# MicroRNA‐Enriched Plant‐Derived Exosomes Alleviate Colitis by Modulating Systemic Immunity, Metabolic Homeostasis, and Gut Microbiota

**DOI:** 10.1002/advs.202505921

**Published:** 2025-09-24

**Authors:** Ruipeng Shi, Wen Tan, Haochun Jin, Sio I Chan, Wei Li, Si San Lei, Guozhen Cui, Yitao Wang, Dong‐Hua Yang, Zhangfeng Zhong

**Affiliations:** ^1^ Macao Centre for Research and Development in Chinese Medicine State Key Laboratory of Quality Research in Chinese Medicine Institute of Chinese Medical Sciences University of Macau Macao SAR 999078 China; ^2^ School of Pharmacy Lanzhou University Lanzhou 730000 China; ^3^ Modern Research Center for Traditional Chinese Medicine Beijing Research Institute of Chinese Medicine Beijing University of Chinese Medicine Beijing 102401 China; ^4^ Department of Bioengineering Zhuhai Campus of Zunyi Medical University Zhuhai 519041 China; ^5^ New York College of Traditional Chinese Medicine Mineola New York 11501 USA

**Keywords:** Centella Asiatica, colitis, microRNA, plant‐derived exosomes, single‐cell sequencing

## Abstract

Ulcerative colitis (UC) is a condition with complex immune dysregulation and chronic intestinal inflammation. Currently, there are still a lack of natural and low‐toxicity therapeutic approaches for UC. Emerging research has underscored the potential of plant‐derived exosomes for UC treatment. In this study, it is found that Centella Asiatica‐derived exosomes (CAEs) have anti‐colitis effects. CAEs selectively accumulate in inflamed colons, enabling targeted delivery of RNA cargos. MicroRNA (miRNA)‐enriched CAEs not only mitigate inflammation but also facilitate the reconstitution of a balanced gut microbiota, characterized by a deceased abundance of pathogenic bacteria (e.g., *Salmonella enterica*), as well as regulating 880 serum metabolites. Furthermore, the results indicate the remarkable impact of specific miRNAs from CAEs, such as *aof‐miR396b* and *fve‐miR396c‐3p*, on *Peak1* target. CAEs attenuate inflammatory responses and enhance the functions of immune cells in the intestinal milieu. Importantly, the safety profile of CAEs is exemplary, with no discernible adverse effects observed in both in vitro and in vivo assays. This study posits plant‐derived exosomes as a potent, targeted, and safe therapeutic modality for UC, representing an alternative to conventional treatments by leveraging the inherent bioactive properties of botanically derived nanovesicles and the cross‐kingdom regulatory potential of plant‐derived miRNAs on mammalian genes.

## Graphical abstract

Herbal medicine, with its multicomponent and multitarget potential, is often studied primarily for traditional forms, especially phytochemicals. Currently, ulcerative colitis lacks natural, low‐toxicity therapies, yet emerging research highlights plant‐derived exosomes as a promising option. Centella Asiatica‐derived exosomes (CAEs), a novel form rich in miRNAs, alleviate colitis by modulating immunity, metabolism, and microbiota, safely targeting inflamed colons as a viable therapy.

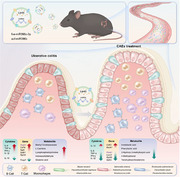

## 1 Introduction

Ulcerative colitis (UC) is a recalcitrant form of inflammatory bowel disease (IBD), characterized by chronic intestinal inflammation involving a complex interplay of immune dysregulation and genetic factors.^[^ ^1^ ^]^ The current therapeutic strategies for UC management include amino‐salicylic acids (ASA), glucocorticoids, immunosuppressants, and advanced biological therapies. These interventions are tailored to target specific proinflammatory and downstream signaling pathways; however, the nonspecific nature of these pharmacological agents can elicit a range of adverse effects.^[^ ^2^ ^]^

UC is intricately associated with dysbiosis of the gut microbiota within the colonic microenvironment. Dysbiosis is characterized by a diminished diversity of the microbiota and is implicated in the induction of chronic inflammatory states with heightened production of toxic metabolites, and disruptions in the host's metabolic balance.^[^ ^3^ ^]^ Specific intestinal bacteria can utilize nutrients and signaling molecules from exosomes, which in turn augment their growth and metabolic activities.^[^ ^4^ ^]^ Furthermore, exosomes harboring microRNAs (miRNAs) have demonstrated the capability to modulate gut microbiota, exerting influences on systemic functions, including metabolic processes.^[^ ^5^ ^]^

The advent of nanotechnology has ushered in a new era of targeted therapeutics, with exosomes, the nano‐sized membrane vesicles secreted into the extracellular space, garnering significant interest.^[^ ^6^ ^]^ Exosomes derived from natural sources, particularly those from plants, demonstrate reduced immunogenicity and possess an inherent safety profile,^[^ ^7^ ^]^ when compared to exosomes produced by animal cells.^[^ ^8^ ^]^ Recent studies have demonstrated the broad potential of plant‐derived exosomes in the field of biomedicine, highlighting the multifunctionality of exosomes in drug delivery, intercellular communication and therapeutic interventions for disease treatment.^[^ ^8^, ^9^ ^]^ Several plant‐derived exosome‐like nanoparticles have been identified and reported to facilitate interspecies communication and possess potential anti‐cancer and anti‐inflammatory properties.^[^ ^10^ ^]^ These exosomes encapsulate a plethora of bioactive molecules, such as metabolites, proteins, lipids, and nucleic acids such as miRNAs, are adept at delivering these molecules directly to sites of inflammation, enhancing the precision and potency of therapeutic interventions. The lipid and protein composition of the exosome membrane not only facilitates cellular uptake and transport but also ensures complete degradation, thereby minimizing side effects and toxicity.^[^ ^11^ ^]^

MicroRNA was first identified in *C*. elegans in 1993, such as lin‐4 miRNA. This breaking discovery was honored with the Nobel Prize in Physiology or Medicine in 2024.^[^ ^12^ ^]^ MicroRNA is a class of endogenous RNA with 20–24 nucleotides. MicroRNAs are noncoding single strands with crucial roles in the regulation of gene expression, which are associated with various diseases.^[^ ^13^ ^]^ Abundant miRNAs have been discovered in plants. Plant‐derived miRNAs could be transferred into animal cells and regulate their gene expression. They have the potential for therapeutic applications, such as anti‐inflammation, anti‐cancer, anti‐virus, and anti‐obesity.^[^ ^14^ ^]^ For example, ginger‐derived *osa‐miR164d* exerts anti‐colitis effects by silencing TAB1, a mediator of the TNF pathway, driving macrophage polarization towards the anti‐inflammatory M2 phenotype and suppressing pro‐inflammatory cytokines.^[^ ^15^ ^]^

**Figure 1:**
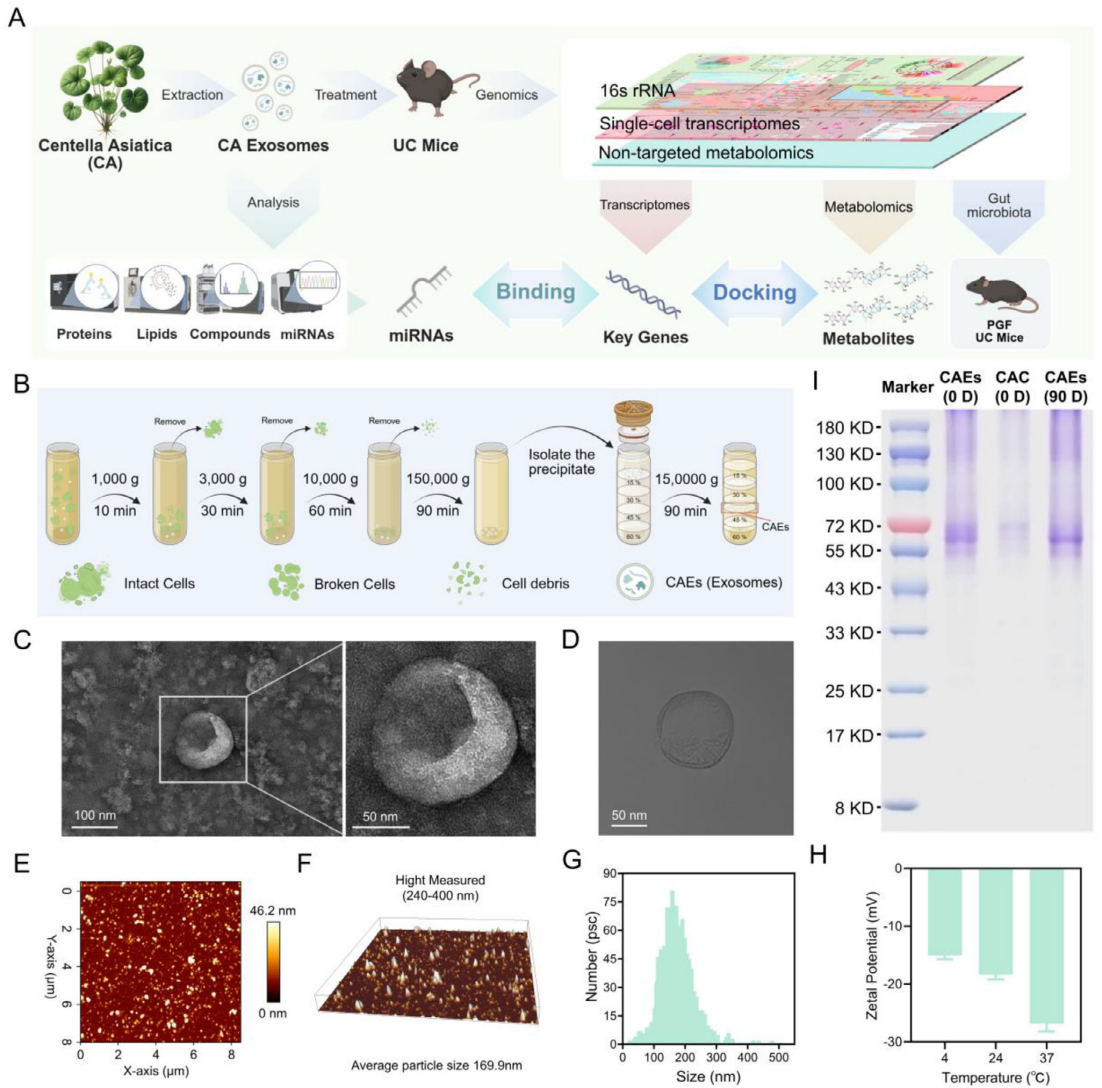
Isolation and characterization of Centella Asiatica‐derived exosomes (CAEs). A) Schematic representation of the research methodology. B) The selective isolation and purification of CAEs. C) Transmission electron microscope (TEM) images of CAEs. D) Cryo‐TEM analysis of CAEs. E) Atomic force microscopy (AFM) topographical analysis of CAEs. F) Three‐dimensional AFM visualization of CAEs surface. G) Size distribution profiles of CAEs determined by nanoparticle tracking analysis (NTA). H) Zeta potential of CAEs across varying temperatures evaluated by dynamic light scattering (DLS). I) Sodium dodecyl sulfate‐polyacrylamide gel electrophoresis (SDS‐PAGE) analysis for the proteins within CAEs, and the symbol D denotes Day, CAC was abbreviated for Centella asiatica crushing solution. The data were representative of three independent experiments.

Herbal medicine exhibits anti‐inflammatory potential, whether in their conventional extracts or innovative forms.^[^ ^16^ ^]^ Centella Asiatica^[^ *Centella asiatica* (L.) Urb.] with its principal constituents Asiatic acid and Madecassic acid^[^ ^17^ ^]^ have exhibited therapeutic potential, such as anti‐inflammation,^[^ ^18^ ^]^ anti‐cancer,^[^ ^19^ ^]^ and neuroinflammation‐attenuating properties.^[^ ^20^ ^]^ Dietary interventions that alter gut microbiota have been shown to be able to prevent and treat IBD, including UC.^[^ ^21^ ^]^ Given the enrichment of bioactive constituents within plant‐derived exosome‐like nanoparticles, we hypothesized that a combination strategy utilizing plant‐derived exosomes, leveraging their potent bioactive components and their inherent ability to modulate gut flora, could offer a distinct advantage in the treatment of colitis (**Figure** **1**A).

In the present study, we investigated the exosome‐like nanovesicles isolated from fresh Centella Asiatica (Centella Asiatica‐derived exosomes, CAEs). The bioactive constituents of CAEs were characterized through LC‐MS, lipidomics, proteomics, and miRNA profiling analyses. These plant‐derived exosomes, notably CAEs, demonstrate significant advantages over conventional therapeutic modalities for UC, particularly regarding cross‐kingdom regulation, targeted delivery, and reduced immunogenicity. For example, CAEs are rich in miRNAs, which not only play a role in cross‐kingdom regulation but also have precisely targeting ability. Moreover, the CAEs targeted delivery capability might attribute to its unique phospholipid bilayer structure, which facilitates their selective delivery to inflamed intestinal tissues via inherent tropism mechanisms. This targeted approach enhances therapeutic efficacy and minimizes off‐target systemic effects, a limitation frequently associated with nonspecific conventional drugs. Critically, CAEs are of natural origin, featuring significantly lower immunogenicity than synthetic nanoparticles or animal‐derived therapeutic agents. Their intrinsic plant origin and biodegradability confer a lower propensity to elicit adverse immune responses, an essential consideration for chronic UC management. Notably, a whole‐process and low‐temperature extraction methodology employed in this study to isolate plant‐derived exosomes from Centella Asiatica is designed to preserve the structural and functional integrity of these bioactive nanovesicles. This approach is similar to the gentle processing conditions used by Tu Youyou (the Nobel Prize winner in Physiology or Medicine in 2015) in the extraction of artemisinin, where the maintenance of therapeutic efficacy for herbal plants is paramount. A DSS‐induced murine model was established to evaluate the in vivo anti‐colitis efficacy of CAEs. Through single‐cell transcriptomics, we elucidated the profound effects of CAEs on colonic epithelial cells and immune cells, revealing a significant change of the immune microenvironment in the colonic tissues. Plasma metabolomic analysis underscored the systemic impact of CAEs on the host metabolic pathways, suggesting a potential rebalancing of disturbed homeostatic mechanisms in UC. Microbiome analysis revealed that CAEs could balance gut microbiota through deceasing abundance of pathogenic bacteria and increasing that of beneficial bacteria. Importantly, our study provides compelling evidence for the cell‐metabolite‐gut microbiota axis as a viable therapeutic target, illustrating the potential of plant‐derived exosomes for IBD treatment.

## 2 Results

### 2.1 Preparation and Physicochemical Characterization of CAEs

CAEs were isolated from fresh Centella Asiatica juice and purified using sucrose gradient ultracentrifugation (Figure 1B). CAEs are mainly distributed in a 30–45% (w/v) sucrose density gradient, with a discernible band evident at the 15–30% and 45–60% interface (Figure S1, Supporting Information), and have a characteristic density range between 1.13 and 1.19 g mL^−1^. The CAEs located at the 30–45% sucrose gradient interface were collected. For morphological examination of CAEs, aliquots of the CAEs suspension were adjusted to an optimal concentration and subjected to negative staining using 1% (w/v) uranyl acetate solution. Transmission electron microscope (TEM) images revealed that CAEs exhibit typical extracellular vesicle morphology with a spherical or cup‐shaped structure and a bilayer membrane (Figure 1C). Moreover, the structural features of CAEs were visualized using a cryo‐transmission electron microscopy (Cryo‐TEM) (Figure 1D). Under TEM and Cryo‐TEM, CAEs exhibited a characteristic of rounded, hollow vesicular morphology.

Topographical analysis of CAEs surface was detected using atomic force microscopy (AFM). The distribution of white spots across the surface suggests a uniform spherical morphology with smooth surface for CAEs (Figure 1E). Three‐dimensional AFM imaging further confirmed the round vesicle‐like structure of CAEs (Figure 1F). The average particle size was approximately 169.9 nm, consistent with the typical size range of plant‐derived extracellular vesicles. The concentration and diameter of purified CAEs were measured using nanoparticle tracking analysis (NTA). Video S1 (Supporting Information) illustrates the flow of CAEs, which showed a concentration of 6.3 × 10^11^ particles mL^−1^ and a mean diameter of 162.3 nm (Figure 1G). The zeta potential measurements of purified CAEs were conducted using dynamic light scattering (DLS) in PBS solution at 4 °C, 24 °C, and 37 °C, yielding values of −15.03, −18.33, and −26.83 mV, respectively (Figure 1H). Gel electrophoresis analysis revealed that most proteins encapsulated in CAEs exhibited molecular weights concentrated in the range of approximately 55–72 kilodaltons (kDa), and these CAEs demonstrated stability when stored for 90 days at −80 °C (Figure 1I). Regarding protein content, the yield from the CAEs interface was determined to be approximately 50 mg/1 kg of fresh Centella Asiatica. CAEs is a concentrated form obtained through ultracentrifugation‐based extraction, as compared with Centella Asiatica crushing solution (CAC). To compare the bioactive constituents between CAEs and other components, the quantification of CAEs was conducted by assessing the protein concentration.

### 2.2 Molecular Characterization of CAEs

**Figure 2:**
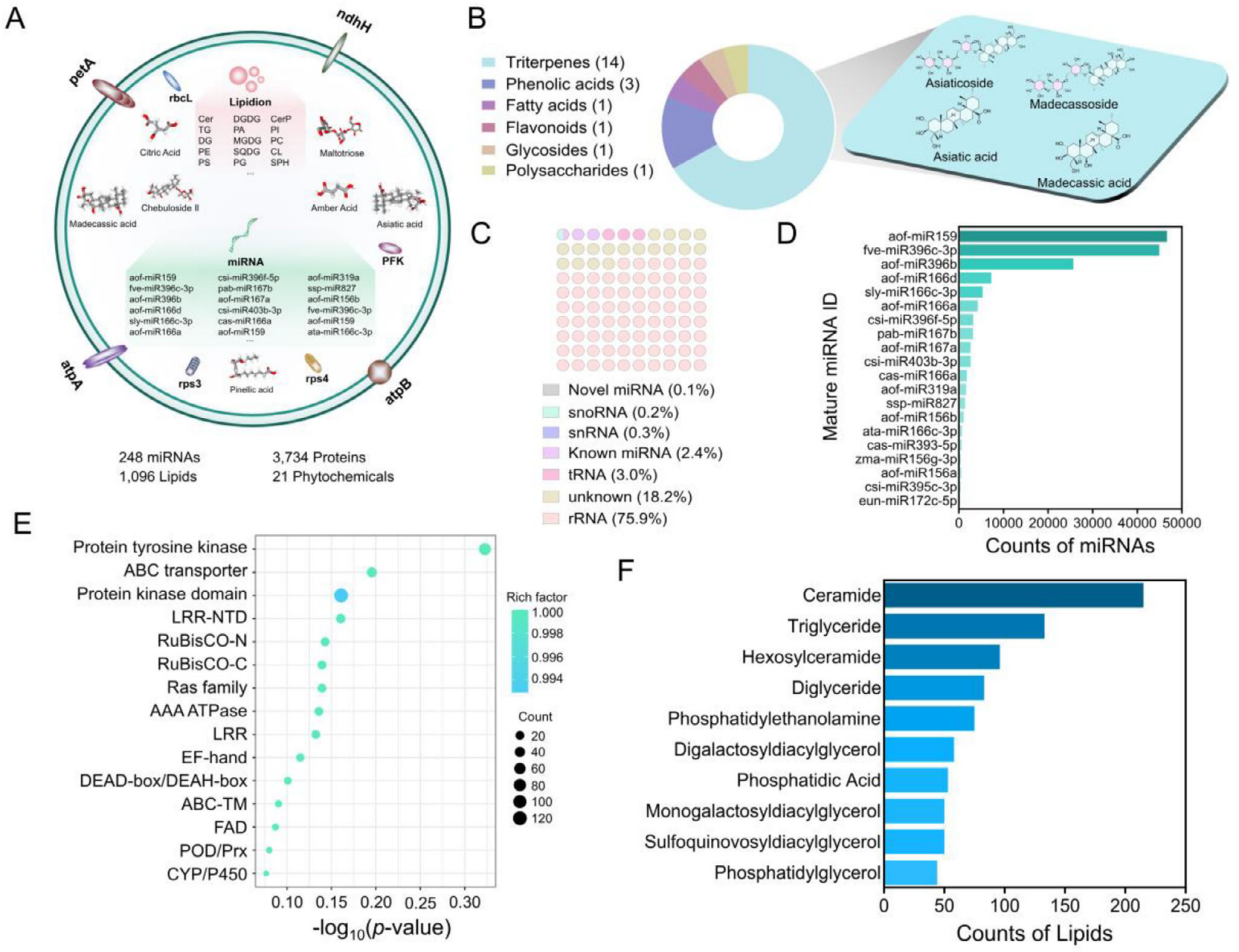
Component identification of Centella Asiatica‐derived exosomes (CAEs). A) Characterization of bioactive constituents, including RNAs, lipids, proteins, and phytochemicals in CAEs. B) Ultra‐high‐performance liquid chromatography coupled with quadrupole time‐of‐flight mass spectrometry (UHPLC‐Q‐TOF/MS) based classification of phytochemicals in CAEs. C) RNA sequencing analysis for the classification of RNAs in CAEs. D) Top 20 miRNAs identified in CAEs. E) Proteomic‐driven Kyoto Encyclopedia of Genes and Genomes (KEGG) enrichment analysis of CAEs proteins. F) Lipidomic profiling at the class level for CAEs lipids.

Exosomes carry a variety of endogenous substances derived from their parental cells. We conducted a comprehensive analysis of exosomal cargos, including an array of metabolites (phytochemicals), RNAs, proteins, and lipids (**Figure** **2**A). A total of 21 phytochemicals were identified, encompassing 14 triterpenes, 3 phenolic acids, 1 fatty acid, 1 flavonoid, 1 glycoside, and 1 polysaccharide, with the characteristic compounds such as Asiaticoside, Asiatic acid, Madecassoside, and Madecassic acid (Figure 2B, Figure S2, and Table S1, Supporting Information). Recognizing the pivotal role of exosomes in intercellular communication, we focused on miRNAs, which are stable, conserved, and biologically active nucleic acids. miRNAs were sequenced to comprehend the molecular profile of the CAEs. The sequencing data revealed that most of the nucleic acid content consisted of ribosomal RNAs (rRNAs, 75.9%), transfer RNAs (tRNAs, 3.0%), known miRNAs (2.4%), small nuclear RNAs (snRNAs, 0.3%), and small nucleolar RNAs (snoRNAs, 0.2%), with a minor fraction of novel miRNAs accounting for 0.1% (Figure 2C). Notably, the miRNAs, *aof‐miR159*, *fve‐miR396c‐3p*, and *aof‐miR396b*, were identified as the most abundant, representing the highest proportion of the miRNA profile (Figure 2D and Table S2, Supporting Information). The most notable feature of exosomes is their phospholipid bilayer structure. In our study, we performed a thorough proteomic and lipidomic analysis of CAEs cataloged a comprehensive list of 3734 proteins derived from CAEs and subjected the proteins to Kyoto Encyclopedia of Genes and Genomes (KEGG) pathway enrichment analysis. Notable presence of proteins involved in key biological processes such as protein tyrosine and serine/threonine kinase, as well as ABC transporters and protein kinase domains were notably present (Figure 2E, Figure S3, and Table S3, Supporting Information). These findings suggest that CAEs, akin to many reported exosomes, are predominantly implicated in intercellular communication. Paralleling to our proteomic investigation, we conducted a lipidomic study that identified 1096 distinct lipid species within the CAEs (Table S4, Supporting Information). The lipid composition was notably rich in ceramide, which accounted for 19.6% of the total lipid content, followed by triglyceride at 12.1%, and hexosylceramide at 8.8% (Figure 2F). The detailed characterization of both the proteome and lipidome of CAEs provides a deeper understanding of their molecular architecture and potential functional roles in biological systems.

### 2.3 In Vitro Cellular Uptake and In Vivo Distribution of CAEs

**Figure 3:**
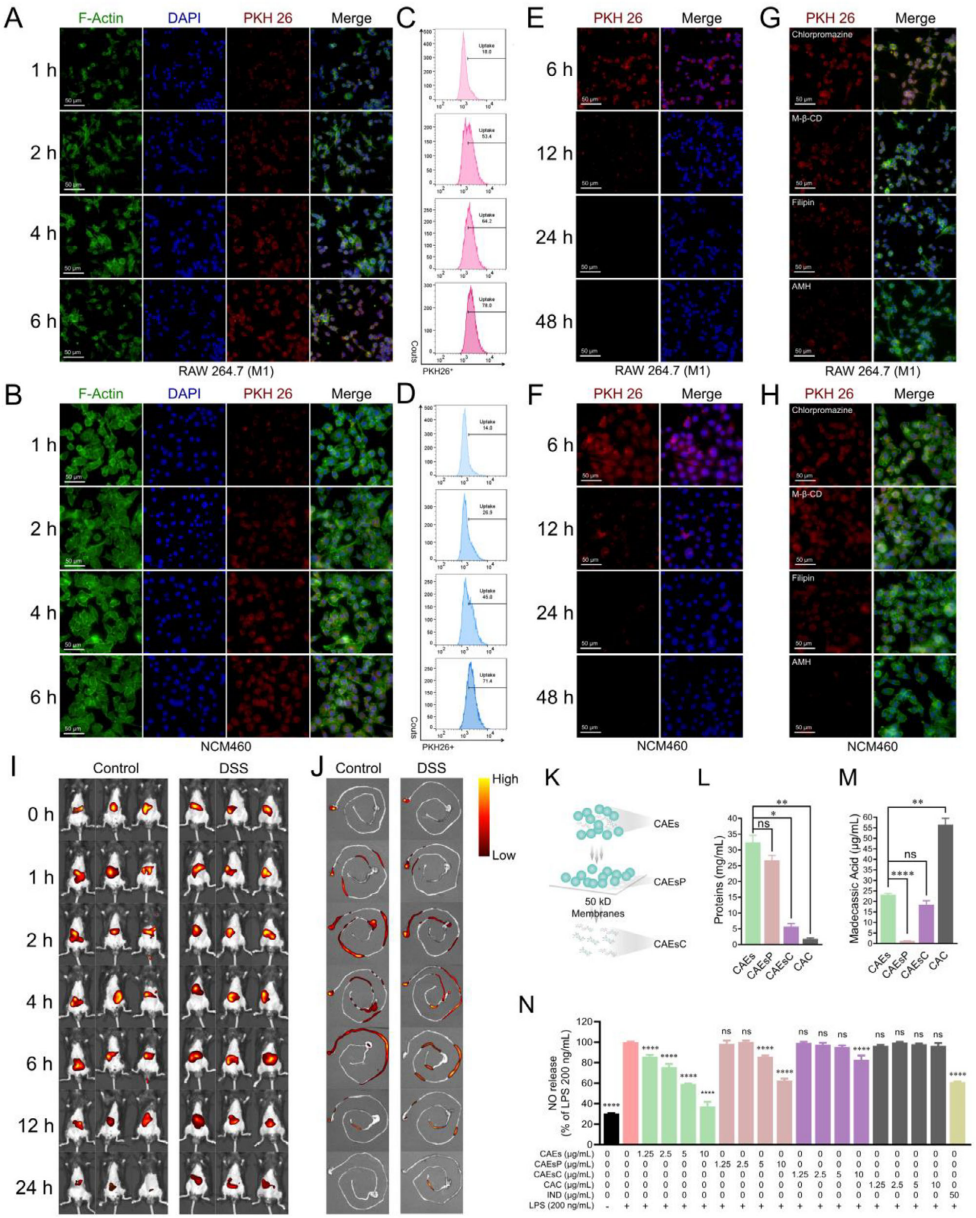
In vitro and in vivo characterization of cellular uptake and retention of Centella Asiatica‐derived exosomes (CAEs). A) Representative fluorescence microscopy images depicting the time‐dependent cellular uptake of CAEs in RAW 264.7 cells and B) NCM460 cells at 1‐, 2‐, 4‐, and 6‐h incubation (These histogram bars served as a negative reference for gating PKH 26‐positive cells). C) Flow cytometric analysis was used to monitor the cellular uptake of CAEs in RAW 264. 7 cells and (D) NCM460 cells. E) Representative fluorescence microscopy images of CAEs retention in RAW 264.7 cells and F) NCM460 cells after an initial 6‐h incubation, followed by a chase period with fresh medium for 6, 12, 24, and 48 h. G) Effects of chlorpromazine, methyl‐β‐cyclodextrin (M‐β‐CD), filipin, and amiloride hydrochloride (AMH) inhibitors on the uptake efficiency of CAEs in RAW 264.7 cells and H) NCM460 cells after 6‐h treatment. I) Whole‐body imaging from control mice and DSS‐induced acute colitis mice at 0‐, 1‐, 2‐, 4‐, 6‐, 12‐, and 24‐h post‐oral administration of DiR‐labeled CAEs. J) Corresponding gastrointestinal distribution of DiR‐labeled CAEs in control and DSS‐colitis mice at indicated time points. K) The fractionation of CAEs constituents into their respective compounds and proteins, the protein fraction (CAEsP) and the compound fraction (CAEsC). L) Quantitative assessment of protein content post‐separation. M) Quantitative analysis of Madecassic acid content post‐separation, a typical compound in CAEs. N) NO release was quantified following an incubation of macrophages with CAEs, CAEsP, CAEsC, and Centella asiatica crushing solution (CAC) at varying concentrations (1.25, 2.5, 5, and 10 µg mL^−1^), as well as indomethacin (IND) (50 µg mL^−1^), with or without the addition of LPS (200 ng mL^−1^) for a duration of 24 h. The data were representative for three independent experiments. Statistical significance was indicated by ^*^ *p* < 0.05, ^**^ *p* < 0.01, ^***^ *p* < 0.001, and ^****^ *p* < 0.0001, no statistical significance was indicated by ns.

Cellular uptake is a pivotal mechanism by which drug delivery systems exert their therapeutic effects. To trace the cellular uptake of CAEs, CAEs were fluorescently labeled with a red dye PKH 26 and co‐incubated with macrophages and normal colonic epithelial cells. A distinct increase in red fluorescence was observed within RAW 264.7 and NCM460 cells over time, indicating the internalization of CAEs (**Figure** **3**A,B). Notably, fluorescence with high intensity was sustained for up to 6 h post‐incubation in both cell lines. Further quantitative analysis using flow cytometry demonstrated an enhancement in the fluorescence signal of RAW 264.7 cells, increasing from 18.0% to 78.0% for 1‐ and 6‐h incubation, respectively (Figure 3C). Similarly, the fluorescence signal of NCM460 cells increased from 14.0% to 71.4% (Figures 3D and S4, Supporting Information). However, after a 12‐h post‐incubation period, there was a gradual decline in red fluorescence intensity within both cell types (Figure 3E,F). This attenuation is hypothesized to be due to the fluidic nature of the cell membrane and the phagocytic activity of RAW 264.7 and NCM460 cells, which may facilitate the degradation and clearance of the internalized CAEs. Confocal microscopy analysis showed a marked reduction in red fluorescence intensity in both cell types after treatment with chlorpromazine, methyl‐β‐cyclodextrin (M‐β‐CD), and filipin, suggesting the involvement of clathrin‐mediated and caveolae/lipid raft‐dependent endocytosis pathways in CAEs uptake. Notably, amiloride hydrochloride (AMH) showed a more potent inhibitory effect, indicating phagocytosis or macropinocytosis, mediated by the membrane sodium channel, plays a critical role in the internalization of CAEs (Figure 3G,H). Flow cytometry analysis further validated these findings (Figure S5, Supporting Information), suggesting that CAEs are primarily internalized via membrane sodium channel‐, lipid raft‐ and clathrin‐mediated pathways in both immune and intestinal epithelial cells.

To assess the targeting capability of CAEs in vivo, fluorescence intensity was monitored following the oral administration of CAEs labeled with the fluorescent marker. At various points post‐gavage (0,1, 2, 4, 6, 12, and 24 h), the mice were euthanized, and colon tissues were collected for fluorescence imaging using an IVIS Spectrum Series imaging system. As shown in Figure 3I, the fluorescence intensity of CAEs in mice peaked at 2 h post‐administration, indicating a rapid accumulation at the target site following intragastric delivery. Thereafter, the fluorescence signal gradually diminished, becoming undetectable by 24 h, suggesting a clearance of the CAEs from the systemic circulation, only partial retention of CAEs was observed in the colonic tissues of DSS‐induced acute colitis mice. Ex vivo biodistribution analysis of CAEs in the collected colon tissues corroborated with the in vivo fluorescence imaging results, demonstrating consistent fluorescence intensity (Figure 3J). The biodistribution of 1,1′‐dioctadecyl‐3,3,3′,3′‐tetramethylindotricarbocyanine iodide (DiR)‐labeled CAEs was assessed in major organs of control and DSS‐induced colitis mice at multiple time points following oral administration. At 2 h post‐administration, a strong DiR fluorescence signal was predominantly detected in the liver, indicating initial hepatic accumulation. After 4 h, the fluorescence signal shifted primarily to the lung, suggesting redistribution or secondary uptake. At 6 h, weak residual signals were still observed in both the liver and lung, indicating partial retention of CAEs in these organs (Figure S6A, Supporting Information). ELISA analysis of the plant‐derived protein RbcL revealed that, following oral administration of CAEs, RbcL was primarily distributed in the stomach, small intestine, and colon (Figure S6B, Supporting Information). Collectively, these observations suggest that CAEs demonstrate a propensity for selective accumulation at sites of colonic inflammation, highlighting their potential as targeted carriers for colonic diseases. To elucidate the active components within CAEs, we fractionated them into the exosomal protein (CAEsP) and the exosomal compounds (CAEsC) in Figure 3K. In detail, 82.43% of the total protein was selectively retained within the CAEsP fraction, whereas 17.57% was found in the CAEsC fraction. In contrast, a lower proportion of 5.40% of the total compounds was retained in the CAEsP fraction, with the majority, 79.81%, being present in the CAEsC fraction. It is intuitive to observe that our established separation method separates the proteins and compounds of CAEs, as evidence by the content quantification of proteins, miRNAs, madecassic acid and asiatic acid within the CAEsP, CAEsC, and CAC fractions (Figure 3L,M, Figures S7 and S8, Supporting Information). CAEsP exerted a pronounced anti‐inflammatory effect in lipopolysaccharide (LPS) induced RAW 264.7 macrophages, suggesting that protein and miRNAs, rather than phytochemicals, are the predominant mediators of the anti‐inflammatory activity of CAEs, as shown in Figure 3N. CAEs at a concentration of 10 µg mL^−1^ did not induce cytotoxicity in RAW 264.7 cells (Figure S9A, Supporting Information) and the macrophage polarization from the M0 to the M1 state, which is characterized by a proinflammatory phenotype, is attenuated by CAEs, CAEsP, and CAEsC (Figure S9, Supporting Information).

### 2.4 Therapeutic Efficacy of CAEs in DSS‐Induced Colitis Model in Mice

**Figure 4:**
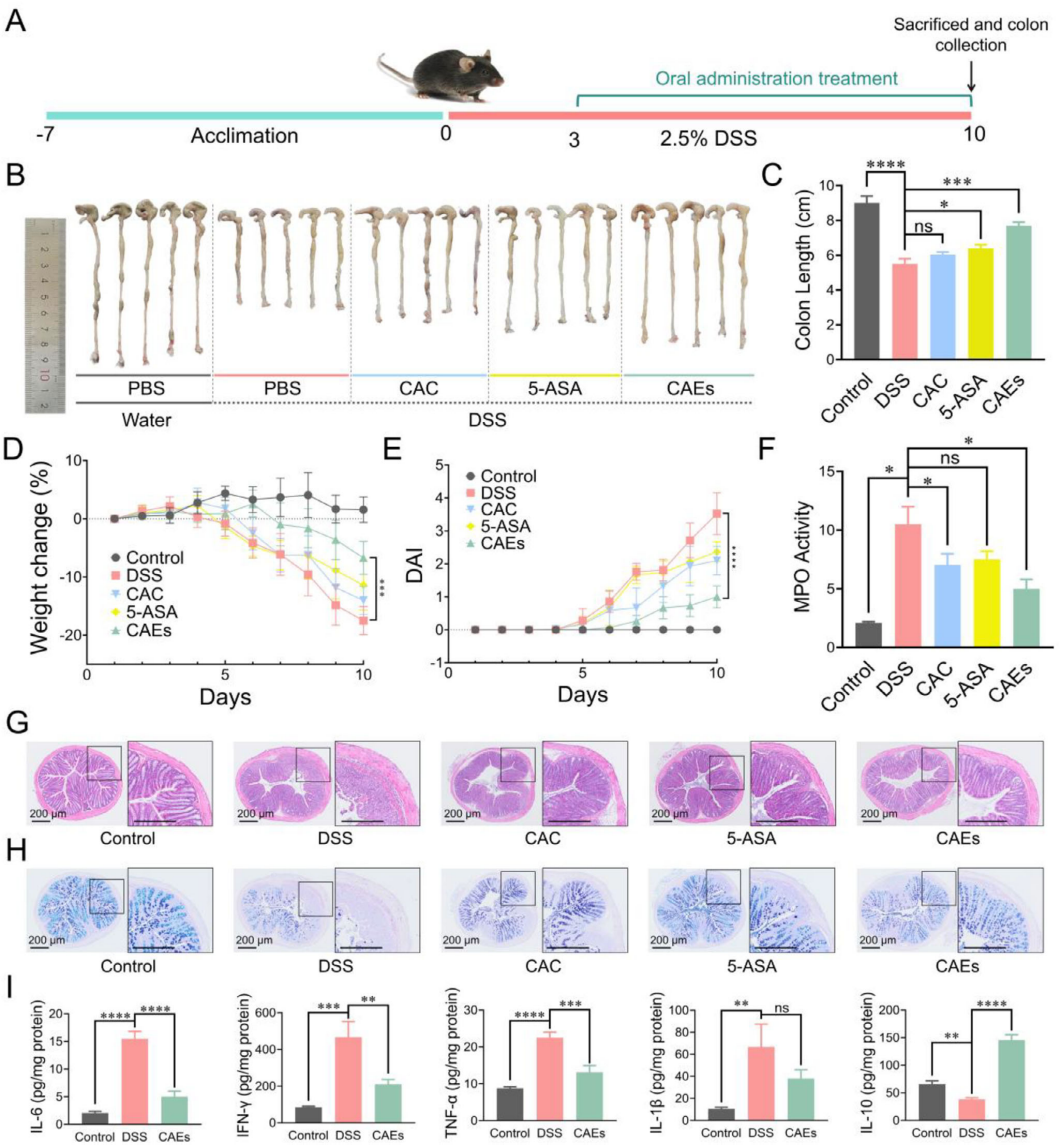
Anti‐colitis effects of Centella Asiatica‐derived exosomes (CAEs) in DSS‐induced acute murine model. A) The schematic flowchart delineates the methodology for inducing colitis and the subsequent treatment protocol. B) Visual representation of the colon morphology after treatment withCentella Asiatica crushing (CAC) and CAEs. C) Quantification of colon length. D) Body weight monitoring throughout the study. E) Assessment of disease activity index (DAI) to quantify the dynamic expression of colitis symptoms. F) Determination of myeloperoxidase (MPO) activity (U g^−1^) in the colonic tissue. G) Histological examination of the distal colons with hematoxylin‐eosin (H&E) staining. H) Staining of the distal colons with AB/PAS. I) Quantitative analysis of pro‐inflammatory (IL‐6, IFN‐γ, TNF‐α, and IL‐1β) and anti‐inflammatory (IL‐10) cytokines expression levels in the colon tissues. Data were presented as mean ± SD, with *n* = 6 animals per group. Statistical significance was denoted by asterisks, ^*^ *p *< 0.05, ^**^ *p* < 0.01, ^***^ *p *< 0.001, and ^****^ *p *< 0.0001, no statistical significance was indicated by ns.

The experimental paradigm involved the induction of acute colitis in mice through the administration of 2.5% dextran sulfate sodium (DSS) in drinking water (**Figure** **4**A). The therapeutic efficacy of CAEs, CAC, asiatic acid, asiaticoside, madecassic acid, and madecassoside against UC were investigated in a colitis mouse model in vivo (Figure S10, Supporting Information). We found that CAEs exhibit the strongest anti‐colitis effect compared with CAC, asiatic acid, asiaticoside, madecassic acid, and madecassoside. To further confirm the anti‐colitis effects of CAEs, the mice were randomly assigned into the following cohorts, including the Negative Control (PBS + Water), Disease Model (PBS + DSS), CAC Treatment (Juice + DSS) and CAEs Treatment (Exosomes + DSS), and Positive Control (5‐aminosalicylic acid: 5‐ASA + DSS). The treatment regimen involved daily oral gavage administration of either PBS, CAC, 5‐ASA, or CAEs. The therapeutic efficacy of different treatments was evaluated through a series of established metrics, including monitoring the body weight fluctuations, calculation of the disease activity index (DAI), measurement of colon lengths, quantification of pro‐ or anti‐inflammatory cytokines (IL‐6, IFN‐γ, TNF‐α, IL‐1β, and IL‐10) in colonic tissues, and histological examination of colon tissues. Colon shortening, a hallmark indicator of inflammatory severity, was assessed alongside the DAI, which quantitatively mirrors the clinical symptom of colitis. Additionally, the activity of myeloperoxidase (MPO), a pivotal biomarker for neutrophil infiltration into colonic tissues during intestinal inflammation, was measured as an index of inflammatory cell migration and activity. Colon lengths in the Control, DSS, CAC, 5‐ASA, and CAEs groups were measured at 9.0 ± 0.4, 5.5 ± 0.3, 6.0 ± 0.2, 6.4 ± 0.2, and 7.7 ± 0.2 cm, respectively (Figure 4B,C). Compared with the DSS‐induced group, the CAEs treatment showed an elongation of the colon, a higher average body weight (Figure 4D), a reduced DAI (Figure 4E), and diminished MPO activity (Figure 4F), indicating a mitigated inflammatory response. Histological assessments of colon tissues were conducted via hematoxylin‐eosin (H&E) staining and Alcian Blue/Periodic Acid‐Schiff (AB/PAS). In the colitis mice, there was pronounced crypt destruction, substantial immune cell infiltration, and colonic epithelial damage. In contrast, the colon tissues with CAEs treatment displayed a near‐normal histological microstructure with minimal inflammatory cell infiltration (Figure 4G,H). Collectively, these findings suggest that CAEs effectively ameliorate the pathophysiological conditions associated with IBD. Additionally, the DSS treatment exhibited elevated levels of pro‐inflammatory cytokines (IL‐6, IFN‐γ, TNF‐α, and IL‐1β) compared to healthy mice (Figure 4I). Orally administered CAEs led to a marked reduction in the secretion of these cytokines. Notably, IL‐10, a prominently recognized anti‐inflammatory cytokine, showed significantly higher levels in the CAEs treatment than the DSS‐treated group, suggesting a regulatory effect of CAEs on the immune response.

### 2.5 The Underlying Mechanism of Anti‐Colitis Effects of CAEs Revealed by Single‐Cell Transcriptome

**Figure 5:**
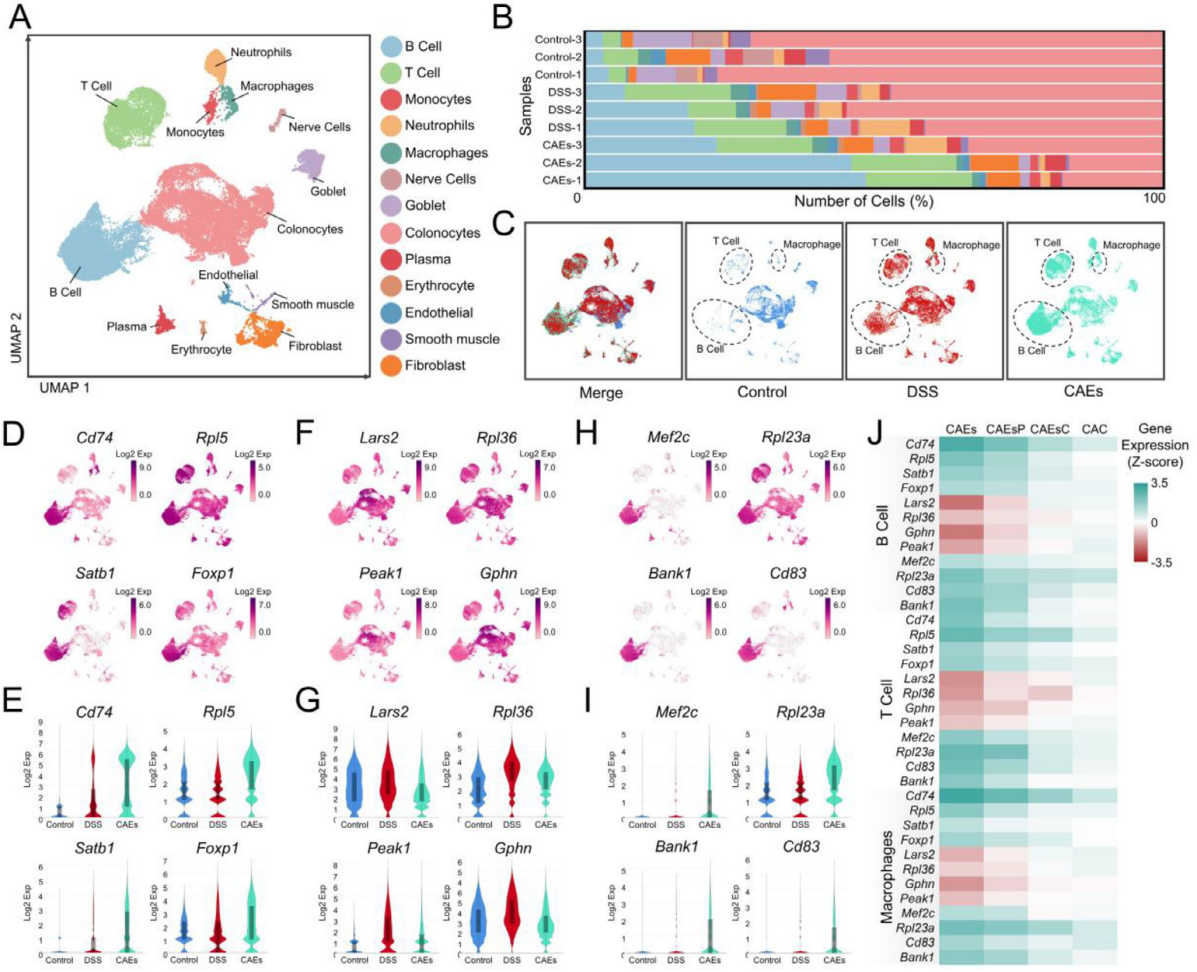
Effect of Centella Asiatica‐derived exosomes (CAEs) on the transcriptional landscape of colonic cells with single‐cell RNA sequencing. A) An analytical quantification of the cellular composition across distinct cell types within the murine colonic tissues by uniform manifold approximation and projection (UMAP) for the dimensionality reduction and clustering. B,C) UMAP algorithm used to classify and visualize cellular clusters, represented Control, DSS‐induced, and CAEs‐treated groups. D–I) Violin plots illustrate the distribution and statistical significance of gene expression variance in DEGs from UMAP clustering analysis. J) Validation of the modulatory effects of CAEs on the expression levels of DEGs in primary B cells, T cells, and macrophages. Data were presented as mean ± SD, with *n* ≥ 3 biological replicates per condition.

Single‐cell transcriptome provides insights into gene expression at the individual cellular level, facilitating the identification of gene regulatory networks and elucidating the variability in gene expression across diverse cell types or states. In our study, we isolated a substantial number of cells from the colon of each group, including 8185 cells from that of Negative Control mice, 13 947 cells from that of DSS‐induced mice, and 21 919 cells from that of CAEs‐treated mice. The cellular heterogeneity was explored using unsupervised uniform manifold approximation and projection (UMAP) clustering, which delineated 13 distinct cell types, including B cells (Figure S11, Supporting Information), T cells (Figure S12, Supporting Information), Goblet cells (Figure S13A, Supporting Information), monocytes, neutrophils, macrophages, and several others (**Figure** **5**A). In the Negative Control group, the distribution of immune cells was as follows: 311 of B cells in total (3.80%), 277 of T cells in total (3.38%) and 33 of macrophages in total (0.40%). In contrast, the DSS‐induced colitis mouse model exhibited an increase in immune cell populations, with 2244 of B cells in total (16.09%), 2010 of T cells in total (14.41%), and 306 of macrophages in total (2.19%). Post‐treatment with CAEs, there was a substantial change in immune cell numbers, with 8771 of B cells in total (40.02%), 3894 of T cells in total (17.77%), and 355 of macrophages in total (1.62%), suggesting that CAEs have the potential to activate immune cells, as evidenced by their increased counts (Figure 5B,C). We identified three distinct categories of genes with expression changes in immune cells following CAEs treatment. Notably, the expression levels of *Cd74*, *Rpl5*, *Satb1*, and *Foxp1* genes were upregulated by DSS or CAEs treatment (Figure 5D,E), while that of *Lars2*, *Rpl36*, *Peak1*, and *Gphn* were upregulated by DSS but attenuated by CAEs treatment (Figure 5F,G, Supporting Information). Moreover, there were some genes exhibiting elevated expression levels exclusively within the CAEs treatment, which were not observed in the DSS‐induced group, including *Mef2c*, *Rpl23a*, *Bank1*, and *Cd83* genes (Figure 5H,I, Supporting Information). Additionally, we specifically focused on gene expression profiles related to goblet cells and intestinal barriers (MUCs and MMPs family). CAEs could attenuate the expression levels of *MUC‐2*, *MUC‐3A*, *MUC‐4*, *MUC‐13*, *MMP‐2*, *MMP‐3*, *MMP‐8*, and *MMP‐9* genes induced by DSS (Figure S13B, Supporting Information). Concurrently, we confirmed some differentially expressed genes (DEGs) in primary cells using quantitative qPCR. These transcriptional changes may be attributed to the complex interplay of proteins, miRNAs, and lipids encapsulated within the CAEs (Figure 5J).

### 2.6 CAEs Modulate the Metabolites in Serum

**Figure 6:**
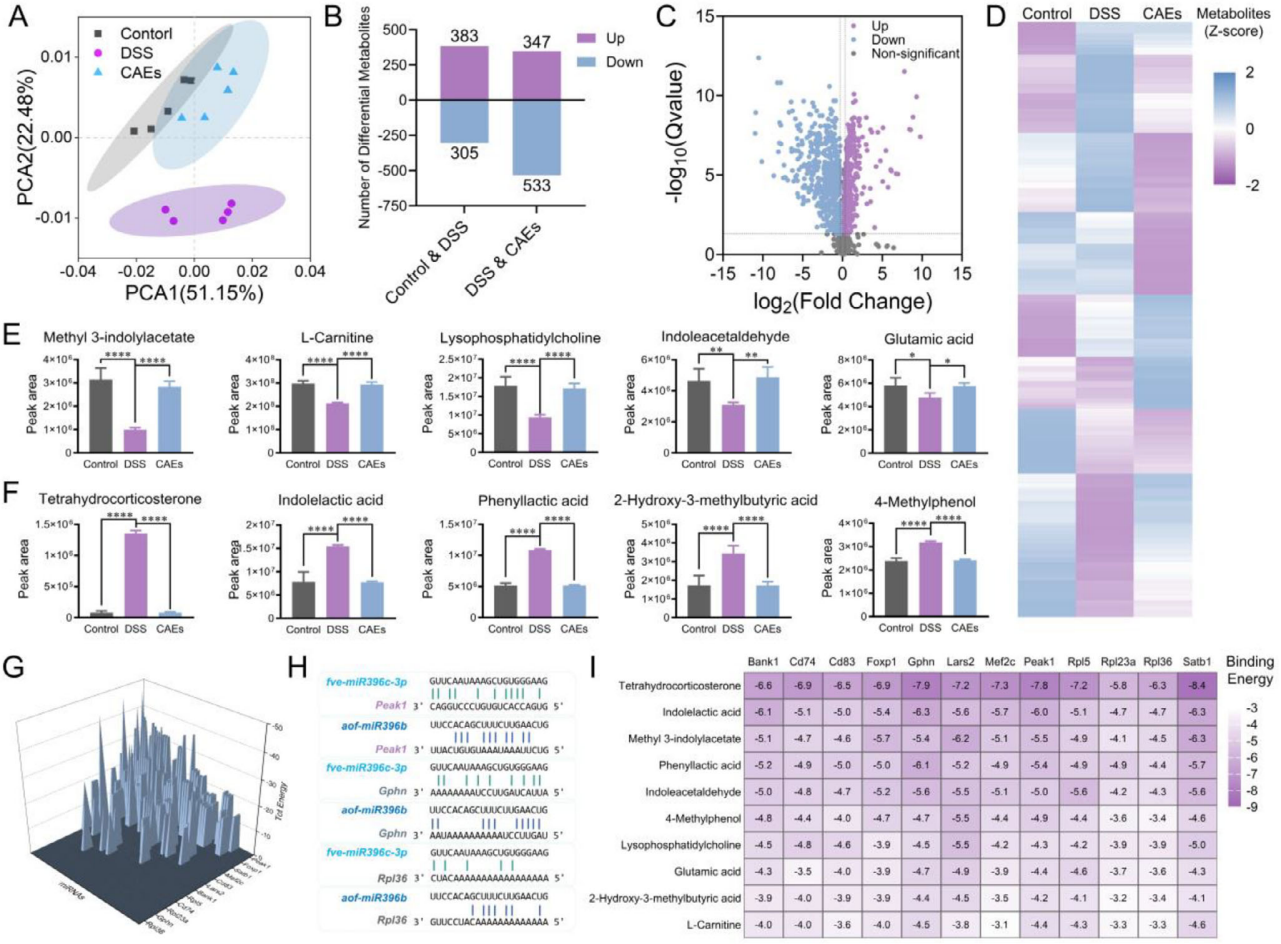
Effects of Centella Asiatica‐derived exosomes (CAEs) on blood metabolomic profiling. A) Principal component analysis (PCA) plot illustrated the metabolic variance and clustering patterns among the experimental groups. B) Histogram representation of serum metabolite distribution across the treatment cohorts. C) Volcano plot depicted the statistical significance and fold‐changes of differentially abundant metabolites in response to CAEs treatment. D) Heatmap visualization of the metabolite expression profiles, highlighting the variations in intensity and pattern. E) The metabolites whose levels were elevated by CAEs treatment compared to the DSS‐induced group. F) The metabolites whose levels were reduced by CAEs treatment compared to the DSS‐induced group. G) Estimation of binding energy between CAEs‐derived miRNAs and the corresponding genes. H) The mRNA sequence of the gene with the fragment of the miRNA sequence with the highest number of matching bases. I) Assessment of the docking binding energy of the predominant metabolites with the respective target protein. Data were presented as mean ± SD, with *n* ≥ 3. Statistical significance was denoted by asterisks, ^*^ *p* < 0.05, ^**^ *p *< 0.01, and ^****^ *p *< 0.0001, signifying the extent of the observed effects.

Principal component analysis (PCA) was used to discriminate serum metabolites among the negative control, DSS‐induced, and CAEs‐treated mouse groups. In the PCA plot, the data of negative control was concentrated, that of the DSS‐induced group was deviated, and that of the CAEs‐treated group trended toward the negative control group, showing the regulatory effect of CAEs on metabolites of the DSS‐induced mice (**Figure** **6**A). The data highlighted the substantial metabolic profile variations that reflect the impacts of the differential treatments. The quantification of differential metabolites showed metabolite variability with the DSS treatment, indicating considerable metabolic perturbations (Figure 6B). Among them, the levels of 383 metabolites were elevated, and 305 metabolites were decreased by DSS treatment. Upon CAEs treatment, 347 metabolites showed increased levels, and 533 metabolites exhibited decreased levels. A detailed volcano plot pinpoints key metabolites that are significantly upregulated or down‐regulated, implicating them as potential orchestrators of the biochemical pathways affected by the CAEs treatments (Figure 6C). This metabolic modulation is further visualized through a heatmap, illustrating the expression patterns of these metabolites across treatments, underscoring consistent and significant alterations (Figure 6D). Specifically, bar graphs quantify the levels of crucial metabolites, including methyl 3‐indolylacetate, l‐Carnitine, lysophosphatidylcholine, indoleacetaldehyde, glutamic acid, tetrahydrocorticosterone, indolelactic acid, phenyllactic acid, 2‐hydroxy‐3‐methylbutyric acid, and 4‐methylphenol (Figure 6E,F). These graphical representations emphasize marked disparities in metabolite concentrations that may suggest mechanisms of action or potential biomarkers for evaluating treatment efficacy. Furthermore, we elucidated the regulatory impact of CAEs‐derived miRNAs on 12 genes through performing sequence alignment with miRanda algorithm. These miRNAs show binding affinities to these genes, including *Foxp1*, *Peak1*, *Satb1*, *Mef2c*, *Bank1*, *Cd74*, *Cd83*, *Gphn*, *Lars2*, *Rpl5*, *Rpl23a*, and *Rpl36* (Figure 6G). Subsequent matching analysis was used to exhibit the presence of potential regulatory associations between two important miRNAs and those 12 genes via the database (*rnamtom.net*) established by our group (Figures S14 and S15, Supporting Information). For example, the fragment of *fve‐miR396c‐3p* and *aof‐miR396b* exhibits the potential sequence match with those of *Peak1, Gphn*, and *Rpl36*, as shown in Figure 6H. The interaction of CAEs‐derived miRNAs with these corresponding target mRNAs indicates a crucial role in gene expression regulation, influencing the stability and translational efficiency of specific mRNAs by complementary pairing, thereby inhibiting or promoting the expression of targeted genes. This analysis determined that CAEs‐derived miRNAs are associated with immune cells, potentially influencing their activation and function. Concurrently, 10 important altered serum metabolites were docking with 12 immune cell‐associated proteins that exhibited remarkable binding energy, corroborating the association of metabolites with immune cells (Figure 6I). Moreover, *fve‐miR396c‐3p* and *aof‐miR396b* demonstrated potential anti‐colitis effects in the DSS‐induced colitis mouse model (Figure S16, Supporting Information). RNA sequencing analysis showed that *fve‐miR396c‐3p* and *aof‐miR396b* could suppress the transcriptional level of *Peak1* induced by DSS in mouse colon tissue (Figure S17, Supporting Information) and that induced by LPS in macrophages (Figure S18, Supporting Information).

### 2.7 Modulatory Effect of CAEs on the Gut Microbiota

**Figure 7:**
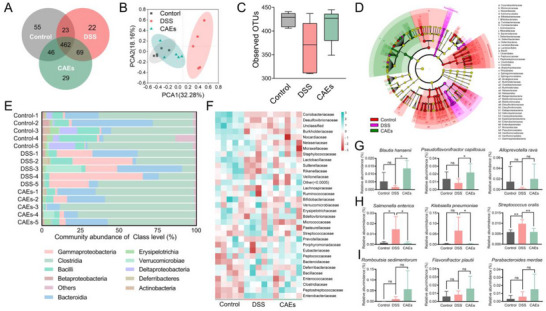
Effects of Centella Asiatica‐derived exosomes (CAEs) on intestinal microbiota assessed through microbiome analysis. A) Operational Taxonomic Units (OTUs) were visualized using a Venn diagram. B) Principal component analysis (PCA) plot depicted the distinct clustering patterns of gut microbiota profiles across different treatment groups. C) Quantitative assessment of OTU richness. D) A circular phylogenetic tree depicting hierarchical relationships and evolutionary distances among identified microbial taxa. E) Comparative analysis of the relative abundance of intestinal microbiota. F) Heatmap of the relative abundance of genus‐level taxonomy for each group of mice. G) Identification and enumeration of representative beneficial microbial species. H) Characterization of harmful microbial taxa at the species level. I) Exploration of potentially beneficial microbial species. Data were presented as mean ± SD, with *n* ≥ 3. Statistical significance was denoted by asterisks, ^*^ *p* < 0.05 and ^**^ *p *< 0.01, no statistical significance is indicated by ns.

Given the capacity of gut microbiota on modulating the immunity of the host, we assessed gut microbiota composition following treatments with PBS and CAEs in DSS‐induced colitis‐bearing mice. Fecal samples were collected and subjected to 16S ribosomal RNA (rRNA) gene sequencing for microbiome analysis. We employed Venn diagrams to illustrate the distribution of operational taxonomic units (OTUs), delineating those common across multiple samples as well as those unique to each group (**Figure** **7**A). The PCA plots revealed that CAEs treatment resulted in a distinct gut microbiota profile when compared with that of DSS‐induced group, showing a convergence with that of Negative Control group (Figure 7B). After CAEs treatment, the observed OTUs indicative of bacterial richness was much greater than those in the DSS‐induced group (Figure 7C). Linear discriminant analysis effect size (LEfSe) analysis showed the dynamics of gut microbial community among Negative Control, DSS, and CAEs groups. It revealed an enrichment of Bifidobacteriales in the Negative Control group and Clostridiales in the CAEs treatment group (Figure 7D). This enhancement in bacterial richness and diversity after CAEs treatment underscores the beneficial influence of CAEs on gut microbiota modulation. An investigation at the class level revealed an increase in the relative abundance of Clostridia in the CAEs treatment group, which was decreased in the DSS‐induced group (Figure 7E). Conversely, the CAEs treatment induced a remarkable reduction in the relative abundance of Gammaproteobacteria. The heatmap analysis on bacterial community delineated the relative abundance of microbial families in Figure 7F. At the species level, CAEs treatment increased the relative abundance of beneficial species, such as *Blautia hansenii*, *Pseudoflavonifractor capillosus*, and *Alloprevotella rava*, which were depleted in the DSS‐induced group (Figure 7G). Moreover, the upregulated relative abundance of potential pathogens such as *Salmonella enterica*, *Klebsiella pneumoniae*, and *Streptococcus oralis*, was found in the DSS‐induced group. These species were decreased after oral administration of CAEs (Figure 7H). Both DSS and CAEs treatments increased the relative abundance of beneficial bacteria, such as *Romboutsia sedimentorum*, *Flavonifractor plautii*, and *Parabacteroides merdae* (Figure 7I). Overall, CAEs effectively bolster the abundance of beneficial bacteria, mitigate pathogenic populations, and enhance the richness and diversity of gut microbiome in DSS‐induced colitis‐bearing mice. This modulation of gut microbiota by CAEs might play a role in augmenting the therapeutic efficacy against colitis.

### 2.8 CAEs Attenuate DSS‐Induced Colitis by Targeting the Gut Microbiota

**Figure 8:**
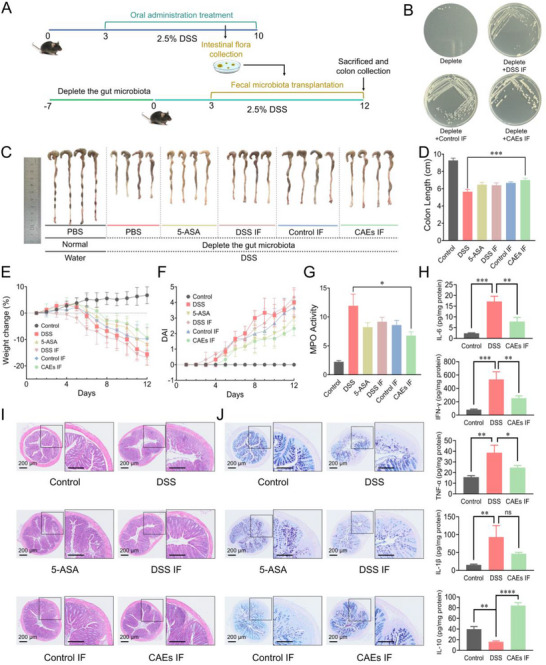
Therapeutic efficacy assessment of fresh fecal microbiota transplantation from donor mice in a DSS‐induced colitis pseudo‐germ‐free (PGF) mouse model. A) Schematic illustration of drug intervention and fecal microbiota transplantation in an acute DSS‐induced PGF mouse model. B) Microbiological plating of fecal homogenates on agar Petri dishes validating successful establishment of PGF model and assessing the efficacy of fecal microbiota transplantation. C) Gross morphological assessment of excised colons. D) Quantification of colon lengths presented as average measurements. E) Mouse body weight. F) Disease activity index (DAI) scores assessment. G) Myeloperoxidase (MPO) activity in colonic tissues. H) Quantitative assessment of cytokine levels, including IL‐6, IFN‐γ, TNF‐α, IL‐1β, and IL‐10 in colon tissue homogenates. I) Hematoxylin‐eosin (H&E) staining of histological sections illustrating tissue morphology and the extent of architectural distortion, and cellular infiltrates. J) AB/PAS staining of histological sections illustrating the integrity of mucus layer and goblet cell population. Data were presented as mean ± SD, with *n* ≥ 3. Statistical significance was denoted by asterisks, ^*^ *p *< 0.05, ^**^ *p* < 0.01, ^***^ *p* < 0.001, and ^****^ *p* < 0.0001, no statistical significance was indicated by ns.

Mounting evidence demonstrates that gut microbiomes are closely involved in the pathogenesis of UC and fecal bacteria transplantation is a strategy for UC therapy.^[^ ^22^ ^]^ Considering this, the potential of CAEs treatment to modulate gut microbiota composition was investigated. We induced colitis in pseudo‐germ‐free (PGF) mice to assess the efficacy of CAEs in colitis (**Figure** **8**A). After establishing the PGF mouse model and depleting the gut microbiota, fecal material from colitis mice of the control, DSS and CAEs groups were transplanted into PGF mice with colitis (Figure 8B). CAEs IF (intestinal flora) treatment restored the colonic lengths (Figure 8C,D), increased the body weight of the mice (Figure 8E), and decreased DAI index (Figure 8F), as well as a reduction in MPO activity (Figure 8G) of the colitis mice. As shown in Figure 8H, in DSS treatment, the levels of the pro‐inflammatory cytokines, including IL‐6, IFN‐γ, TNF‐α, and IL‐1β were significantly upregulated, while the anti‐inflammatory cytokine IL‐10 was dramatically down‐regulated compared to that of Negative Control group. The CAEs IF reversed this inflammatory‐response phenotype. Furthermore, the CAEs IF treatment showed improved gross symptoms of colitis, alleviated colonic inflammation, edema, and mucosal injury, and a restoration of crypt architecture in the recipient mice, when compared with the DSS IF treatment (Figure 8I,J). These findings suggest that gut microbiota plays an indispensable role in mediating the therapeutic effects of CAEs in colitis. Accordingly, an investigation was conducted to assess the capacity of some gut microbiota in the treatment of UC. Specifically, *Blautia hansenii, Pseudoflavonifractor capillosus*, *Alloprevotella rava*, *Romboutsia sedimentorum*, *Flavonifractor plautii*, and *Parabacteroides merdae* were identified as having a potential therapeutic value in UC, as illustrated in Figure S19 (Supporting Information). Moreover, CAEs significantly inhibited the in vitro growth of pathogenic bacteria, including *Salmonella enterica* and *Klebsiella pneumonia*. However, CAEs did not affect the growth of *Blautia hansenii* in vitro (Figure S20, Supporting Information).

### 2.9 The Stability and Biosafety Evaluation of CAEs

**Figure 9:**
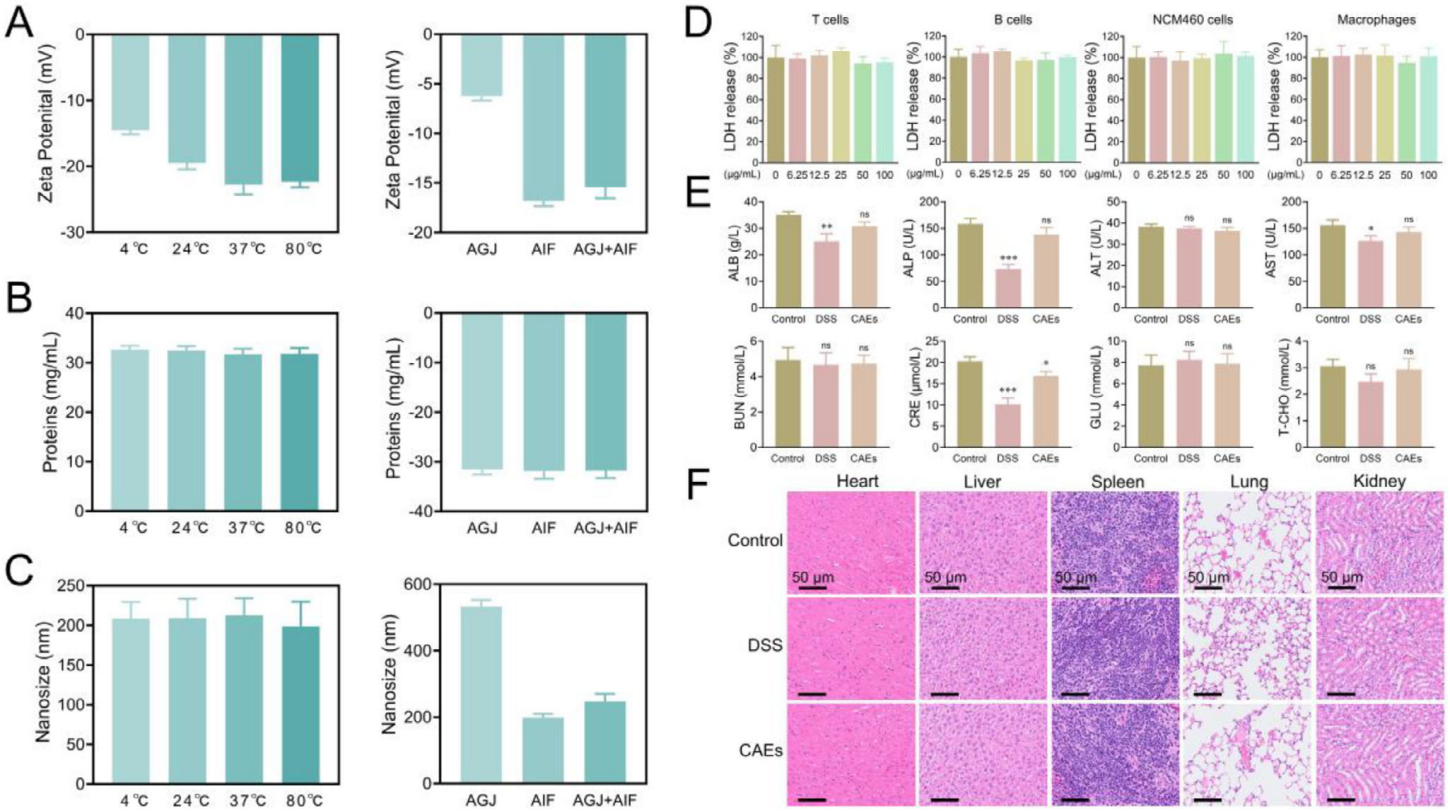
Assessment of the stability and safety profiles of Centella Asiatica‐derived exosomes (CAEs). A) Analysis of zeta potential for CAEs across a range of temperatures, as well as in artificial gastric juice (AGJ) and artificial intestinal fluid (AIF). B) Quantitative determination of protein concentrations within CAEs under varying conditions. C) Characterization of the nanoscale size of CAEs under different conditions. D) Evaluation of the cytotoxic effects of CAEs on primary T and B lymphocytes, NCM460, and primary macrophages, utilizing LDH assay. E) Blood biochemistry profiling to assess the systemic safety of CAEs. F) Histopathological examination via H&E staining of major organs (heart, liver, spleen, lung, and kidney). Data were presented as mean ± SD, with *n* ≥ 3. Statistical significance was denoted by asterisks, ^*^ *p *< 0.05, ^**^ *p* < 0.01, and ^***^ *p* < 0.001, no statistical significance was indicated by ns.

The zeta potential of the CAE nanomaterials was systematically evaluated under various temperature conditions, including 4 °C, 24 °C, 37 °C, and 80 °C, as well as in distinct simulated digestive milieus: artificial gastric juice (AGJ), artificial intestinal fluid (AIF), and a composite of both AGJ and AIF (AGJ + AIF). As delineated in **Figure** **9**A, the zeta potential of CAEs exhibited a relatively stable profile across the temperature gradient, oscillating between approximately −14.0 and −24.4 mV. Conversely, a pronounced decline in zeta potential was detected upon exposure to the digestive fluids, with AGJ eliciting the most substantial reduction to around −6.2 mV. In contrast, AIF and AGJ + AIF yielded marginally elevated values, approximating −15 mV. Figure 9B shows that temperature variations exerted a negligible influence on the protein concentration, which was consistently maintained at approximately 32 mg mL^−1^ across the entire temperature range. However, immersion in the digestive fluids did not induce any significant perturbations in protein concentration, implying that this nanomaterial remains stable in both AGJ and AIF settings. Figure 9C portrays that CAEs maintained a consistent nanoscale dimension across the temperature spectrum, with dimensions remaining proximate to 205 nm. Interestingly, a marked aggregation of CAEs was observed upon interaction with AGJ in particle size reached 532 nm. In contrast, within AIF, CAEs were well‐dispersed with their size reducing to 198 nm. This decrement in size suggests a potential aggregation in the nanomaterials within the AIF context, potentially attributable to interactions with bile salts or other constituents of the intestinal milieu. Moreover, the particle size and zeta potential of CAEs were evaluated under different storage conditions, as well as their gastrointestinal stability (Figure S21, Supporting Information). Those data demonstrated that CAEs remain highly stable during storage at −80 °C for over 180 days.

Biosafety is a fundamental prerequisite for the clinical translation of therapeutic agents. To assess the biosafety of CAEs, an in vitro cytotoxicity evaluation was conducted on T cells, B cells, NCM460 cells, and macrophages. The cell viability data showed that CAEs exhibited no significant cytotoxic effects on these four cell lines at concentrations up to 100 µg mL^−1^ (Figure 9D). Subsequently, the in vivo biosafety of CAEs was examined by oral administration in DSS‐induced colitis mice. Figure 9E indicates that there were minimal fluctuations in activity levels of indicator enzymes among the CAEs‐treated UC mice, including ALB, hepatotoxicity markers (ALP, ALT, and AST), nephrotoxicity indicators (BUN and CRE), blood glucose (GLU) and blood fat (T‐CHO), when compared to that of Negative Control group. Furthermore, the histological examination of the main organs (heart, liver, spleen, lung, and kidney) following H&E staining showed no overt abnormal lesions in the internal organs of the CAEs treatment group relative to the Negative Control group (Figure 9F). These findings from both in vitro and in vivo assessments demonstrate that orally administered CAEs possess excellent safety profiles, indicating their potential as a viable therapeutic intervention with minimal adverse effects.

## 3 Discussion

In this study, our findings underscore the profound therapeutic potential of miRNAs derived from plant‐based exosomes, specifically those from Centella Asiatica, in the treatment of UC. The detailed physicochemical characterization, molecular profiling, and in vivo efficacy studies of CAEs have elucidated their multidimensional capacity to modulate immune responses and influence gut microbiota dynamics, ultimately contributing to their therapeutic effects in a murine model of UC.

One of the most intriguing findings of this study is the elucidation of miRNAs as pivotal bioactive molecules in the plant‐derived exosomes. Plant‐derived exosomes are adept at transferring miRNAs, and these miRNA‐enriched plant exosomes have been shown possess the capacity to modulate the activity of immune cells within the biological system.^[^ ^23^ ^]^ This discovery underscores the potential of Centella Asiatica‐derived miRNAs as regulators of immune cell function, highlighting their significance in the field of immunology and related diseases. The miRNA profiling of CAEs has identified the presence of specific miRNAs, such as *aof‐miR159*, *fve‐miR396c‐3p*, and *aof‐miR396b*, which are recognized for their capacity to post‐transcriptionally modulate gene expression. Our data demonstrate that the fragments of *fve‐miR396c‐3p* and *aof‐miR396b* exhibit the potential sequence match with those genes of *Peak1, Gphn*, and *Rpl36*. Peak1, a structurally‐defined pseudokinase enriched in membrane protrusions, that regulate cytoskeletal dynamics and motility.^[^ ^24^ ^]^ Gphn, beyond its neuronal role of scaffolding inhibitory receptors, is essential for molybdenum cofactor biosynthesis.^[^ ^25^ ^]^ *Rpl36*, a core 60S subunit component, is critical for global protein synthesis. Their association with UC requires further experimental evidence for elucidation. These miRNAs have been shown to interact with immune‐related genes, thereby influencing the expression of those genes and consequently modifying the functionality of immune cells in UC. This modulation is also supported by transcriptome analysis, which elucidates the roles of these miRNAs in the context of UC pathogenesis. The therapeutic efficacy of these miRNAs is postulated to stem from their capacity to attenuate the inflammatory response by engaging key signaling cascades in immune cells. Meanwhile, single‐cell transcriptome analysis unveiled a pronounced alteration in the gene expression profiles of immune cells, characterized by an upregulation of genes implicated in immune activation and a down‐regulation of those associated with inflammation. This finding suggests that the miRNAs from CAEs play a crucial role in orchestrating the immune response, potentially by enhancing anti‐inflammatory pathways and dampening pro‐inflammatory signals. This finding aligns with emerging research that emphasizes the role of miRNAs in immune modulation across various biological systems.^[^ ^23b^ ^]^

Exosomes are recognized as natural nanocarriers with a highly efficient mechanism for targeted delivery of bioactive molecules, including miRNAs, to specific cells. Cellular uptake studies using CAEs labeled with fluorescent PKH 26 dye have demonstrated robust internalization of CAEs by both NCM460 and RAW 264.7 cells, thereby confirming the efficacy of CAEs in delivering their cargo to both immune and colonic epithelial cells. The sustained fluorescence signal observed in these cells indicates a stable and enduring release profiles of miRNAs, which may underpin the therapeutic effects observed in vivo. Furthermore, the in vivo distribution studies highlighted the selective accumulation of CAEs within the inflamed regions of the colon, a pivotal attribute for their targeted therapeutic intervention. This targeted delivery capability is crucial for amplifying the therapeutic efficacy with reducing systemic side effects. The ability of plant‐derived exosomes to selectively home to sites of inflammation and deliver their miRNA cargo with precision positions them as a promising vector for miRNA‐based therapeutics. This approach holds significant promise for developing personalized medicine strategies, particularly in the context of IBD such as UC, where localized treatment is paramount.

CAEs regulate metabolism in DSS‐induced model mice, manifested as the metabolic profile shifting toward the normal control group and the concentration recovery of key serum metabolites (e.g., l‐Carnitine, indoleacetaldehyde, methyl 3‐indolylacetate, indolelactic acid, lysophosphatidylcholine, glutamic acid, tetrahydrocorticosterone, phenyllactic acid, 2‐hydroxy‐3‐methylbutyric acid, and 4‐methylphenol) and thus providing metabolomic evidence for revealing the mechanism of action of CAEs and screening biomarkers for efficacy evaluation. l‐Carnitine, a quaternary ammonium compound essential for mitochondrial fatty acid β‐oxidation, is dysregulated in UC/IBD, reflecting impaired energy metabolism in intestinal tissues.^[^ ^26^ ^]^ l‐Carnitine supplementation might demonstrate anti‐inflammatory effects through enhanced mitochondrial function and reduced oxidative stress in experimental colitis. Tryptophan‐derived metabolites, including indoleacetaldehyde, methyl 3‐indolylacetate, and indolelactic acid, might function as critical immunomodulatory mediators through aryl hydrocarbon receptor (AhR) activation.^[^ ^27^ ^]^ Tryptophan deficiency often contributes to the development of IBD, or aggravates disease severity.^[^ ^28^ ^]^ The elevation of lysophosphatidylcholine in the digestive system is an important biomarker, serving as a pro‐inflammatory mediator and potential therapeutic target.^[^ ^29^ ^]^ Conversely, glutamic acid depletion reflects increased metabolic demand during inflammation, supporting glutamine supplementation as a barrier‐protective intervention. However, its clinical benefits require further investigation through translational research.^[^ ^30^ ^]^ Phenyllactic acid deficiency indicates loss of beneficial microbial metabolic capacity, while elevated 2‐hydroxy‐3‐methylbutyric acid signifies increased protein catabolism and metabolic dysfunction.^[^ ^31^ ^]^ 4‐Methylphenol accumulation serves as a dysbiosis biomarker, correlating with pathogenic bacterial overgrowth and disease activity.^[^ ^32^ ^]^ Collectively, these metabolites provide a metabolic signature of IBD, and the combination panels indicate the potential for disease monitoring and treatment response assessment, also demonstrating the interconnection among microbial dysbiosis, metabolic dysfunction, and intestinal inflammation.

Gut microbiomes are a critical determinant in the etiology and progression of UC, making its modulation a significant target for therapeutic intervention. The observed increase in beneficial bacteria such as *Blautia hansenii* and the concurrent decrease in pathogenic species such as *Klebsiella pneumoniae* suggest that CAEs have the potential to restore gut microbial balance, which is frequently perturbed in UC patients.^[^ ^33^ ^]^ Gut microbiota not only contributes to the maintenance of intestinal homeostasis but also influences immune responses. The LEfSe analysis provided insights into the dynamics of gut microbial communities, revealing that CAEs treatment enriched the relative abundance of certain bacterial taxa that are associated with anti‐inflammatory properties. This microbiota modulation could potentially synergize with the miRNA‐mediated regulation of immune cells, thereby amplifying the therapeutic outcomes.

Taken together, CAEs represent a promising therapeutic strategy for UC through the multifaceted and interconnected regulation of host physiology, gut microbiota, and metabolite production. CAEs serve as a vehicle for miRNA cargo carried within nanovesicles directly modulates immune responses and influence gut microbiota dynamics, thereby reducing colitis severity. Concurrently, these exogenous miRNAs, alongside bioactive lipids and proteins present in the exosomal membrane, actively shape the gut microbial ecology—potentially enriching beneficial taxa while suppressing pathobionts. This targeted modulation of the microbiota, in turn, drives a profound shift in metabolome, further fortifying the epithelial barrier and dampening inflammation. Critically, the integration of these three dimensions—miRNA‐mediated host gene silencing, exosome‐induced microbial restructuring, and microbiota‐derived metabolite regulation creates a self‐reinforcing, anti‐inflammatory loop that synergistically addresses the core dysregulations of UC. In the future, standardizing bioactive profiles, defining miRNA‐metabolite‐microbe axes, and exploring drug synergies are pivotal for precision‐integrated therapy.

Translating CAEs from murine models to human clinical applications entails key challenges and significant opportunities. The foremost hurdle is scalable, cost‐effective production, requiring advanced methods for large‐scale plant cell cultivation and exosome isolation. Additionally, the unique nature of plant‐derived nanoparticles poses regulatory complexities, necessitating robust data on safety, efficacy, and composition, as well as early engagement with regulatory agencies to ensure compliance. Identifying optimal patient populations and establishing appropriate clinical protocols are also critical, particularly given the promise of CAEs in regenerative medicine and tissue repair. Despite these challenges, CAEs offer considerable potential for targeted drug delivery and tissue regeneration due to their biocompatibility and genetic payload capacity. Addressing issues of scalability, regulation, and patient selection will be essential to fully realize the clinical potential of CAEs.

Plant‐derived exosomes offer a novel, potent strategy for managing inflammatory diseases by enabling cross‐kingdom miRNA delivery. These natural nanovesicles efficiently transport functional plant miRNAs into mammalian cells. Once internalized, the miRNAs can integrate into the host RNA‐Induced Silencing Complex (RISC), modulating expression of key genes, such as those of inflammatory mediators. This leverages intrinsic plant miRNA anti‐inflammatory properties for potentially more precise targeting than conventional therapies. Crucially, plant‐derived exosomes possess inherent advantages over synthetic carriers, such as enhanced biological stability, superior tissue penetration, inherent immune evasion, and passive targeting to inflamed tissues. Overall, leveraging plant exosomal miRNAs for cross‐kingdom regulation holds significant potential as a precise and safe therapeutic strategy for managing chronic inflammatory diseases.

Lastly, but most importantly, safety is a paramount concern for therapeutic interventions. The in vitro and in vivo biosafety assessments conducted in this study substantiated that CAEs do not exhibit significant cytotoxicity or adverse effects on major organs, even at relatively high concentrations. This favorable safety profile, coupled with their natural origin and biocompatibility, renders CAEs as a compelling candidate for clinical translation. Nevertheless, the present study also exhibits certain constraints that warrant acknowledgement. The research has pinpointed *aof‐miR396b* and *fve‐miR396c‐3p* as promising candidates that need further in‐depth investigation. By the same token, these miRNAs demonstrate great potential as biomarkers or therapeutic targets, warranting comprehensive analysis of their functional roles, mechanisms of action, and clinical implications in UC and its treatment paradigms.

## 4 Conclusion

**Figure 10:**
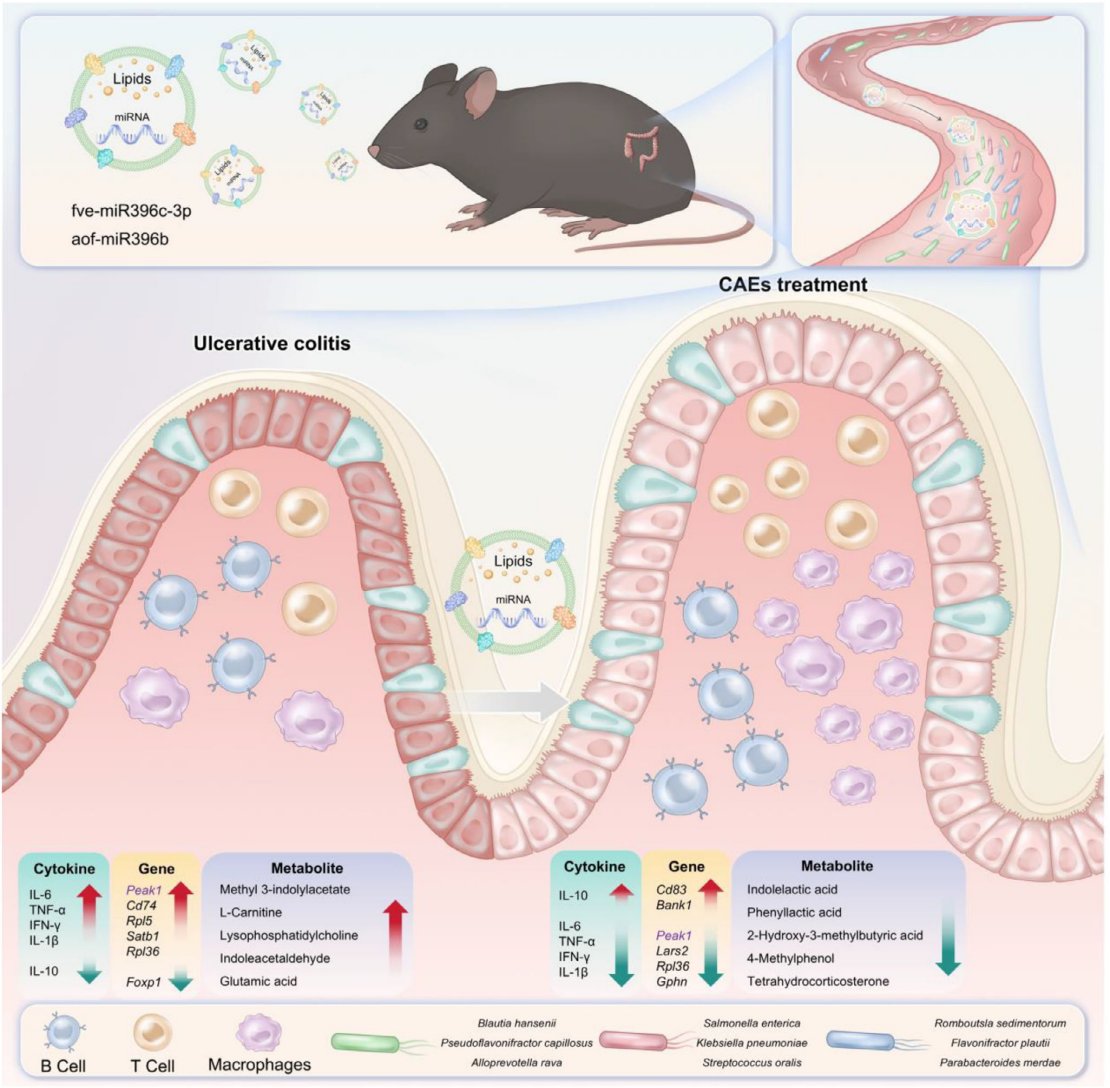
The potential mechanisms of anti‐colitis effects of Centella Asiatica‐derived exosomes (CAEs).

In conclusion, this study delineates that miRNA‐enriched plant‐derived exosomes, specifically those from Centella Asiatica, can effectively regulate immune cells and modulate gut microbiota to exert therapeutic effects for UC (**Figure** **10**). The mechanisms of action, which include targeted delivery of miRNAs, modulation of gene expression in immune cells, and alteration of gut microbiota composition, underscore the potential of CAEs as an innovative, natural therapeutic approach for IBD. The utilization of plant‐derived exosomes as carriers for miRNAs represents a promising and pioneering approach to modulate immune responses and treat inflammatory diseases such as UC, paving the way for the development of novel natural, bioactive therapeutics.

## 5 Experimental Section

### Materials

Fresh Centella Asiatica was harvested from Zhongshan or Qingyuan City, Guangdong Province, China. High‐purity sucrose was purchased from Aladdin Reagent (Shanghai, China). A comprehensive suite of cell culture reagents was procured from Gibco (NY, USA), including fetal bovine serum (FBS), Dulbecco's Modified Eagle Medium (DMEM), McCoy's 5A Medium, Phosphate buffered saline (PBS), Penicillin/Streptomycin (P/S), and 0.25% trypsin‐EDTA. DiR and PKH 26 were obtained from MedChemExpress (NJ, USA). DSS with a molecular weight range of 36–50 kDa was supplied by MP Biomedicals (CA, USA). A panel of ELISA kits for the quantification of murine cytokines, including IL‐6, IFN‐γ, TNF‐α, IL‐1β, and IL‐10, was provided by NeoBioscience Technology Co., Ltd (Shenzhen, China). 4′, 6‐diamidino‐2‐phenylindole (DAPI), sample loading buffer for sodium dodecyl sulfate‐polyacrylamide gel electrophoresis (SDS‐PAGE) was supplied by Beyotime Biotech Inc. (Taizhou, China). The F‐actin staining kit was sourced from TraKine. The Bicinchoninic Acid (BCA) Protein Assay Kit, a product of Pierce, Thermo Fisher Scientific Company (MA, USA), was used for protein quantification. B Cell Isolation Kit (Mouse, 130‐090‐862) and CD4^+^ T Cell Isolation Kit (Mouse, 130‐104‐454) were supplied by Miltenyi Biotec (Bergisch Gladbach, Germany).

### Isolation and Purification of Plant‐Derived Exosomes

Fresh Centella Asiatica was washed thoroughly with deionized ice‐water and subsequently macerated using a mechanical blender to extract the juice. The juice underwent a series of centrifugation steps, initially at 1000 *g* for 10 min, followed by 3000 *g* for 30 min, and then 10 000 *g* for 1 h to remove debris and fibers. The supernatant was then subjected to ultracentrifugation at 150 000 *g* for 90 min to concentrate the exosomal fraction at 4 °C. The pellet obtained was resuspended in PBS. For purification of exosomes, the resuspended pellet was transferred to a sucrose density gradient consisting of 8%, 30%, 45%, and 60% sucrose solutions and ultracentrifuged at 150 000 *g* for an additional 90 min. The exosomal fraction, termed CAEs, was carefully harvested from the interphase between 30% and 45% sucrose layers.

### Characterization of Plant‐Derived Exosomes

The characterization of CAEs was conducted using multiple analytical techniques. Protein concentrations of CAEs were determined using BCA Protein Assay Kits, adhering to the manufacturer's guidelines. The particle size distribution of CAEs was assessed by NTA using a ZetaVIEW instrument from PARTICLE METRIX. The surface charge of CAEs was evaluated using Malvern Zetasizer. Transmission electron microscopy (HT‐7700, Hitachi) imaging was utilized for morphological examination. CAEs were prepared by depositing 10 µL of the sample onto a copper grid, followed by staining with 1% uranyl acetate for one min. Excess stains were removed, and the grid was air‐dried to prepare for imaging. The stability of CAEs was evaluated by monitoring changes in zeta potential after storage at 4 °C, 24 °C and 37 °C for 30 min using the DLS. Protein profiling of CAEs was performed using SDS‐PAGE. Samples were prepared by mixing with SDS‐PAGE loading buffer and subjected to heat denaturation at 95 °C for 10 min. Then, equal amounts of protein (40 µg per well) were then loaded and separated on a 10% SDS‐polyacrylamide gel under a voltage of 80 V (30 min) initially, followed by 120 V (1 h). The resolved proteins were visualized by staining with Coomassie Blue, destained with deionized water, and imaged. For the long‐term stability assessment, CAEs were stored at −80 °C for a period of 90 and 180 days. Post‐storage, CAEs were allowed to equilibrate to 24 °C and their protein, particle size, and zeta potential were re‐evaluated for stability using the methods mentioned above.

### AFM Analysis

CAEs were diluted with ultrapure water and deposited onto freshly cleaved mica sheets. After air drying, the samples were imaged in tapping mode using the Ultra Speed 2 AFM system (Bruker). Surface topography and particle size were analyzed using Nanoscope Analysis software. All imaging experiments were performed at the Shenzhen Bay Laboratory (Shenzhen, China).

### Cryo‐TEM Analysis

CAEs were suspended in PBS, and 3–5 µL of the sample was loaded onto glow‐discharged carbon‐coated copper grids. Grids were blotted to remove excess liquid and rapidly vitrified in liquid ethane using a Vitrobot Mark IV. Imaging was performed using a Tundra Cryo‐TEM (Thermo Fisher Scientific) operated at 100 kV. Images were acquired using a Ceta CMOS camera, and post‐acquisition noise reduction was performed to enhance image clarity while preserving structural integrity. All imaging experiments were performed at the Shenzhen Bay Laboratory (Shenzhen, China).

### Qualitative Analysis of Phytochemicals

The qualitative analysis of phytochemicals in CAEs was performed using ultra‐high‐performance liquid chromatography coupled with quadrupole time‐of‐flight mass spectrometry (UHPLC‐Q‐TOF/MS). The sample was separated using a HSS T3 column (2.1 × 150 mm, 1.8 µm, Waters). Mobile phase A was an aqueous solution of formic acid (a ratio for formic acid to water of 1:1000 v/v), and mobile phase B consists of acetonitrile. The elution program was meticulously crafted to gradually increase the concentration of Phase B, that is 0–5 min, 5–30% B; 5–10 min, 30–41% B; 10–17 min, 41–48% B; 17–25 min, 48–95% B; 25–30 min, 95% B. The flow rate was maintained at 0.25 mL min^−1^, and the column temperature was regulated at 40 °C. The mass spectrometry parameters are finely tuned for positive (ESI^+^) and negative ion (ESI^−^) scanning modes. The heater temperature was set to 500 °C, and the sheath gas flow rate, aux gas flow rate, and sweep gas flow rate were all balanced at 50 arbitrary units. The spray voltage was adjusted to −4.5 kV, and the S‐Lens RF Level was set to 50%. The MS1 scan ranges were set from *m/z* 100 to 1300. The data acquisition and processing were conducted using PeakView 1.2 software (SCIEX).

### Quantitative Analysis of Phytochemicals

High‐performance liquid chromatography (HPLC) was performed using a Thermo UltiMate 3000 UHPLC+ system. Chromatographic separation was achieved on a Welch Ultimate AQ‐C18 column (250 × 4.6 mm, 5 µm particle size). The mobile phase consisted of solvent A (0.1% phosphoric acid in water) and solvent B (acetonitrile, HPLC grade, Thermo Fisher). The gradient elution program was as follows: 0–10 min, 5–20% B; 10–30 min, 20–30% B; 30–45 min, 30–50% B; 45–60 min, 50% B. The flow rate was maintained at 1.0 mL min^−1^, with a column temperature of 30 °C. The detection wavelength was set at 205 nm, and the injection volume was 10 µL. Ultrapure water used in the preparation of mobile phases was produced by a Milli‐Q system (Millipore).

### Proteomic Analysis

CAEs were precipitated using TCA/acetone, while plant‐derived exosomes were homogenized in SDT buffer (4% SDS, 100 × 10^−3^ m Tris‐HCl, pH 7.6) using an MP FastPrep‐24 homogenizer. After sonication and boiling, lysates were centrifuged (14 000 *g*, 40 min) and supernatants quantified via BCA assay. Protein reduction and alkylation preceded enzymatic digestion using dithiothreitol (DTT) (40 × 10^−3^ m, 37 °C, 1.5 h) and iodoacetamide (IAA) (20 × 10^−3^ m, RT, dark), respectively. Samples were subjected to Microcon units (10 kDa cutoff filter) and washed with Universal antibody dilution buffer and 25 × 10^−3^ m NH_4_HCO_3_ buffer. Trypsin digestion (1:50 w/w, 37 °C, 15–18 h) generated peptides that were desalted on C18 Cartridges (Empore SPE Cartridges MCX, 30 UM, Waters), vacuum‐concentrated, then dissolved in formic acid (0.1% v/v). Peptide concentration was determined by UV absorption at 280 nm. LC‐MS/MS analysis was performed on a Vanquish Neo UHPLC system coupled with an Orbitrap Astral mass spectrometer (Thermo Scientific) in data‐independent acquisition (DIA) mode. MS1 spectra (380–980 *m/z*) were acquired at 240 000 resolutions with 5 ms max injection time. MS2 analysis employed 299 variable windows (2 *m/z* isolation width) using high‐energy collisional dissociation (HCD) fragmentation (25 eV collision energy), with 500% AGC target and 3 ms max injection time. DIA data were processed using DIA‐NN software suite. Protein‐level quantification was normalized by median centering to minimize batch effects. The experiments were supported by Shanghai Applied Protein Technology Co., Ltd.

### Lipidomic Analysis

The liquid chromatography‐tandem mass spectrometry (LC‐MS/MS) method was applied for lipid analysis. Reverse‐phase chromatography utilized a CSH C18 column (1.7 µm, 2.1 mm  ×  100 mm, Waters). Mobile phase A was prepared with acetonitrile aqueous solution with a ratio of acetonitrile to water of 6:4 (v/v), supplemented with 0.1% formic acid and 0.1 × 10^−3^ m ammonium formate. Mobile phase B was an acetonitrile isopropanol solution with a ratio of acetonitrile to isopropanol of 1:9 (v/v), containing 0.1% formic acid and 0.1 × 10^−3^ m ammonium formate. The elution program was intricately designed to gradually increase the concentration of mobile phase B, that is 0–3.5 min, 40% B; 3.5–13 min, 40–75% B; 13–19 min, 75–99% B; 19–19.1 min, 99–40% B; 19.1–24 min, 40% B. The flow rate was maintained at 0.3 mL min^−1^, and the column temperature was regulated at 45 °C. The mass spectrometer was operated in both positive (ESI^+^) and negative ion (ESI^−^) ionization modes. The heater temperature was set at 300 °C, the sheath gas flow rate at 45 arbitrary units, the aux gas flow rate at 15 arbitrary units, and the sweep gas flow rate at 1 arbitrary unit. The spray voltage was set to 3.0 kV, and the capillary temperature was maintained at 350 °C. The S‐lens RF level was adjusted to 50%. For the MS1 scan, the scan range was set from *m/z* 200 to 1800. The identification of lipids was performed using LipidSearch software (Thermo Fisher Scientific), a widely recognized tool in the field of lipidomics. The mass tolerance for both precursor and fragment ions was set to 5 parts per million (ppm). The experiments were supported by Shanghai Applied Protein Technology Co., Ltd.

### MicroRNA Analysis

An exoRNeasy Maxi Kit (Qiagen, 77 164) was used to isolate and purify the total RNA. The quantity and purity of the extracted RNA from each sample were measured using NanoDrop ND‐1000 spectrophotometer (Wilmington, DE, USA). The RNA integrity was evaluated by Agilent 2100 Bioanalyzer, which yielded RNA integrity numbers (RIN) greater than 7.0. The TruSeq Small RNA Sample Preparation Kits (Illumina, San Diego, USA) were employed to construct miRNA sequencing libraries. Subsequently, single‐end sequencing was conducted using a NovaSeq 6000 Sequencing System (Illumina). The experiments were supported by Shanghai Applied Protein Technology Co., Ltd.

### Animals and Cell Lines

Male C57BL/6J mice (age range 6–8 weeks) were procured from Zhuhai Bestest Biotechnology Co., Ltd. All animal studies were performed following the National Institutes of Health (NIH) Guidelines for the Care and Use of Laboratory Animals, and the animal experimental protocols were approved by the Animal Ethics Committee of Zunyi Medical University's Zhuhai Campus (ZHSC‐2‐[2024]001). For in vitro studies, murine macrophage RAW 264.7, human NCM460, and human HT‐29 cell lines were obtained from the American Type Culture Collection (ATCC). RAW 264.7 and NCM460 cells were cultured in DMEM, and HT‐29 cells were cultured in McCoy's 5A medium. All media were supplemented with 10% (v/v) FBS and 100 IU mL^−1^ P/S. All cell cultures were maintained in a humidified atmosphere containing 5% CO_2_ at 37 °C.

### Cellular Uptake and Retention

The assessment of cellular uptake and retention of CAEs (5 µg mL^−1^) labeled with PKH 26 was conducted employing advanced imaging and flow cytometry in NCM460 and RAW 264.7 cells. Both cell types were plated at a seeding density of 2 × 10^5^ cells per well in confocal dishes. The cells were then treated with PKH 26‐CAEs and counterstained with DAPI for nucleus labeling and an F‐actin Staining Kit to visualize the cytoskeletal actin filaments. The colocalization and internalization of CAEs within cells were examined using an OLYMPUS IX83 inverted microscope. To quantify cellular uptake over time, NCM460 and RAW 264.7 cells were plated at the same seeding density in a 24‐well plate and exposed to PKH 26‐CAEs for 1, 2, 4, and 6 h. Subsequently, the cells were either enzymatically digested or mechanically scraped off to prepare single‐cell suspensions, followed by washing with PBS for 3 times at 4 °C. The cellular fluorescence was then measured using a flow cytometer (CytoFLEX, Beckman Coulter). After incubation with fresh blank DMEM, the retention of PKH 26‐labeled CAEs (PKH 26‐CAEs) by RAW 264.7 and NCM460 cells was monitored over an extended period. The cells were then imaged at 12‐, 24‐ and 48‐h post‐treatment to track the intracellular retention trafficking of the labeled exosomes.

### Endocytosis Mechanism of Plant‐Derived Exosomes

Endocytosis mechanism of CAEs was evaluated following previous report.^[^ ^34^ ^]^ RAW 264.7 cells were polarized to the M1 phenotype by incubation with 200 µg mL^−1^ LPS. M1 macrophage and NCM460 cells were pretreated for 1 h with inhibitors including M‐β‐CD (5 × 10^−6^ m), Filipin (5 µg mL^−1^), chlorpromazine (10 µg mL^−1^), and AMH (3 × 10^−3^ m). Subsequently, PKH 26‐labeled CAEs (10 µg mL^−1^) were added to the cells for co‐incubation. Flow cytometry analysis was performed to assess cellular uptake. Negative control was established using unstained cells with DAPI and FITC to account for autofluorescence. Additionally, cell images were captured using an OLYMPUS IX83 inverted fluorescence microscope.

### Biodistribution of Plant‐Derived Exosomes

Oral administration of DiR‐CAEs was carried out in mice with DSS‐induced acute colitis, and the distribution of CAEs was monitored at 0‐, 1‐, 2‐, 4‐, 6‐, 12‐, and 24‐h intervals using IVIS imaging system (Caliper PerkinElmer). According to the DiR staining protocol, CAEs were labeled after quantification and then administered orally to mice at a dose of 10 mg kg^−1^. Following the in vivo imaging, the animals were sacrificed, and their colons were excised for ex vivo imaging to ascertain the accumulation of CAEs at the site of inflammation. To further investigate the therapeutic potential of CAEs, mice that had undergone drug treatment were also administered DiR‐CAEs. The fluorescence intensity within the colonic tissues was assessed to monitor the distribution and retention of CAEs.

### In Vivo Distribution of Exosomal Proteins

To assess the tissue distribution of orally administered CAEs, mice were given a single dose of 10 mg kg^−1^ CAEs via oral gavage. At 6 h post‐administration, the mice were euthanized, and major organs (heart, liver, spleen, lung, kidney, stomach, small intestine, and colon) along with blood samples were collected. Tissues were homogenized in PBS and centrifuged to obtain clear supernatants. The concentration of CAEs in each sample was determined using an ELISA kit specific to the plant‐derived protein ribulose‐1,5‐bisphosphate carboxylase/oxygenase large subunit (RbcL), according to the manufacturer's protocol. Absorbance was measured at 450 nm using a microplate reader, and RbcL concentrations were quantified based on a standard curve.

### Nitric Oxide (NO) Level Determination

The quantification of NO release was assessed using Griess reagent system. RAW 264.7 cells were seeded in 24‐well plates (2  ×  10^5^ cells per well) and allowed to adhere overnight. Following this, the cells were treated with varying conditions, including CAEs, CAC, CAEsC, and CAEsP in the presence of LPS at a concentration of 200 ng mL^−1^ for 24 h. Then equal volumes of cell culture supernatant were mixed with Griess reagent and incubated for 15 min. The absorbance was detected at 540 nm using a microplate reader.

### Lactate Dehydrogenase (LDH) Release Determination

Cells were seeded in 96‐well plate (1 × 10^4^ cells per well) and allowed to adhere for a period of 24 h. Subsequently, the cells were preincubated with varying concentrations of CAEs, ranging from 0 to 10 µg mL^−1^, for a duration of 24 h. The quantification of LDH release into the supernatant was performed using the LDH Cytotoxicity Detection Kit (Thermo Fisher Scientific Inc., USA) according to the protocol outlined by the manufacturer.

### In Vivo Efficacy Evaluation

C57BL/6J mice (age range 6–8 weeks) were housed and acclimatized for 7 days. Mice were given 2.5% DSS dissolved in the drinking water for 10 days. Afterward, 10 mg kg^−1^ day^−1^ CAEs, 10 mg kg^−1^ day^−1^ CAC, 100 mg kg^−1^ day^−1^ 5‐ASA, 450 mg kg^−1^ day^−1^ sulfasalazine (SAS), or PBS were orally administered to mice daily. Concurrently, the control group received normal drinking water. The colitis‐associated symptoms, such as stool consistency, body weight changes, and the presence of fecal occult blood, were monitored and documented daily during the experimental period. On the 10^th^ day, mice were euthanized, and the entire colon was excised for analysis.

### Colonic MPO Activity Assay

The excised colons were washed with cold PBS and homogenized in a cold potassium phosphate buffer solution (50 × 10^−3^ m) supplemented with hexadecyltrimethylammonium bromide (0.5%, v/v). The supernatant was assessed for MPO activity by adding o‐dianisidine dihydrochloride in the presence of H_2_O_2_. The colorimetric change was monitored by recording the absorbance at 450 nm over a 5‐min period using a spectrophotometer.

### Histological Staining

The colon, spleen, liver, lung, kidney, and heart were fixed with paraformaldehyde (4% in PBS), dehydrated, and embedded in paraffin wax. Subsequently, sections were subjected to H&E staining. For combined detection of acidic mucins and glycogen, selected sections were further processed with AB‐PAS staining, using Alcian Blue (pH 2.5) prior to the PAS reaction. The stained sections were examined and imaged using microscopy (DS‐RI2, Nikon).

### Colonic Microbiome Analysis

The fresh fecal samples were collected from all the experimental mice, and genomic DNA was extracted using E.Z.N.A. soil DNA Kit. The extracted DNA was then separated with an agarose gel, and the DNA concentration and purity were measured using NanoDrop 2000 UV‐vis spectrophotometer. The hypervariable V3‐V4 region of the bacterial 16S rRNA gene was amplified using the primer pairs 338F and 806R on an ABI GeneAmp 9700 PCR thermos cycler. The amplified products were sequenced using Illumina MiSeq platform with PE300 chemistry. Raw fastq files were demultiplexed and quality‐filtered using QIIME (version 1.17). The 16S rRNA gene sequences were analyzed through the REALGENE analysis platform. Shanghai Applied Protein Technology Co., Ltd, supported the experiments.

### Nontargeted Metabolomic Analysis

Plasma samples were collected from Control, DSS, and CAEs groups, with each group comprising N ≥ 3 biological replicates. Aliquots of 200 µL from each murine plasma sample were mixed with 500 µL of methanol containing butylated hydroxytoluene (BHT, 2.3%, w/v) and formate (1%, v/v), followed by centrifugation at 14 000 *g* for 10 min. The supernatant was then washed with solution A (5% BHT reagent) and solution B (1% formate in solution A, v/v). The samples were subsequently eluted with 1 mL of methanol and dried under a stream of nitrogen gas. In metabolomics analysis, samples were re‐dissolved with 80 µL of methanol/acetonitrile mixture (1:1, v/v) and subjected to UHPLC‐Q‐TOF/MS. The separation was performed using an ACQUITY UHPLC BEH Amide column (2.1 × 100 mm, 1.7 µm) with a mobile phase consisting of 25 × 10^−3^ m ammonium hydroxide and 25 × 10^−3^ m ammonium acetate in water (mobile phase A), and acetonitrile (mobile phase B). The elution program was as follows: 0–1 min, 95% B; 14 min, 65% B; 16–18 min, 40% B; 23 min, 95% B. The flow rate was set at 0.3 mL min^−1^, and the column temperature was 25 °C. Mass spectrometry data were acquired in both positive (ESI^+^) and negative (ESI^−^) ion modes, with a heater temperature of 600 °C and an accumulation time of 0.20 s/spectra for TOF MS scan. Automatic MS/MS acquisition was performed with an *m/z* range of 50–1000 Da, an accumulation time of 0.05 s/spectra, collision energy set at 35 ± 15 eV, and a decluttering potential of 60 V for both positive and negative modes. Isotope interferences were analyzed via an exclusion window of 4 Da and 6 precursor ions per cycle. Metabolite identification was analyzed by converting raw MS data to mzXML files, peak matching, retention time alignment and peak area extraction via XCMS software. The experiments were supported by BGI Health (HK) Company Limited.

### Single‐Cell Transcriptome

The colonic tissues were harvested from Control, DSS, and CAEs groups, with triplicate samples collected for each group. Cell viability rate was above 80% after quality check and counting single‐cell suspension. Then the cells were washed and resuspended to achieve a standardized concentration of 700–1200 cells per microliter, in preparation for single‐cell sequencing using the Genomics Chromium platform (10 × Genomics). Single‐cell encapsulation into Gel Bead in Emulsion (GEMs) was formed to ensure precise cell isolation, and the GEMs were collected for reverse transcription within a PCR apparatus for cDNA synthesis and labeling. After the oil phase treatment, the amplified cDNA was purified using magnetic beads for the subsequent amplification and quality inspection. A high‐quality cDNA library was constructed and underwent a series of processes, including fragmentation, adaptor ligation, and sample index PCR. The final library pool was sequenced on the Illumina NovaSeq 6000 platform using 150‐basepair paired‐end reads. FastQC software was used to perform quality control analysis, including calculating quality scores for each read and assessing the distribution of nucleotide content. The raw Fastq data underwent cell identification and data quality statistics using Cell Ranger software. The read2 sequences were aligned to the reference genome from the Ensembl database (https://asia.ensembl.org/index.html). For cell filtration, the Seurat package was employed to filter out outlier cells based on criteria such as the number of genes and unique molecular identifiers (UMIs) expressed, mitochondrial gene expression levels, and ribosomal gene expression profiles. Cluster analysis was performed using the Seurat package, beginning with the PCA for dimensionality reduction to reduce computational load and noise. Subsequently, the Leiden clustering algorithm is applied to identify communities within the reduced dimensional space. Nonlinear dimensionality reduction methods, such as TSNE and UMAP, were utilized for visualizing the clustering patterns of the single‐cell populations. In the context of multisample integration analysis, Seurat calculated difference vectors to correct gene expression values of anchor cells, thereby mitigating technical biases and integrating multiple single‐cell datasets. Protein–protein interaction networks for the target gene sets were elucidated using STRING database (https://www.string‐db.org/), providing insights into the functional associations between proteins corresponding to the genes of interest. Cell communication analysis was performed according to the annotated expression matrix and cell types, utilizing the CellPhoneDB platform to explore gene1‐gene2 interaction networks. The experiments were supported by Shanghai Applied Protein Technology Co., Ltd.

### RNA Sequencing Analysis

Total RNA was extracted from animal tissues or cultured cells using TRIzol reagent or other appropriate extraction methods, depending on sample type. After homogenization and phase separation, the RNA‐containing aqueous phase was recovered, precipitated with alcohol, washed, and finally dissolved in RNase‐free water. RNA purity, concentration, and integrity were assessed using spectrophotometry and capillary electrophoresis to ensure suitability for downstream applications. Either ribosomal RNA was depleted, or mRNA was enriched using Oligo(dT) magnetic beads. The enriched RNA was then fragmented and reverse‐transcribed into cDNA. Library construction involved second‐strand synthesis, end repair, A‐tailing, adapter ligation, size selection, and PCR amplification. The experiments were supported by Shanghai Applied Protein Technology Co., Ltd.

### MicroRNA Binding and Molecular Docking

The mature miRNA sequences were collected from miRBase database, and the 3′ untranslated region (3′ UTR) sequences of the target genes were obtained from the National Center for Biotechnology Information (NCBI) database. Sequence alignment was performed using the miRanda algorithm, considering the complementary base pairing between the miRNA seed region and the target gene sequences. Molecular docking was performed using Autodock Vina 1.1.2 software to predict the binding affinity of the selected metabolites and the target proteins. The three‐dimensional structures of the ligands were prepared using Chem3D, and energy minimization was carried out using the MM2 force field. The target protein structures were retrieved from the Protein Data Bank (PDB). Before docking, water molecules were removed, hydrogen atoms were added, and Kollman charges were assigned. A grid box was defined to encompass the entire active site, and docking simulations were conducted with the Lamarckian Genetic Algorithm (LGA) to predict the most favorable binding conformations and affinities.

### MicroRNA Target Identification

The mRNA sequences of the genes were collected from the NCBI database. The sequences of miRNAs were paired according to A (adenine) to U (uracil), C (cytosine) to G (guanosine) to find the fragment with the highest number of pairs of miRNAs and mRNAs. The database (*rnamtom.net*) was established by our group.

### Primary Cell Isolation and qPCR

B cells and CD4^+^ T cells were isolated from mouse splenocytes using magnetic‐activated cell sorting kits (Miltenyi Biotec). Meanwhile, spleen‐derived macrophage‐like cells were obtained by plastic adherence for 2 h, followed by washing to remove nonadherent cells. All cell types were treated with 5 µg mL^−1^ of CAEs for 24 h. After treatment, total RNA was extracted, and subsequent cDNA synthesis and qPCR analysis of target gene expression were performed, with β‐actin serving as the internal control. The primers used in this experiment are listed in Table S5 (Supporting Information).

### Fecal Microbiota Transplantation

The PGF murine model was established by administering an antibiotic cocktail, comprising vancomycin (0.5 g L^−1^), ampicillin (1 g L^−1^), metronidazole (1 g L^−1^) and neomycin sulfate (1 g L^−1^). This regimen was initiated 7 days before the DSS treatment and continued throughout the experimental period to deplete gut microbiota. Concurrently, mice were randomly assigned into three groups, one group serving as normal mice and the other two groups representing DSS‐induced acute colitis mice received oral gavage of either PBS or CAEs (10 mg kg^−1^ day^−1^). Fecal samples were systematically collected from three groups. Subsequently, the PGF mice were subjected to a 2.5% DSS for 12 days. Concurrently, these mice received daily fecal microbiota transplantations from either control (Control IF), PBS (DSS IF), or CAEs (CAEs IF) donor mice. Additionally, three groups were included, wherein 100 mg kg^−1^ 5‐ASA or PBS was administered orally to mice every day, while the Control was provided with normal drinking water. The fecal material collected from randomly selected donor mice was pooled, diluted at a ratio of 1:10 (w/v) using saline, and homogenized for 1 min with a vortex. The homogenate was centrifuged at 500 *g* for 3 min to produce a clear liquid supernatant. Each recipient mouse received 200 µL of supernatant via oral gavage.

### In Vitro Evaluation of Gut Microbiota Growth

The effects of CAEs on the in vitro growth of gut microbiota were evaluated by monitoring optical density at 600 nm (OD_600_) over a 24‐h period using an automated microplate reader (BioTek Synergy H1). Overnight cultures of each bacterium were diluted in fresh Nutrient Broth (NB) to an initial OD_600_ of approximately 0.05 and aliquoted (200 µL) into the wells of 96‐well microplates. CAEs were added to achieve final concentrations of 0, 0.01, 0.1, and 1 mg mL^−1^. The plates were incubated at 37 °C with continuous orbital shaking (180 rpm) to ensure aeration. OD_600_ was measured Hly over a 20–24‐h period, with each reading blank‐corrected using sterile medium. Microbiota cultured to the endpoint were diluted (10^−6^) and plated on solid agar.

### Statistical Analysis

Data analysis was conducted using GraphPad Prism software (V.8.3.0), with the statistical significance between two comparative groups assessed via Student's t‐test. One‐way analysis of variance (ANOVA) was applied for multiple group comparisons. Results are presented as the mean ± standard deviation (SD) from multiple experiments. Statistical significance was denoted as follows, ^*^ *p* < 0.05, ^**^ *p* < 0.01, ^***^ *p* < 0.001, and ^****^ *p* < 0.0001, and ns means insignificant.

## Conflict of Interest

The authors declare no conflict of interest.

## Author Contributions

R.S. and W.T. contributed equally to this work. R.S. performed conceptualization, investigation, and data curation, and wrote the original draft. W.T. performed supervision, investigation, data curation, and reviewed and edited the writing. H.J. performed investigation and data curation. S.I.C. performed investigation and data curation. W.L. performed investigation and data curation. S.S.L. performed investigation. G.C. performed methodology and resources. Y.W. performed supervision and funding acquisition. D.H.Y. performed supervision, data curation, visualization, and reviewed and edited the writing. Z.Z. performed conceptualization, project administration, funding acquisition, and reviewed and edited the writing. All authors have read and approved the final manuscript.

## Supporting information



Supporting Information

## Data Availability

The data that support the findings of this study are available from the corresponding author upon reasonable request.
