# Supplementary material for: MicroRNA‐Enriched Plant‐Derived Exosomes Alleviate Colitis by Modulating Systemic Immunity, Metabolic Homeostasis, and Gut Microbiota
Source: Adv Sci (Weinh). 2025 Sep 24;12(42):e05921. doi: 10.1002/advs.202505921 (PMC12622485; doi:10.1002/advs.202505921)
Supplement: Supplementary file 1 — Supporting Information [file ADVS-12-e05921-s001.docx]

**Supplementary Information**

**MicroRNA-enriched Plant-derived Exosomes Alleviates Colitis by Modulating Systemic Immunity, Metabolic Homeostasis, and Gut Microbiota**

Ruipeng SHI *et al.*

*Corresponding authors.

Zhangfeng ZHONG, Email: zfzhong@um.edu.mo

Donghua YANG, Email: dong-hua.yangc@nyctcm.edu

**This file includes:**

1. Figs. S1 to S21
2. Tables S1 to S5
3. Video S1

Figure S1





Fig. S1. Extraction and purification of Centella Asiatica-derived exosomes (CAEs).

Representative images of sucrose gradient centrifugation illustrating the fraction of CAEs.

Figure S2

**

**

**Fig. S2. Chemical analysis of phytochemicals in** **Centella Asiatica-derived exosomes (CAEs).**

A) Representative total ion current (TIC) chromatogram of the CAEs sample.

B) Mass spectrometric analysis of the CAEs sample in positive ion mode.

C) Mass spectrometric analysis of the CAEs sample in negative ion mode.

Figure S3





**Fig. S3. Proteomic and miRNA profiling analysis in Centella Asiatica-derived exosomes (CAEs).**

A) Frequency analysis of nucleotide base composition (Adenine [A], Uracil [U], Cytosine [C], Guanine [G]) across various positions in miRNA sequences.

B) Consistency of retention times for iRT (indexed retention time) peptides across multiple analytical runs.

C) Enumeration of peptides identified in the CAEs from proteomic study.

D) Enumeration of proteins identified in the CAEs from proteomic study.

E) Distribution of relative standard deviation (RSD) values for quantitative analysis in CAEs during proteomics.

F) Distribution of peptide lengths in the proteomic analysis.

Figure S4





**Fig. S4. Cellular uptake of** **Centella Asiatica-derived exosomes (CAEs) monitored by flow cytometry in RAW 264.7 and NCM460 cells.**

A) RAW 264.7 cells were incubated with PKH 26-CAEs (5 μg/mL) for 1, 2, 4, and 6 h.

1. NCM460 cells were incubated with PKH 26-CAEs (5 μg/mL) for 1, 2, 4, and 6 h.

Data were presented as mean ± SD, with n ≥ 3. **p* <0.05, ***p*<0.01, and *****p*<0.0001.

Figure S5





**Fig. S5. Effects of inhibitors on the cellular uptake of** **Centella Asiatica-derived exosomes (CAEs).**

Flow cytometric analysis was performed to evaluate the effects of Chlorpromazine (10 μg/mL), Methyl-β-cyclodextrin (M-β-CD) (5 μM), Filipin (50 μg/mL), and Amiloride hydrochloride (AMH) (3 mM) inhibitors on the cellular uptake of PKH 26-CAEs (5 μg/mL) after 6-h treatment. (These histogram bars are compared against a negative reference for gating PKH 26-positive cells).

A) RAW 264.7 cells were incubated with PKH 26-CAEs and those inhibitors.

B) NCM460 cells were incubated with PKH 26-CAEs and those inhibitors.

The data were representative for three independent experiments.

Figure S6





**Fig. S6. *In vivo* distribution of** **Centella Asiatica-derived exosomes (CAEs) in mice.**

A) The heart, liver, spleen, lung and kidney were collected from control mice and Dextran sulfate sodium (DSS, 2.5%)-induced acute colitis mice at 0-, 1-, 2-, 4-, 6-, 12- and 24-h post oral administration of DiR-labeled CAEs (10 mg/kg). The data were representative for three independent experiments.

B) *In vivo* distribution of the plant-derived protein RbcL after a 6-h oral administration of CAEs (10 mg/kg) in mice. RbcL levels were measured using an ELISA kit in various tissues (heart, liver, spleen, lung, and kidney), gastrointestinal tract segments (stomach, small intestine, and colon), and blood from control mice and DSS-induced acute colitis mice. Data were presented as mean ± SD, with n ≥ 3. **p* <0.05 and *****p*<0.0001.

Figure S7


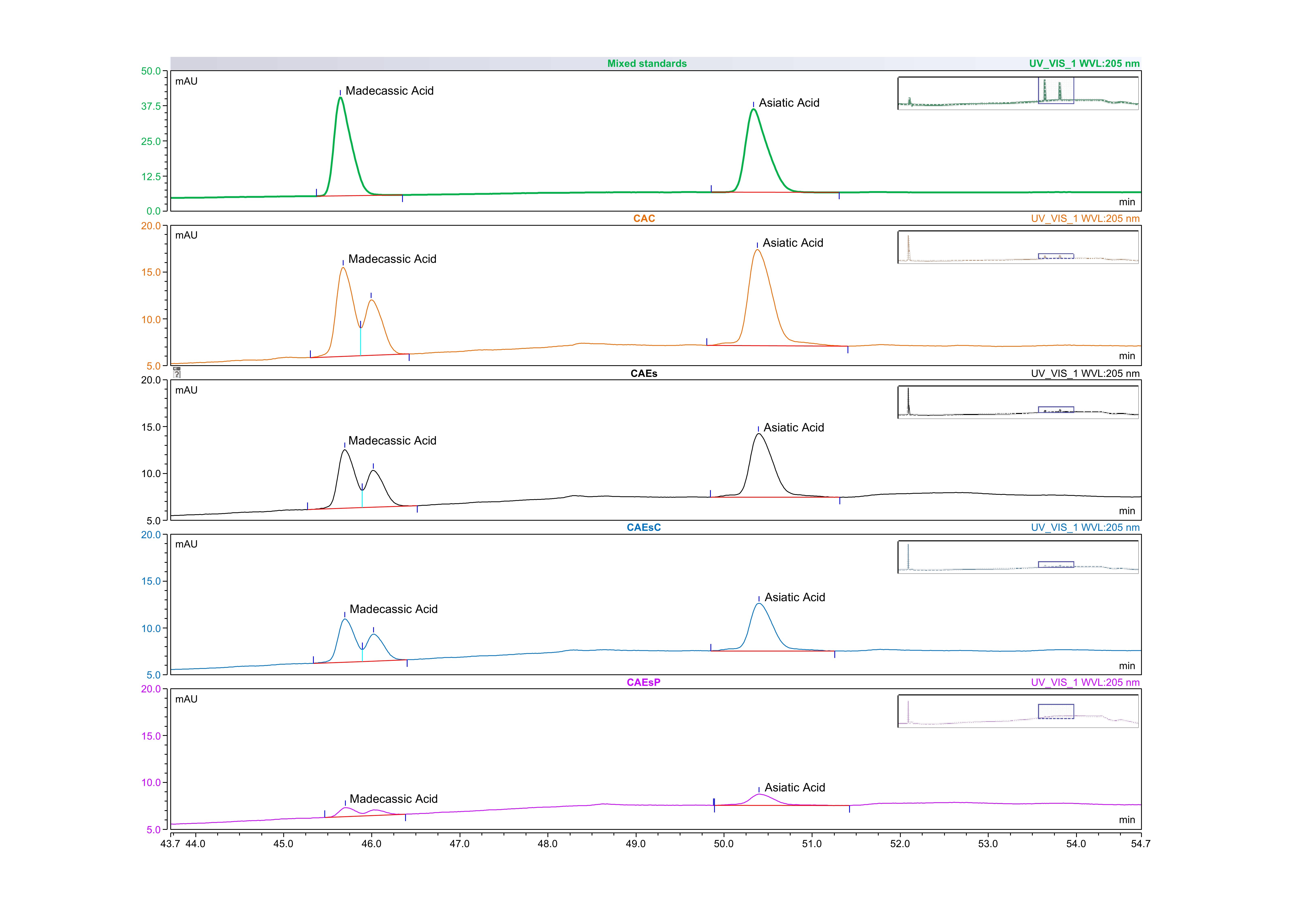


**Fig. S7. Quantitative analysis of** **Madecassic acid and Asiatic acid from Centella Asiatica fractions.**

High-performance liquid chromatography (HPLC) was used to identify and quantitify major phytochemicals from thouse Centella Asiatica fractions, including Madecassic acid and Asiatic acid. The chromatographic profiles were utilized to determine the content of Madecassic acid and Asiatic acid in Centella Asiatica-derived exosomes (CAEs), Centella Asiatica-derived exosomal proteins (CAEsP), Centella Asiatica-derived exosomal compounds (CAEsC), and Centella Asiatica crushing solution (CAC) using the peak area method.

The data were representative for three independent experiments.

Figure S8





**Fig. S8. Quantitative analysis of miRNAs from Centella Asiatica-derived exosomes (CAEs) fractions.**

Quantitative real-time PCR (qRT-PCR) analysis was used to detemine the levels of miRNAs (A: fve-miR396c-3p, B: aof-miR159, and C: aof-miR396b) from Centella Asiatica-derived exosomes (CAEs), Centella Asiatica-derived exosomal proteins (CAEsP), and Centella Asiatica-derived exosomal compounds (CAEsC). Data are presented as mean ± SD, with n ≥ 3. Statistical significance is denoted by asterisks, **p* <0.05 and ***p*<0.01, as well as no statistical significance is indicated by ns.

Figure S9





**Fig. S9. Effects of Centella Asiatica-derived exosomes (CAEs) fractions on** **macrophage polarization.**

A) Cell cytotoxicity was evaluated in RAW 264.7 cells for 8 h with lactate dehydrogenase (LDH) assay. Data were presented as mean ± SD, with n ≥ 3.

B) RAW 264.7 cells were stimulated with LPS alone or co-treated with Centella Asiatica-derived exosomal compounds (CAEsC, 5 μg/mL, dose from pre-separation), Centella Asiatica-derived exosomal proteins (CAEsP, 5 μg/mL, dose from pre-separation) and CAEs (5 μg/mL). Flow cytometry was used to evaluate macrophage polarization. The percentage of CD86^+^ cells (M1 region) is indicated in each panel (These histogram bars compared against a negative reference for gating CD86-positive cells). The data were representative for three independent experiments.

**Figure S10**





**Fig. S10. Therapeutic efficacy of Centella Asiatica-related extracts** **in acute colitis mice.**

A) Colons were harvested from mice after a 10-day treatment regimen. Groups include Control (CON, healthy mouse), Dextran sulfate sodium (DSS, 2.5%), Centella Asiatica-derived exosomes (CAEs, 10 mg/kg/day), Centella Asiatica crushing solution (CAC, 10 mg/kg/day), Asiatic acid (20 mg/kg/day), Asiaticoside (60 mg/kg/day), Madecassic acid (20 mg/kg/day), Madecassoside (60 mg/kg/day), 5-Aminosalicylic acid (5-ASA, 100 mg/kg/day), and Sulfasalazine (SAS, 450 mg/kg/day).

B) Statistical analyasis of colon length in mice. Data are presented as mean ± SD, with n ≥ 3. Statistical significance is denoted by asterisks, *****p* <0.0001.

Fig. S11





**Figure S11.** **Effects of Centella Asiatica-derived exosomes (CAEs) on transcriptional profiles of B cells in colon tissue.**

A) Colons were harvested from mice after CAEs treatment, and transcriptional profiles of colonic B cells were determined by single-cell RNA sequencing. Uniform Manifold Approximation and Projection (UMAP) analysis depicting the clustering of colonic B cells.

B) UMAP algorithm for the classification and visualization of cellular clusters in Control (PBS), Dextran sulfate sodium (DSS, 2.5%), and CAEs (10 mg/kg/day) groups.

C) The most highly expressed genes within each of the five distinct B cells clusters identified by UMAP.

The data were representative for three independent experiments.

Fig. S12





**Figure S12. Effects of Centella Asiatica-derived exosomes (CAEs) on transcriptional profiles of T cells in colon tissue.**

A) Colons were harvested from mice after CAEs treatment, and transcriptional profiles of colonic T cells were determined by single-cell RNA sequencing. Uniform Manifold Approximation and Projection (UMAP) analysis depicting the clustering of colonic T cells.

B) UMAP algorithm for the classification and visualization of cellular clusters in Control (PBS), Dextran sulfate sodium (DSS, 2.5%), and CAEs (10 mg/kg/day) groups.

C) The most highly expressed genes across the five principal T cells clusters identified by UMAP.

The data were representative for three independent experiments.

Fig. S13





**Figure S13. Effects of Centella Asiatica-derived exosomes (CAEs) in Goblet cells and intestinal mucus**

A) Colons were harvested from mice after CAEs treatment and quantitive analysis of Goblet cells were conducted by single-cell RNA sequencing. Goblet cells were identified based on the expression of the gene marker *MUC*. Uniform Manifold Approximation and Projection (UMAP) depicting the clustering of Goblet cells. The data were representative for three independent experiments.

B) Gene expression profiles related to goblet cells, intestinal mucus, and intestinal barriers (MUCs and MMPs family) in colon tissues of each group, including Control (CON, PBS), Dextran sulfate sodium (DSS, 2.5%), and CAEs (10 mg/kg/day). Data are presented as mean ± SD, with n ≥ 3. **p* <0.05, ***p*<0.01, ****p*<0.001, and *****p*<0.0001.

Fig. S14





**Figure S14. Identification and validation of the potential targets of aof-miR396b.**

Matching analysis between aof-miR396b and key genes (*Cd74*, *Satb1*, *Rpl5*, *Foxp1*, *Lars2*, *Peak1*, *Rpl36*, *Gphn*, *Mef2c*, *Bank1*, *Cd83*, and *Rpl23a*) with the database (*rnamtom.net*).

Fig. S15

**

**

**Figure S15. Identification and validation of the potential targets of** **fve-miR396c-3p.**

Matching analysis between fve-miR396c-3p and key genes (*Cd74*, *Satb1*, *Rpl5*, *Foxp1*, *Lars2*, *Peak1*, *Rpl36*, *Gphn*, *Mef2c*, *Bank1*, *Cd83*, and *Rpl23a*) with the database (*rnamtom.net*).

Fig. S16





**Figure S16.** **Therapeutic efficacy of microRNAs in acute colitis mice.**

A) The schematic flowchart delineating the methodology for treatment protocol.

B) Colons were harvested from mice after a 10-day treatment and representative images of colon tissue gross morphology were captured. The experimental cohorts include a healthy control (CON, PBS) group, Dextran sulfate sodium (DSS, 2.5%)-induced colitis group, aof-miR396b (40 nanomoles per administration) group, fve-miR396c-3p (40 nanomoles per administration) group, and aof-miR159 (40 nanomoles per administration) group, as well as a 5-Aminosalicylic acid (5-ASA, 100 mg/kg/day) group.

C) Quantitative analysis of colon length in mice. Data are presented as mean ± SD, with n ≥ 3. Statistical significance is denoted by asterisks, ***p*<0.01 and ****p* <0.001, as well as no statistical significance is indicated by ns.

Fig. S17





**Figure S17. Effects of microRNAs on transcriptional profiles of colon tissue.**

Colons were harvested from mice after microRNAs treatment and quantitive analysis of gene levels were conducted by RNA sequencing. The experimental cohorts include a healthy control (CON, PBS) group, Dextran sulfate sodium (DSS, 2.5%)-induced colitis group, *aof-miR396b* (40 nanomoles per administration) group, and *fve-miR396c-3p* (40 nanomoles per administration) group.

A) Volcano plots of Differentially expressed genes (DEGs) between the DSS and *aof-miR396b* groups.

B) Volcano plots of DEGs between the DSS and *fve-miR396c-3p* groups.

C) The expression levels of key genes (*Cd74*, *Satb1*, *Rpl5*, *Foxp1*, *Lars2*, *Peak1*, *Rpl36*, *Gphn*, *Mef2c*, *Bank1*, *Cd83*, and *Rpl23a*) were analyzed.

Data are presented as mean ± SD, with n ≥ 3.

Fig. S18





**Figure S18. Effect of microRNAs on transcriptional profiles of macrophage.**

RAW 264.7 cells were transfected with *aof-miR396b* and *fve-miR396c-3p* using lipid-based transfection reagent and then induced with Lipopolysaccharides (LPS, 200 ng/mL). Subsequently, the cells were subjected to RNA sequencing analysis.

A) Volcano plots of Differentially expressed genes (DEGs) between the LPS and *aof-miR396b* groups.

B) Volcano plots of DEGs between the LPS and *fve-miR396c-3p* groups.

C) The expression levels of key genes (*Cd74*, *Satb1*, *Rpl5*, *Foxp1*, *Lars2*, *Peak1*, *Rpl36*, *Gphn*, *Mef2c*, *Bank1*, *Cd83*, and *Rpl23a*) were analyzed.

Data are presented as mean ± SD, with n ≥ 3.

Fig. S19





**Figure S19. Therapeutic potential of gut microbiota in pseudo-germ-free (PGF) mice with induced ulcerative colitis (UC).**

A) Colons were harvested from mice after a 10-day treatment regimen, including Control (CON, PBS, healthy mouse), Dextran sulfate sodium (DSS, 2.5% DSS for inducing UC in PGF mice), PGF (untreated PGF mice), and treatments with oral gavage of specific bacterial strains: *Blautia hansenii*, *Pseudoflavonifractor capillosus*, *Alloprevotella rava*, *Romboutsia sedimentorum*, *Flavonifractor plautii*, and *Parabacteroides merdae*, as well as interventions with 5-Aminosalicylic acid (5-ASA, 100 mg/kg/day) and Sulfasalazine (SAS, 450 mg/kg/day) in DSS-induced PGF mice.

B) Quantitative analysis of colon length in mice. Data are presented as mean ± SD, with n ≥ 3. Statistical significance is denoted by asterisks, **p* <0.05, ***p*<0.01, and ****p*<0.001.

Fig. S20





**Figure S20. Effects of Centella Asiatica-derived exosomes (CAEs) on the growth of gut microbiota *in vitro*.**

*Salmonella enterica*, *Klebsiella pneumoniae* and *Blautia hansenii* were treated with different concentrations of CAEs (0, 0.01, 0.1, and 1 mg/mL) at different time points (0-24 h). Optical density at 600 nm (OD_600_) was recorded using an automated microplate reader.

A-C) Real-time monitoring of microbiota growth during a 20-24 h incubation period *in vitro*.

D-F) End-point measurement of microbiota growth at 20-24 h *in vitro*.

G-I) Microbiota cultured to the endpoint were diluted (10^-6^) and plated on solid agar.

Data are presented as mean ± SD, with n ≥ 3. **p*<0.05, ***p*<0.01, and ****p* <0.001, as well as no statistical significance is indicated by ns.

Fig. S21





**Figure S21.** **Assessment of the long-tern stability of Centella Asiatica-derived exosomes (CAEs).**

CAEs were stored at -80°C for 180 days and subsequently thawed to assess their long-term stability. A) Analysis of Zeta Potential for CAEs across a spectrum of temperatures, as well as that in (B) artificial gastric juice (AGJ) and artificial intestinal fluids (AIF).

C-D) Quantitative determination of protein concentrations within CAEs under varying conditions.

E-F) Characterization of the nanoscale size of CAEs under different conditions.

Data are presented as mean ± SD, with n ≥ 3.

Table S1

**Table S1. List of Centella Asiatica-derived exosomes (CAEs) phytochemicals**

| No. | *t*_R_ (min) | Adducts | MS^1^ | Error  (ppm) | MS^2^ | Molecular formula | Identity | Classes |
| --- | --- | --- | --- | --- | --- | --- | --- | --- |
| 1 | 1.28 | [M−H]^−^ | 503.1598 | −3.9 | 341.1097,323.0978,221.0665,179.05863,161.0446,113.0237 | C_18_H_32_O_16_ | Maltotriose | Polysaccharides |
| 2 | 2.01 | [M−H]^−^ | 191.0207 | 5.1 | 111.0088,87.0089,85.0298 | C_16_H_18_O_9_ | Citric Acid | Phenolic acids |
| 3 | 2.48 | [M−H]^−^ | 117.0193 | 3.1 | 117.0197;101.0611 | C_4_H_6_O_4_ | Amber Acid | Glycosides |
| 4 | 8.9 | [M−H]^−^ | 461.0752 | 5.5 | 285.0412,229.0510,113.0229 | C_21_H_18_O_12_ | Kaempferol-3-*O*-*β*-D-glucuronide | Flavonoids |
| 5 | 9.13 | [M−H]^−^ | 601.1203 | 0.7 | 233.0677,179.0378 | C_28_H_26_O_15_ | Irbic acid or isomer | Phenolic acids |
| 6 | 11.12 | [M−H]^−^ | 601.1193 | −1.0 | 233.0688 | C_28_H_26_O_15_ | Irbic acid or isomer | Phenolic acids |
| 7 | 17.44 | [M−H]^−^ | 973.5047 | −2.6 | 503.3433 | C_48_H_78_O_20_ | Madecassoside* | Triterpenes |
| 8 | 18.9 | [M−H]^−^ | 957.5043 | 0.3 | 487.3434,469.1569,409.1293,323.1024,247.0822 | C_48_H_78_O_19_ | Asiaticoside * | Triterpenes |
| 9 | 19.75 | [M−H]^−^ | 973.5000 | 1.1 | 503.3324 | C_48_H_78_O_20_ | Madecassoside isomer | Triterpenes |
| 10 | 20.06 | [M−H]^−^ | 957.5086 | 2.2 | 487.3326 | C_48_H_78_O_19_ | Isoasiaticoside/Centellasaponin J/Centellasaponin C/Centellasaponin D/Scheffoleoside A/Centellasaponin A/Isothankuniside | Triterpenes |
| 11 | 20.54 | [M−H]^−^ | 957.5043 | −2.3 | 487.3436 | C_48_H_78_O_19_ | Isoasiaticoside/Centellasaponin J/Centellasaponin C/Centellasaponin D/Scheffoleoside A/Centellasaponin A/Isothankuniside | Triterpenes |
| 12 | 20.71 | [M−H]^−^ | 711.3959 | 0.3 | 665.3937,575.2990,503.3354,485.3399 | C_37_H_60_O_13_ | 8-oxy-10,11-dihydroxy-9-hexamethyl-tetradecahydro-picene-carboxylic acid | Triterpenes |
| 13 | 21.11 | [M−H]^−^ | 329.2354 | −2.0 | 283.2653,229.1396,211.1358,171.1041,139.1129 | C_18_H_34_O_5_ | Pinellic acid | Fatty Acids |
| 14 | 21.36 | [M−H]^−^ | 665.3925 | −1.1 | 665.4032,649.4080 | C_36_H_58_O_11_ | Chebuloside II | Triterpenes |
| 15 | 21.63 | [M−H]^−^ | 501.3199 | −4.5 | 471.3133,455.3196,321.2093 | C_30_H_46_O_6_ | Medicagenic acid/Retigeric acid B/Catsanogenin | Triterpenes |
| 16 | 21.9 | [M−H]^−^ | 941.5115 | 0.9 | 469.1568,367.1242,323.0987,247.0824,161.0468 | C_48_H_78_O_18_ | Asiaticoside D/Asiaticoside F | Triterpenes |
| 17 | 21.93 | [M−H]^−^ | 501.3215 | −1.3 | 501.3242,169.1035 | C_30_H_48_O_7_ | Medicagenic acid/Retigeric acid B/Catsanogenin | Triterpenes |
| 18 | 22.35 | [M−H]^−^ | 503.3388 | 2.0 | 503.3408,437.3044,391.3041,389.2892 | C_30_H_48_O_6_ | Madecassic acid* | Triterpenes |
| 19 | 23.17 | [M−H]^−^ | 487.3434 | 1.0 | 487.3445 | C_30_H_48_O_5_ | Asiatic acid* | Triterpenes |
| 20 | 23.32 | [M−H]^−^ | 501.3213 | −1.7 | 471.3103,317.0025 | C_30_H_46_O_6_ | Medicagenic acid/Retigeric acid B/Catsanogenin | Triterpenes |
| 21 | 24.2 | [M−H]^−^ | 487.3419 | 5.5 | 487.3456 | C_30_H_48_O_5_ | Isoasiatic acid/Centellasapogenol A/Madasiatic acid/Euscaphic acid | Triterpenes |

Note：“*” identified by authentic reference.

Table S2

**Table S2. List of Centella Asiatica-derived exosomes (CAEs) miRNAs**

| No. | mature_miRNA_ID |
| --- | --- |
| 1 | fve-miR396c-3p |
| 2 | aof-miR159 |
| 3 | aof-miR396b |
| 4 | csi-miR403b-3p |
| 5 | aof-miR166d |
| 6 | csi-miR396f-5p |
| 7 | aof-miR319a |
| 8 | aof-miR166a |
| 9 | aof-miR167a |
| 10 | pab-miR167b |
| 11 | aof-miR393b |
| 12 | sly-miR166c-3p |
| 13 | cas-miR166a |
| 14 | eun-miR172c-5p |
| 15 | aof-miR156a |
| 16 | cas-miR393-5p |
| 17 | aof-miR156b |
| 18 | aof-miR166b |
| 19 | aof-miR167c |
| 20 | ata-miR166c-3p |
| 21 | csi-miR393c-3p |
| 22 | fve-miR159a-5p |
| 23 | aof-miR164 |
| 24 | aof-miR319b |
| 25 | mdm-miR171q |
| 26 | csi-miR395c-3p |
| 27 | aof-miR160c |
| 28 | cas-miR171c-3p |
| 29 | csi-miR156c-3p |
| 30 | fve-miR156g-3p |
| 31 | vca-miR156a-3p |
| 32 | cas-miR159b-3p |
| 33 | zma-miR156g-3p |
| 34 | aof-miR168a |
| 35 | aof-miR398 |
| 36 | aof-miR408 |
| 37 | aof-miR479 |
| 38 | ata-miR399a-3p |
| 39 | bra-miR168c-5p |
| 40 | cas-miR159a |
| 41 | cas-miR390a-3p |
| 42 | cca-miR396a-3p |
| 43 | csi-miR477e-5p |
| 44 | eun-miR167b-3p |
| 45 | gma-miR403b |
| 46 | pgi-miR2118 |
| 47 | aof-miR171a |
| 48 | aof-miR172 |
| 49 | aof-miR393a |
| 50 | aof-miR394 |
| 51 | bna-miR167b |
| 52 | csi-miR159c-3p |
| 53 | eun-miR160-3p |
| 54 | lja-miR166-3p |
| 55 | lus-miR159b |
| 56 | pab-miR858b |
| 57 | sly-miR168a-3p |
| 58 | sly-miR319c-3p |
| 59 | ssp-miR827 |
| 60 | vca-miR396-3p |
| 61 | aau-miR168 |
| 62 | aqc-miR159 |
| 63 | ata-miR169d-3p |
| 64 | cas-miR162a |
| 65 | cas-miR166b |
| 66 | cas-miR172a-5p |
| 67 | cca-miR396c |
| 68 | cpa-miR167c |
| 69 | csi-miR166c-5p |
| 70 | csi-miR169r-5p |
| 71 | csi-miR395b-5p |
| 72 | csi-miR396d-3p |
| 73 | fve-miR167d |
| 74 | fve-miR535b |
| 75 | gma-miR156r |
| 76 | lus-miR396d |
| 77 | osa-miR166i-5p |
| 78 | aly-miR3445-3p.1 |
| 79 | aof-miR166c |
| 80 | aof-miR168b |
| 81 | aof-miR171b |
| 82 | aof-miR390 |
| 83 | aof-miR395a |
| 84 | aof-miR396a |
| 85 | aof-miR399a |
| 86 | aof-miR535 |
| 87 | cas-miR172c |
| 88 | cas-miR403 |
| 89 | cas-miR828 |
| 90 | cas-miR858 |
| 91 | cme-miR399a |
| 92 | cpa-miR8146 |
| 93 | csi-miR166b-5p |
| 94 | csi-miR167d-3p |
| 95 | csi-miR168-3p |
| 96 | csi-miR171c-5p |
| 97 | csi-miR403b-5p |
| 98 | csi-miR482d-5p |
| 99 | fve-miR164c |
| 100 | gma-miR156s |
| 101 | gma-miR159e-5p |
| 102 | gra-miR7504g |
| 103 | lja-miR168-3p |
| 104 | mdm-miR396a |
| 105 | osa-miR159f |
| 106 | osa-miR5538 |
| 107 | pab-miR319j |
| 108 | ppt-miR894 |
| 109 | sly-miR167b-5p |
| 110 | sly-miR9475-3p |
| 111 | sof-miR159e |
| 112 | zma-miR171a-5p |
| 113 | aly-miR3447-3p |
| 114 | aly-miR828-5p |
| 115 | aly-miR833-5p |
| 116 | ama-miR156 |
| 117 | aof-miR167b |
| 118 | aof-miR169c |
| 119 | aof-miR171c |
| 120 | aof-miR477a |
| 121 | ata-miR156d-3p |
| 122 | ata-miR164a-5p |
| 123 | ata-miR166d-5p |
| 124 | ata-miR167f-5p |
| 125 | ata-miR168-5p |
| 126 | ata-miR169c-5p |
| 127 | ata-miR169h-5p |
| 128 | ata-miR396b-5p |
| 129 | ata-miR396c-3p |
| 130 | ata-miR5168-3p |
| 131 | ath-miR2933b |
| 132 | ath-miR5638a |
| 133 | ath-miR5658 |
| 134 | ath-miR8175 |
| 135 | atr-miR171b |
| 136 | atr-miR8577 |
| 137 | bdi-miR159b-3p.1 |
| 138 | bdi-miR166e-3p |
| 139 | bdi-miR319b-3p |
| 140 | bdi-miR5174d-3p |
| 141 | bna-miR399c |
| 142 | bra-miR164c-5p |
| 143 | cas-miR156b-3p |
| 144 | cas-miR166f-3p |
| 145 | cas-miR167a |
| 146 | cas-miR169a |
| 147 | cas-miR169g-5p |
| 148 | cas-miR394 |
| 149 | cas-miR395c-3p |
| 150 | cca-miR156b |
| 151 | cca-miR6111-5p |
| 152 | cme-miR166i |
| 153 | csi-miR156f-5p |
| 154 | csi-miR164a-3p |
| 155 | csi-miR166f-3p |
| 156 | csi-miR166k-3p |
| 157 | csi-miR167c-3p |
| 158 | csi-miR172b-5p |
| 159 | csi-miR393a |
| 160 | csi-miR477d-5p |
| 161 | csi-miR858-3p |
| 162 | eun-miR159-5p |
| 163 | eun-miR827-3p |
| 164 | fve-miR156e |
| 165 | fve-miR156h |
| 166 | fve-miR159b |
| 167 | fve-miR162-3p |
| 168 | fve-miR169e |
| 169 | fve-miR530 |
| 170 | ghr-miR169b |
| 171 | gma-miR10441 |
| 172 | gma-miR1531-5p |
| 173 | gma-miR167k |
| 174 | gma-miR396e |
| 175 | gma-miR4385 |
| 176 | gma-miR4398 |
| 177 | gma-miR5678 |
| 178 | gma-miR9767 |
| 179 | gra-miR167c |
| 180 | gra-miR7502d |
| 181 | gra-miR7504m |
| 182 | gra-miR8752 |
| 183 | hbr-miR396a |
| 184 | hvu-miR156b |
| 185 | lja-miR11090e-5p |
| 186 | lja-miR11108n-3p |
| 187 | lja-miR11155c-3p |
| 188 | lja-miR11155e-5p |
| 189 | lja-miR1507b |
| 190 | lus-miR395e |
| 191 | mdm-miR156ae |
| 192 | mdm-miR391 |
| 193 | mes-miR160h |
| 194 | mes-miR166i |
| 195 | mes-miR319h |
| 196 | mes-miR397b |
| 197 | mes-miR828b |
| 198 | mtr-miR156b-3p |
| 199 | mtr-miR166b |
| 200 | mtr-miR172d-5p |
| 201 | mtr-miR2666 |
| 202 | mtr-miR319c-3p |
| 203 | mtr-miR5236e |
| 204 | mtr-miR5750 |
| 205 | nta-miR160d |
| 206 | osa-miR1439 |
| 207 | osa-miR166e-3p |
| 208 | osa-miR166i-3p |
| 209 | osa-miR2094-3p |
| 210 | osa-miR5801a |
| 211 | pab-miR11450 |
| 212 | pab-miR11452 |
| 213 | pab-miR156ab |
| 214 | pab-miR156k |
| 215 | pab-miR156o |
| 216 | pab-miR159a |
| 217 | pab-miR159d |
| 218 | pab-miR166j |
| 219 | pab-miR319e |
| 220 | pab-miR319h |
| 221 | pab-miR396a-5p |
| 222 | pab-miR396t |
| 223 | pab-miR399c |
| 224 | pde-miR3712 |
| 225 | pgi-miR6138 |
| 226 | ppe-miR2111d |
| 227 | ppe-miR396a |
| 228 | pta-miR159a |
| 229 | ptc-miR166p |
| 230 | ptc-miR167h-5p |
| 231 | ptc-miR319i |
| 232 | sbi-miR5565g-3p |
| 233 | sly-miR166c-5p |
| 234 | sly-miR171e |
| 235 | sly-miR172c |
| 236 | sly-miR482e-3p |
| 237 | sly-miR6027-5p |
| 238 | sly-miR827 |
| 239 | ssl-miR399 |
| 240 | stu-miR156d-3p |
| 241 | stu-miR396-3p |
| 242 | stu-miR397-5p |
| 243 | stu-miR8036-3p |
| 244 | tae-miR9773 |
| 245 | vca-miR166a-5p |
| 246 | vvi-miR399g |
| 247 | zma-miR156k-3p |
| 248 | zma-miR396g-3p |

Table S3

**Table S3. List of Centella Asiatica-derived exosomes (CAEs) proteins**

| No. | Gene Name |
| --- | --- |
| 1 | rbcL |
| 2 | atpA |
| 3 | petA |
| 4 | ndhH |
| 5 | atpB |
| 6 | rps3 |
| 7 | PFK |
| 8 | rps4 |
| 9 | atpE |
| 10 | atpF |
| 11 | MLO |
| 12 | ndhA |
| 13 | ndhF |
| 14 | ndhI |
| 15 | ndhJ |
| 16 | ndhK |
| 17 | psaA |
| 18 | psaC |
| 19 | psbH |
| 20 | rpl20 |
| 21 | rpoA |
| 22 | rpoC1 |
| 23 | rpoC2 |
| 24 | rps16 |
| 25 | ycf1 |
| 26 | 4CL1 |
| 27 | accD |
| 28 | atp1 |
| 29 | GAPC2 |
| 30 | HSP70 |
| 31 | infA |
| 32 | nad7 |
| 33 | ndhD |
| 34 | petB |
| 35 | petD |
| 36 | PFP-BETA |
| 37 | psaB |
| 38 | psbA |
| 39 | psbB |
| 40 | psbE |
| 41 | rpl16 |
| 42 | rpl22 |
| 43 | rpoB |
| 44 | rps15 |
| 45 | rps2 |
| 46 | rps8 |
| 47 | 179B |
| 48 | 4CL |
| 49 | 4CL10 |
| 50 | 4CL7 |
| 51 | AACT |
| 52 | ACC1-1 |
| 53 | ACO |
| 54 | AFP |
| 55 | AG4045_000122 |
| 56 | AG4045_000136 |
| 57 | AG4045_001323 |
| 58 | AG4045_001324 |
| 59 | AG4045_001418 |
| 60 | AG4045_001549 |
| 61 | AG4045_001583 |
| 62 | AG4045_001600 |
| 63 | AG4045_001612 |
| 64 | AG4045_001614 |
| 65 | AG4045_001716 |
| 66 | AG4045_001735 |
| 67 | AG4045_002332 |
| 68 | AG4045_002532 |
| 69 | AG4045_002604 |
| 70 | AG4045_003275 |
| 71 | AG4045_003888 |
| 72 | AG4045_003892 |
| 73 | AG4045_003984 |
| 74 | AG4045_004027 |
| 75 | AG4045_004541 |
| 76 | AG4045_004560 |
| 77 | AG4045_004564 |
| 78 | AG4045_004696 |
| 79 | AG4045_004815 |
| 80 | AG4045_004821 |
| 81 | AG4045_004991 |
| 82 | AG4045_005015 |
| 83 | AG4045_005588 |
| 84 | AG4045_005898 |
| 85 | AG4045_006084 |
| 86 | AG4045_006125 |
| 87 | AG4045_006408 |
| 88 | AG4045_006638 |
| 89 | AG4045_006640 |
| 90 | AG4045_006993 |
| 91 | AG4045_007850 |
| 92 | AG4045_007853 |
| 93 | AG4045_008304 |
| 94 | AG4045_008315 |
| 95 | AG4045_008578 |
| 96 | AG4045_008723 |
| 97 | AG4045_008767 |
| 98 | AG4045_008773 |
| 99 | AG4045_009264 |
| 100 | AG4045_009865 |
| 101 | AG4045_009981 |
| 102 | AG4045_009982 |
| 103 | AG4045_009983 |
| 104 | AG4045_010032 |
| 105 | AG4045_010824 |
| 106 | AG4045_010825 |
| 107 | AG4045_011076 |
| 108 | AG4045_011314 |
| 109 | AG4045_011374 |
| 110 | AG4045_011463 |
| 111 | AG4045_011675 |
| 112 | AG4045_012291 |
| 113 | AG4045_012293 |
| 114 | AG4045_012367 |
| 115 | AG4045_012454 |
| 116 | AG4045_013056 |
| 117 | AG4045_013458 |
| 118 | AG4045_013459 |
| 119 | AG4045_013567 |
| 120 | AG4045_013720 |
| 121 | AG4045_013788 |
| 122 | AG4045_013919 |
| 123 | AG4045_014322 |
| 124 | AG4045_014323 |
| 125 | AG4045_014391 |
| 126 | AG4045_014591 |
| 127 | AG4045_015275 |
| 128 | AG4045_015276 |
| 129 | AG4045_015606 |
| 130 | AG4045_016017 |
| 131 | AG4045_016018 |
| 132 | AG4045_016019 |
| 133 | AG4045_016359 |
| 134 | AG4045_016363 |
| 135 | AG4045_016369 |
| 136 | AG4045_016374 |
| 137 | AG4045_016550 |
| 138 | AG4045_016847 |
| 139 | AG4045_016848 |
| 140 | AG4045_016912 |
| 141 | AG4045_017676 |
| 142 | AG4045_017756 |
| 143 | AG4045_018209 |
| 144 | AG4045_018329 |
| 145 | AG4045_018348 |
| 146 | AG4045_018455 |
| 147 | AG4045_018637 |
| 148 | AG4045_018708 |
| 149 | AG4045_018712 |
| 150 | AG4045_018763 |
| 151 | AG4045_018921 |
| 152 | AG4045_019515 |
| 153 | AG4045_019614 |
| 154 | AG4045_020532 |
| 155 | AG4045_020634 |
| 156 | AG4045_020890 |
| 157 | AG4045_021137 |
| 158 | AG4045_021166 |
| 159 | AG4045_021196 |
| 160 | AG4045_021511 |
| 161 | AG4045_021513 |
| 162 | AG4045_021515 |
| 163 | AG4045_021518 |
| 164 | AG4045_021934 |
| 165 | AG4045_022205 |
| 166 | AG4045_022398 |
| 167 | AG4045_022697 |
| 168 | AG4045_022704 |
| 169 | AG4045_023139 |
| 170 | AG4045_023440 |
| 171 | AG4045_023467 |
| 172 | AG4045_023677 |
| 173 | AG4045_023685 |
| 174 | AG4045_024201 |
| 175 | AG4045_024762 |
| 176 | AG4045_025184 |
| 177 | AG4045_025201 |
| 178 | AG4045_025316 |
| 179 | AG4045_025686 |
| 180 | AG4045_026093 |
| 181 | AG4045_026094 |
| 182 | AG4045_026284 |
| 183 | AG4045_026528 |
| 184 | AG4045_026572 |
| 185 | AG4045_026720 |
| 186 | AG4045_026958 |
| 187 | AG4045_026976 |
| 188 | AG4045_026985 |
| 189 | AG4045_026986 |
| 190 | AG4045_027428 |
| 191 | AG4045_027437 |
| 192 | AG4045_027450 |
| 193 | AG4045_027544 |
| 194 | AG4045_027752 |
| 195 | AG4045_028066 |
| 196 | AG4045_028069 |
| 197 | AG4045_028433 |
| 198 | AG4045_028471 |
| 199 | AG4045_028650 |
| 200 | AG4045_028654 |
| 201 | AG4045_029431 |
| 202 | AG4045_029845 |
| 203 | AG4045_029847 |
| 204 | AG4045_030247 |
| 205 | AG4045_030326 |
| 206 | AG4045_030345 |
| 207 | AG4045_030414 |
| 208 | AG4045_030606 |
| 209 | AG4045_030627 |
| 210 | AG4045_030912 |
| 211 | AG4045_031080 |
| 212 | AG4045_031081 |
| 213 | AGPDc1 |
| 214 | AIS1 |
| 215 | AOX2b |
| 216 | ARF1 |
| 217 | At4g26390 |
| 218 | atp4 |
| 219 | atpI |
| 220 | AX110 |
| 221 | C4H-1 |
| 222 | C5-COMT |
| 223 | cab3 |
| 224 | CAM-1 |
| 225 | CAPETRO |
| 226 | CAT1 |
| 227 | CCD1 |
| 228 | CCD1-1 |
| 229 | CCD1-3 |
| 230 | CDPMES |
| 231 | cG6PDH1 |
| 232 | CHXE |
| 233 | clpP |
| 234 | cob |
| 235 | COMT |
| 236 | COMT-S |
| 237 | COX2 |
| 238 | cox3 |
| 239 | CPR2 |
| 240 | CRTISO-2 |
| 241 | CS |
| 242 | cyc07 |
| 243 | CYP73A10 |
| 244 | CYP82H1 |
| 245 | CYP97A |
| 246 | CYP97B3 |
| 247 | CYP98A21 |
| 248 | Dc-Rab8 |
| 249 | DCAR_000200 |
| 250 | DCAR_000223 |
| 251 | DCAR_000295 |
| 252 | DCAR_000299 |
| 253 | DCAR_000300 |
| 254 | DCAR_000378 |
| 255 | DCAR_000389 |
| 256 | DCAR_000400 |
| 257 | DCAR_000426 |
| 258 | DCAR_000446 |
| 259 | DCAR_000455 |
| 260 | DCAR_000474 |
| 261 | DCAR_000504 |
| 262 | DCAR_000505 |
| 263 | DCAR_000511 |
| 264 | DCAR_000520 |
| 265 | DCAR_000552 |
| 266 | DCAR_000568 |
| 267 | DCAR_000584 |
| 268 | DCAR_000587 |
| 269 | DCAR_000596 |
| 270 | DCAR_000604 |
| 271 | DCAR_000608 |
| 272 | DCAR_000615 |
| 273 | DCAR_000619 |
| 274 | DCAR_000638 |
| 275 | DCAR_000646 |
| 276 | DCAR_000648 |
| 277 | DCAR_000665 |
| 278 | DCAR_000671 |
| 279 | DCAR_000673 |
| 280 | DCAR_000676 |
| 281 | DCAR_000686 |
| 282 | DCAR_000689 |
| 283 | DCAR_000691 |
| 284 | DCAR_000729 |
| 285 | DCAR_000732 |
| 286 | DCAR_000743 |
| 287 | DCAR_000753 |
| 288 | DCAR_000791 |
| 289 | DCAR_000796 |
| 290 | DCAR_000802 |
| 291 | DCAR_000814 |
| 292 | DCAR_000816 |
| 293 | DCAR_000820 |
| 294 | DCAR_000836 |
| 295 | DCAR_000840 |
| 296 | DCAR_000854 |
| 297 | DCAR_000858 |
| 298 | DCAR_000890 |
| 299 | DCAR_000895 |
| 300 | DCAR_000922 |
| 301 | DCAR_000932 |
| 302 | DCAR_000942 |
| 303 | DCAR_000953 |
| 304 | DCAR_000959 |
| 305 | DCAR_000966 |
| 306 | DCAR_000972 |
| 307 | DCAR_000973 |
| 308 | DCAR_000997 |
| 309 | DCAR_001031 |
| 310 | DCAR_001047 |
| 311 | DCAR_001057 |
| 312 | DCAR_001116 |
| 313 | DCAR_001127 |
| 314 | DCAR_001133 |
| 315 | DCAR_001144 |
| 316 | DCAR_001157 |
| 317 | DCAR_001159 |
| 318 | DCAR_001160 |
| 319 | DCAR_001175 |
| 320 | DCAR_001198 |
| 321 | DCAR_001207 |
| 322 | DCAR_001208 |
| 323 | DCAR_001211 |
| 324 | DCAR_001214 |
| 325 | DCAR_001249 |
| 326 | DCAR_001263 |
| 327 | DCAR_001279 |
| 328 | DCAR_001290 |
| 329 | DCAR_001294 |
| 330 | DCAR_001295 |
| 331 | DCAR_001310 |
| 332 | DCAR_001322 |
| 333 | DCAR_001326 |
| 334 | DCAR_001327 |
| 335 | DCAR_001360 |
| 336 | DCAR_001366 |
| 337 | DCAR_001377 |
| 338 | DCAR_001381 |
| 339 | DCAR_001384 |
| 340 | DCAR_001388 |
| 341 | DCAR_001393 |
| 342 | DCAR_001395 |
| 343 | DCAR_001426 |
| 344 | DCAR_001434 |
| 345 | DCAR_001441 |
| 346 | DCAR_001444 |
| 347 | DCAR_001445 |
| 348 | DCAR_001451 |
| 349 | DCAR_001464 |
| 350 | DCAR_001465 |
| 351 | DCAR_001480 |
| 352 | DCAR_001503 |
| 353 | DCAR_001509 |
| 354 | DCAR_001513 |
| 355 | DCAR_001518 |
| 356 | DCAR_001529 |
| 357 | DCAR_001545 |
| 358 | DCAR_001550 |
| 359 | DCAR_001554 |
| 360 | DCAR_001565 |
| 361 | DCAR_001569 |
| 362 | DCAR_001571 |
| 363 | DCAR_001579 |
| 364 | DCAR_001583 |
| 365 | DCAR_001595 |
| 366 | DCAR_001596 |
| 367 | DCAR_001620 |
| 368 | DCAR_001621 |
| 369 | DCAR_001629 |
| 370 | DCAR_001630 |
| 371 | DCAR_001639 |
| 372 | DCAR_001640 |
| 373 | DCAR_001655 |
| 374 | DCAR_001662 |
| 375 | DCAR_001668 |
| 376 | DCAR_001704 |
| 377 | DCAR_001705 |
| 378 | DCAR_001715 |
| 379 | DCAR_001727 |
| 380 | DCAR_001729 |
| 381 | DCAR_001745 |
| 382 | DCAR_001748 |
| 383 | DCAR_001763 |
| 384 | DCAR_001770 |
| 385 | DCAR_001777 |
| 386 | DCAR_001786 |
| 387 | DCAR_001787 |
| 388 | DCAR_001799 |
| 389 | DCAR_001811 |
| 390 | DCAR_001820 |
| 391 | DCAR_001823 |
| 392 | DCAR_001855 |
| 393 | DCAR_001869 |
| 394 | DCAR_001875 |
| 395 | DCAR_001880 |
| 396 | DCAR_001888 |
| 397 | DCAR_001897 |
| 398 | DCAR_001898 |
| 399 | DCAR_001900 |
| 400 | DCAR_001912 |
| 401 | DCAR_001913 |
| 402 | DCAR_001927 |
| 403 | DCAR_001929 |
| 404 | DCAR_001930 |
| 405 | DCAR_001933 |
| 406 | DCAR_001954 |
| 407 | DCAR_001959 |
| 408 | DCAR_001961 |
| 409 | DCAR_001963 |
| 410 | DCAR_001968 |
| 411 | DCAR_001976 |
| 412 | DCAR_001979 |
| 413 | DCAR_001982 |
| 414 | DCAR_001983 |
| 415 | DCAR_001985 |
| 416 | DCAR_001986 |
| 417 | DCAR_001987 |
| 418 | DCAR_001999 |
| 419 | DCAR_002024 |
| 420 | DCAR_002025 |
| 421 | DCAR_002026 |
| 422 | DCAR_002027 |
| 423 | DCAR_002029 |
| 424 | DCAR_002034 |
| 425 | DCAR_002036 |
| 426 | DCAR_002037 |
| 427 | DCAR_002050 |
| 428 | DCAR_002051 |
| 429 | DCAR_002061 |
| 430 | DCAR_002067 |
| 431 | DCAR_002085 |
| 432 | DCAR_002090 |
| 433 | DCAR_002094 |
| 434 | DCAR_002100 |
| 435 | DCAR_002102 |
| 436 | DCAR_002111 |
| 437 | DCAR_002119 |
| 438 | DCAR_002122 |
| 439 | DCAR_002126 |
| 440 | DCAR_002130 |
| 441 | DCAR_002144 |
| 442 | DCAR_002146 |
| 443 | DCAR_002150 |
| 444 | DCAR_002153 |
| 445 | DCAR_002161 |
| 446 | DCAR_002178 |
| 447 | DCAR_002198 |
| 448 | DCAR_002199 |
| 449 | DCAR_002200 |
| 450 | DCAR_002212 |
| 451 | DCAR_002217 |
| 452 | DCAR_002225 |
| 453 | DCAR_002226 |
| 454 | DCAR_002240 |
| 455 | DCAR_002243 |
| 456 | DCAR_002244 |
| 457 | DCAR_002254 |
| 458 | DCAR_002260 |
| 459 | DCAR_002265 |
| 460 | DCAR_002272 |
| 461 | DCAR_002280 |
| 462 | DCAR_002283 |
| 463 | DCAR_002286 |
| 464 | DCAR_002290 |
| 465 | DCAR_002306 |
| 466 | DCAR_002318 |
| 467 | DCAR_002322 |
| 468 | DCAR_002344 |
| 469 | DCAR_002355 |
| 470 | DCAR_002359 |
| 471 | DCAR_002365 |
| 472 | DCAR_002369 |
| 473 | DCAR_002386 |
| 474 | DCAR_002390 |
| 475 | DCAR_002394 |
| 476 | DCAR_002396 |
| 477 | DCAR_002397 |
| 478 | DCAR_002425 |
| 479 | DCAR_002434 |
| 480 | DCAR_002436 |
| 481 | DCAR_002455 |
| 482 | DCAR_002465 |
| 483 | DCAR_002466 |
| 484 | DCAR_002470 |
| 485 | DCAR_002471 |
| 486 | DCAR_002490 |
| 487 | DCAR_002504 |
| 488 | DCAR_002512 |
| 489 | DCAR_002518 |
| 490 | DCAR_002520 |
| 491 | DCAR_002529 |
| 492 | DCAR_002539 |
| 493 | DCAR_002548 |
| 494 | DCAR_002557 |
| 495 | DCAR_002565 |
| 496 | DCAR_002576 |
| 497 | DCAR_002583 |
| 498 | DCAR_002588 |
| 499 | DCAR_002589 |
| 500 | DCAR_002593 |
| 501 | DCAR_002598 |
| 502 | DCAR_002601 |
| 503 | DCAR_002602 |
| 504 | DCAR_002603 |
| 505 | DCAR_002610 |
| 506 | DCAR_002619 |
| 507 | DCAR_002637 |
| 508 | DCAR_002638 |
| 509 | DCAR_002639 |
| 510 | DCAR_002652 |
| 511 | DCAR_002654 |
| 512 | DCAR_002668 |
| 513 | DCAR_002678 |
| 514 | DCAR_002687 |
| 515 | DCAR_002691 |
| 516 | DCAR_002695 |
| 517 | DCAR_002698 |
| 518 | DCAR_002710 |
| 519 | DCAR_002711 |
| 520 | DCAR_002713 |
| 521 | DCAR_002727 |
| 522 | DCAR_002729 |
| 523 | DCAR_002733 |
| 524 | DCAR_002749 |
| 525 | DCAR_002766 |
| 526 | DCAR_002780 |
| 527 | DCAR_002781 |
| 528 | DCAR_002788 |
| 529 | DCAR_002816 |
| 530 | DCAR_002820 |
| 531 | DCAR_002823 |
| 532 | DCAR_002833 |
| 533 | DCAR_002834 |
| 534 | DCAR_002836 |
| 535 | DCAR_002843 |
| 536 | DCAR_002847 |
| 537 | DCAR_002849 |
| 538 | DCAR_002854 |
| 539 | DCAR_002855 |
| 540 | DCAR_002856 |
| 541 | DCAR_002857 |
| 542 | DCAR_002879 |
| 543 | DCAR_002891 |
| 544 | DCAR_002915 |
| 545 | DCAR_002917 |
| 546 | DCAR_002923 |
| 547 | DCAR_002932 |
| 548 | DCAR_002933 |
| 549 | DCAR_002934 |
| 550 | DCAR_002935 |
| 551 | DCAR_002936 |
| 552 | DCAR_002937 |
| 553 | DCAR_002940 |
| 554 | DCAR_002946 |
| 555 | DCAR_002950 |
| 556 | DCAR_002962 |
| 557 | DCAR_002971 |
| 558 | DCAR_002974 |
| 559 | DCAR_002977 |
| 560 | DCAR_002984 |
| 561 | DCAR_002997 |
| 562 | DCAR_003005 |
| 563 | DCAR_003011 |
| 564 | DCAR_003019 |
| 565 | DCAR_003035 |
| 566 | DCAR_003048 |
| 567 | DCAR_003051 |
| 568 | DCAR_003055 |
| 569 | DCAR_003056 |
| 570 | DCAR_003072 |
| 571 | DCAR_003075 |
| 572 | DCAR_003077 |
| 573 | DCAR_003081 |
| 574 | DCAR_003086 |
| 575 | DCAR_003115 |
| 576 | DCAR_003126 |
| 577 | DCAR_003129 |
| 578 | DCAR_003140 |
| 579 | DCAR_003141 |
| 580 | DCAR_003145 |
| 581 | DCAR_003155 |
| 582 | DCAR_003157 |
| 583 | DCAR_003161 |
| 584 | DCAR_003164 |
| 585 | DCAR_003165 |
| 586 | DCAR_003166 |
| 587 | DCAR_003173 |
| 588 | DCAR_003178 |
| 589 | DCAR_003213 |
| 590 | DCAR_003217 |
| 591 | DCAR_003225 |
| 592 | DCAR_003226 |
| 593 | DCAR_003231 |
| 594 | DCAR_003232 |
| 595 | DCAR_003235 |
| 596 | DCAR_003239 |
| 597 | DCAR_003251 |
| 598 | DCAR_003253 |
| 599 | DCAR_003261 |
| 600 | DCAR_003264 |
| 601 | DCAR_003266 |
| 602 | DCAR_003270 |
| 603 | DCAR_003273 |
| 604 | DCAR_003298 |
| 605 | DCAR_003310 |
| 606 | DCAR_003314 |
| 607 | DCAR_003321 |
| 608 | DCAR_003324 |
| 609 | DCAR_003326 |
| 610 | DCAR_003353 |
| 611 | DCAR_003358 |
| 612 | DCAR_003377 |
| 613 | DCAR_003393 |
| 614 | DCAR_003401 |
| 615 | DCAR_003411 |
| 616 | DCAR_003413 |
| 617 | DCAR_003415 |
| 618 | DCAR_003420 |
| 619 | DCAR_003427 |
| 620 | DCAR_003434 |
| 621 | DCAR_003437 |
| 622 | DCAR_003449 |
| 623 | DCAR_003456 |
| 624 | DCAR_003469 |
| 625 | DCAR_003470 |
| 626 | DCAR_003472 |
| 627 | DCAR_003474 |
| 628 | DCAR_003475 |
| 629 | DCAR_003476 |
| 630 | DCAR_003478 |
| 631 | DCAR_003482 |
| 632 | DCAR_003490 |
| 633 | DCAR_003506 |
| 634 | DCAR_003512 |
| 635 | DCAR_003523 |
| 636 | DCAR_003526 |
| 637 | DCAR_003532 |
| 638 | DCAR_003551 |
| 639 | DCAR_003564 |
| 640 | DCAR_003567 |
| 641 | DCAR_003572 |
| 642 | DCAR_003573 |
| 643 | DCAR_003580 |
| 644 | DCAR_003587 |
| 645 | DCAR_003592 |
| 646 | DCAR_003602 |
| 647 | DCAR_003604 |
| 648 | DCAR_003605 |
| 649 | DCAR_003613 |
| 650 | DCAR_003618 |
| 651 | DCAR_003639 |
| 652 | DCAR_003642 |
| 653 | DCAR_003654 |
| 654 | DCAR_003658 |
| 655 | DCAR_003665 |
| 656 | DCAR_003671 |
| 657 | DCAR_003673 |
| 658 | DCAR_003678 |
| 659 | DCAR_003679 |
| 660 | DCAR_003680 |
| 661 | DCAR_003681 |
| 662 | DCAR_003683 |
| 663 | DCAR_003686 |
| 664 | DCAR_003692 |
| 665 | DCAR_003693 |
| 666 | DCAR_003695 |
| 667 | DCAR_003699 |
| 668 | DCAR_003707 |
| 669 | DCAR_003711 |
| 670 | DCAR_003713 |
| 671 | DCAR_003714 |
| 672 | DCAR_003723 |
| 673 | DCAR_003725 |
| 674 | DCAR_003728 |
| 675 | DCAR_003741 |
| 676 | DCAR_003751 |
| 677 | DCAR_003771 |
| 678 | DCAR_003772 |
| 679 | DCAR_003775 |
| 680 | DCAR_003780 |
| 681 | DCAR_003781 |
| 682 | DCAR_003796 |
| 683 | DCAR_003798 |
| 684 | DCAR_003828 |
| 685 | DCAR_003831 |
| 686 | DCAR_003832 |
| 687 | DCAR_003854 |
| 688 | DCAR_003864 |
| 689 | DCAR_003880 |
| 690 | DCAR_003881 |
| 691 | DCAR_003883 |
| 692 | DCAR_003892 |
| 693 | DCAR_003902 |
| 694 | DCAR_003906 |
| 695 | DCAR_003909 |
| 696 | DCAR_003920 |
| 697 | DCAR_003925 |
| 698 | DCAR_003949 |
| 699 | DCAR_003950 |
| 700 | DCAR_003952 |
| 701 | DCAR_003961 |
| 702 | DCAR_003964 |
| 703 | DCAR_003967 |
| 704 | DCAR_003977 |
| 705 | DCAR_003991 |
| 706 | DCAR_004001 |
| 707 | DCAR_004011 |
| 708 | DCAR_004016 |
| 709 | DCAR_004025 |
| 710 | DCAR_004062 |
| 711 | DCAR_004076 |
| 712 | DCAR_004078 |
| 713 | DCAR_004087 |
| 714 | DCAR_004114 |
| 715 | DCAR_004125 |
| 716 | DCAR_004130 |
| 717 | DCAR_004142 |
| 718 | DCAR_004162 |
| 719 | DCAR_004163 |
| 720 | DCAR_004165 |
| 721 | DCAR_004175 |
| 722 | DCAR_004182 |
| 723 | DCAR_004183 |
| 724 | DCAR_004200 |
| 725 | DCAR_004207 |
| 726 | DCAR_004213 |
| 727 | DCAR_004217 |
| 728 | DCAR_004220 |
| 729 | DCAR_004227 |
| 730 | DCAR_004232 |
| 731 | DCAR_004238 |
| 732 | DCAR_004262 |
| 733 | DCAR_004263 |
| 734 | DCAR_004287 |
| 735 | DCAR_004313 |
| 736 | DCAR_004333 |
| 737 | DCAR_004348 |
| 738 | DCAR_004367 |
| 739 | DCAR_004370 |
| 740 | DCAR_004376 |
| 741 | DCAR_004380 |
| 742 | DCAR_004430 |
| 743 | DCAR_004435 |
| 744 | DCAR_004440 |
| 745 | DCAR_004452 |
| 746 | DCAR_004453 |
| 747 | DCAR_004493 |
| 748 | DCAR_004495 |
| 749 | DCAR_004503 |
| 750 | DCAR_004512 |
| 751 | DCAR_004515 |
| 752 | DCAR_004517 |
| 753 | DCAR_004519 |
| 754 | DCAR_004529 |
| 755 | DCAR_004536 |
| 756 | DCAR_004543 |
| 757 | DCAR_004550 |
| 758 | DCAR_004564 |
| 759 | DCAR_004565 |
| 760 | DCAR_004572 |
| 761 | DCAR_004575 |
| 762 | DCAR_004578 |
| 763 | DCAR_004584 |
| 764 | DCAR_004586 |
| 765 | DCAR_004589 |
| 766 | DCAR_004600 |
| 767 | DCAR_004619 |
| 768 | DCAR_004625 |
| 769 | DCAR_004626 |
| 770 | DCAR_004633 |
| 771 | DCAR_004635 |
| 772 | DCAR_004642 |
| 773 | DCAR_004651 |
| 774 | DCAR_004665 |
| 775 | DCAR_004677 |
| 776 | DCAR_004683 |
| 777 | DCAR_004694 |
| 778 | DCAR_004710 |
| 779 | DCAR_004732 |
| 780 | DCAR_004746 |
| 781 | DCAR_004749 |
| 782 | DCAR_004763 |
| 783 | DCAR_004764 |
| 784 | DCAR_004771 |
| 785 | DCAR_004792 |
| 786 | DCAR_004794 |
| 787 | DCAR_004804 |
| 788 | DCAR_004808 |
| 789 | DCAR_004833 |
| 790 | DCAR_004863 |
| 791 | DCAR_004868 |
| 792 | DCAR_004870 |
| 793 | DCAR_004881 |
| 794 | DCAR_004889 |
| 795 | DCAR_004906 |
| 796 | DCAR_004914 |
| 797 | DCAR_004919 |
| 798 | DCAR_004929 |
| 799 | DCAR_005022 |
| 800 | DCAR_005043 |
| 801 | DCAR_005050 |
| 802 | DCAR_005122 |
| 803 | DCAR_005223 |
| 804 | DCAR_005274 |
| 805 | DCAR_005278 |
| 806 | DCAR_005280 |
| 807 | DCAR_005281 |
| 808 | DCAR_005296 |
| 809 | DCAR_005308 |
| 810 | DCAR_005312 |
| 811 | DCAR_005314 |
| 812 | DCAR_005350 |
| 813 | DCAR_005355 |
| 814 | DCAR_005361 |
| 815 | DCAR_005371 |
| 816 | DCAR_005387 |
| 817 | DCAR_005394 |
| 818 | DCAR_005415 |
| 819 | DCAR_005419 |
| 820 | DCAR_005435 |
| 821 | DCAR_005442 |
| 822 | DCAR_005460 |
| 823 | DCAR_005462 |
| 824 | DCAR_005484 |
| 825 | DCAR_005503 |
| 826 | DCAR_005509 |
| 827 | DCAR_005510 |
| 828 | DCAR_005517 |
| 829 | DCAR_005524 |
| 830 | DCAR_005525 |
| 831 | DCAR_005540 |
| 832 | DCAR_005543 |
| 833 | DCAR_005553 |
| 834 | DCAR_005556 |
| 835 | DCAR_005562 |
| 836 | DCAR_005564 |
| 837 | DCAR_005567 |
| 838 | DCAR_005569 |
| 839 | DCAR_005583 |
| 840 | DCAR_005585 |
| 841 | DCAR_005593 |
| 842 | DCAR_005596 |
| 843 | DCAR_005597 |
| 844 | DCAR_005602 |
| 845 | DCAR_005608 |
| 846 | DCAR_005619 |
| 847 | DCAR_005620 |
| 848 | DCAR_005627 |
| 849 | DCAR_005634 |
| 850 | DCAR_005658 |
| 851 | DCAR_005675 |
| 852 | DCAR_005677 |
| 853 | DCAR_005682 |
| 854 | DCAR_005687 |
| 855 | DCAR_005690 |
| 856 | DCAR_005702 |
| 857 | DCAR_005714 |
| 858 | DCAR_005716 |
| 859 | DCAR_005719 |
| 860 | DCAR_005742 |
| 861 | DCAR_005751 |
| 862 | DCAR_005757 |
| 863 | DCAR_005766 |
| 864 | DCAR_005773 |
| 865 | DCAR_005782 |
| 866 | DCAR_005783 |
| 867 | DCAR_005799 |
| 868 | DCAR_005803 |
| 869 | DCAR_005805 |
| 870 | DCAR_005808 |
| 871 | DCAR_005820 |
| 872 | DCAR_005822 |
| 873 | DCAR_005823 |
| 874 | DCAR_005827 |
| 875 | DCAR_005850 |
| 876 | DCAR_005858 |
| 877 | DCAR_005870 |
| 878 | DCAR_005879 |
| 879 | DCAR_005883 |
| 880 | DCAR_005884 |
| 881 | DCAR_005941 |
| 882 | DCAR_005942 |
| 883 | DCAR_005945 |
| 884 | DCAR_005948 |
| 885 | DCAR_005977 |
| 886 | DCAR_005985 |
| 887 | DCAR_005991 |
| 888 | DCAR_006002 |
| 889 | DCAR_006006 |
| 890 | DCAR_006018 |
| 891 | DCAR_006022 |
| 892 | DCAR_006029 |
| 893 | DCAR_006049 |
| 894 | DCAR_006064 |
| 895 | DCAR_006112 |
| 896 | DCAR_006129 |
| 897 | DCAR_006134 |
| 898 | DCAR_006140 |
| 899 | DCAR_006147 |
| 900 | DCAR_006148 |
| 901 | DCAR_006149 |
| 902 | DCAR_006150 |
| 903 | DCAR_006154 |
| 904 | DCAR_006155 |
| 905 | DCAR_006162 |
| 906 | DCAR_006171 |
| 907 | DCAR_006178 |
| 908 | DCAR_006211 |
| 909 | DCAR_006218 |
| 910 | DCAR_006219 |
| 911 | DCAR_006220 |
| 912 | DCAR_006223 |
| 913 | DCAR_006256 |
| 914 | DCAR_006257 |
| 915 | DCAR_006262 |
| 916 | DCAR_006268 |
| 917 | DCAR_006269 |
| 918 | DCAR_006270 |
| 919 | DCAR_006280 |
| 920 | DCAR_006281 |
| 921 | DCAR_006290 |
| 922 | DCAR_006299 |
| 923 | DCAR_006300 |
| 924 | DCAR_006303 |
| 925 | DCAR_006304 |
| 926 | DCAR_006308 |
| 927 | DCAR_006311 |
| 928 | DCAR_006330 |
| 929 | DCAR_006337 |
| 930 | DCAR_006348 |
| 931 | DCAR_006352 |
| 932 | DCAR_006377 |
| 933 | DCAR_006393 |
| 934 | DCAR_006401 |
| 935 | DCAR_006405 |
| 936 | DCAR_006406 |
| 937 | DCAR_006411 |
| 938 | DCAR_006415 |
| 939 | DCAR_006417 |
| 940 | DCAR_006425 |
| 941 | DCAR_006426 |
| 942 | DCAR_006431 |
| 943 | DCAR_006435 |
| 944 | DCAR_006439 |
| 945 | DCAR_006442 |
| 946 | DCAR_006463 |
| 947 | DCAR_006465 |
| 948 | DCAR_006478 |
| 949 | DCAR_006479 |
| 950 | DCAR_006484 |
| 951 | DCAR_006502 |
| 952 | DCAR_006504 |
| 953 | DCAR_006507 |
| 954 | DCAR_006513 |
| 955 | DCAR_006514 |
| 956 | DCAR_006530 |
| 957 | DCAR_006536 |
| 958 | DCAR_006537 |
| 959 | DCAR_006540 |
| 960 | DCAR_006543 |
| 961 | DCAR_006545 |
| 962 | DCAR_006556 |
| 963 | DCAR_006568 |
| 964 | DCAR_006585 |
| 965 | DCAR_006586 |
| 966 | DCAR_006587 |
| 967 | DCAR_006592 |
| 968 | DCAR_006596 |
| 969 | DCAR_006604 |
| 970 | DCAR_006606 |
| 971 | DCAR_006611 |
| 972 | DCAR_006615 |
| 973 | DCAR_006625 |
| 974 | DCAR_006627 |
| 975 | DCAR_006630 |
| 976 | DCAR_006631 |
| 977 | DCAR_006636 |
| 978 | DCAR_006647 |
| 979 | DCAR_006648 |
| 980 | DCAR_006655 |
| 981 | DCAR_006660 |
| 982 | DCAR_006665 |
| 983 | DCAR_006667 |
| 984 | DCAR_006668 |
| 985 | DCAR_006669 |
| 986 | DCAR_006673 |
| 987 | DCAR_006689 |
| 988 | DCAR_006698 |
| 989 | DCAR_006707 |
| 990 | DCAR_006713 |
| 991 | DCAR_006734 |
| 992 | DCAR_006735 |
| 993 | DCAR_006747 |
| 994 | DCAR_006750 |
| 995 | DCAR_006766 |
| 996 | DCAR_006768 |
| 997 | DCAR_006771 |
| 998 | DCAR_006803 |
| 999 | DCAR_006805 |
| 1000 | DCAR_006806 |
| 1001 | DCAR_006810 |
| 1002 | DCAR_006811 |
| 1003 | DCAR_006814 |
| 1004 | DCAR_006822 |
| 1005 | DCAR_006836 |
| 1006 | DCAR_006851 |
| 1007 | DCAR_006858 |
| 1008 | DCAR_006860 |
| 1009 | DCAR_006866 |
| 1010 | DCAR_006870 |
| 1011 | DCAR_006872 |
| 1012 | DCAR_006875 |
| 1013 | DCAR_006882 |
| 1014 | DCAR_006884 |
| 1015 | DCAR_006891 |
| 1016 | DCAR_006900 |
| 1017 | DCAR_006909 |
| 1018 | DCAR_006913 |
| 1019 | DCAR_006917 |
| 1020 | DCAR_006920 |
| 1021 | DCAR_006928 |
| 1022 | DCAR_006940 |
| 1023 | DCAR_006943 |
| 1024 | DCAR_006944 |
| 1025 | DCAR_006948 |
| 1026 | DCAR_006965 |
| 1027 | DCAR_006975 |
| 1028 | DCAR_006977 |
| 1029 | DCAR_006981 |
| 1030 | DCAR_006993 |
| 1031 | DCAR_007003 |
| 1032 | DCAR_007010 |
| 1033 | DCAR_007011 |
| 1034 | DCAR_007013 |
| 1035 | DCAR_007030 |
| 1036 | DCAR_007034 |
| 1037 | DCAR_007053 |
| 1038 | DCAR_007064 |
| 1039 | DCAR_007094 |
| 1040 | DCAR_007098 |
| 1041 | DCAR_007102 |
| 1042 | DCAR_007115 |
| 1043 | DCAR_007116 |
| 1044 | DCAR_007119 |
| 1045 | DCAR_007135 |
| 1046 | DCAR_007168 |
| 1047 | DCAR_007169 |
| 1048 | DCAR_007181 |
| 1049 | DCAR_007191 |
| 1050 | DCAR_007192 |
| 1051 | DCAR_007200 |
| 1052 | DCAR_007221 |
| 1053 | DCAR_007240 |
| 1054 | DCAR_007253 |
| 1055 | DCAR_007260 |
| 1056 | DCAR_007265 |
| 1057 | DCAR_007274 |
| 1058 | DCAR_007282 |
| 1059 | DCAR_007289 |
| 1060 | DCAR_007294 |
| 1061 | DCAR_007304 |
| 1062 | DCAR_007311 |
| 1063 | DCAR_007312 |
| 1064 | DCAR_007313 |
| 1065 | DCAR_007325 |
| 1066 | DCAR_007328 |
| 1067 | DCAR_007332 |
| 1068 | DCAR_007335 |
| 1069 | DCAR_007342 |
| 1070 | DCAR_007353 |
| 1071 | DCAR_007356 |
| 1072 | DCAR_007359 |
| 1073 | DCAR_007368 |
| 1074 | DCAR_007378 |
| 1075 | DCAR_007392 |
| 1076 | DCAR_007398 |
| 1077 | DCAR_007400 |
| 1078 | DCAR_007406 |
| 1079 | DCAR_007407 |
| 1080 | DCAR_007424 |
| 1081 | DCAR_007432 |
| 1082 | DCAR_007441 |
| 1083 | DCAR_007447 |
| 1084 | DCAR_007453 |
| 1085 | DCAR_007457 |
| 1086 | DCAR_007458 |
| 1087 | DCAR_007471 |
| 1088 | DCAR_007477 |
| 1089 | DCAR_007481 |
| 1090 | DCAR_007484 |
| 1091 | DCAR_007485 |
| 1092 | DCAR_007493 |
| 1093 | DCAR_007498 |
| 1094 | DCAR_007522 |
| 1095 | DCAR_007527 |
| 1096 | DCAR_007543 |
| 1097 | DCAR_007547 |
| 1098 | DCAR_007555 |
| 1099 | DCAR_007560 |
| 1100 | DCAR_007564 |
| 1101 | DCAR_007566 |
| 1102 | DCAR_007568 |
| 1103 | DCAR_007585 |
| 1104 | DCAR_007592 |
| 1105 | DCAR_007607 |
| 1106 | DCAR_007610 |
| 1107 | DCAR_007616 |
| 1108 | DCAR_007627 |
| 1109 | DCAR_007628 |
| 1110 | DCAR_007633 |
| 1111 | DCAR_007637 |
| 1112 | DCAR_007639 |
| 1113 | DCAR_007640 |
| 1114 | DCAR_007641 |
| 1115 | DCAR_007646 |
| 1116 | DCAR_007652 |
| 1117 | DCAR_007654 |
| 1118 | DCAR_007657 |
| 1119 | DCAR_007664 |
| 1120 | DCAR_007670 |
| 1121 | DCAR_007671 |
| 1122 | DCAR_007674 |
| 1123 | DCAR_007707 |
| 1124 | DCAR_007712 |
| 1125 | DCAR_007728 |
| 1126 | DCAR_007757 |
| 1127 | DCAR_007758 |
| 1128 | DCAR_007811 |
| 1129 | DCAR_007814 |
| 1130 | DCAR_007822 |
| 1131 | DCAR_007824 |
| 1132 | DCAR_007826 |
| 1133 | DCAR_007829 |
| 1134 | DCAR_007836 |
| 1135 | DCAR_007838 |
| 1136 | DCAR_007856 |
| 1137 | DCAR_007867 |
| 1138 | DCAR_007868 |
| 1139 | DCAR_007881 |
| 1140 | DCAR_007882 |
| 1141 | DCAR_007888 |
| 1142 | DCAR_007905 |
| 1143 | DCAR_007906 |
| 1144 | DCAR_007907 |
| 1145 | DCAR_007937 |
| 1146 | DCAR_007943 |
| 1147 | DCAR_007969 |
| 1148 | DCAR_008009 |
| 1149 | DCAR_008047 |
| 1150 | DCAR_008058 |
| 1151 | DCAR_008065 |
| 1152 | DCAR_008071 |
| 1153 | DCAR_008074 |
| 1154 | DCAR_008082 |
| 1155 | DCAR_008098 |
| 1156 | DCAR_008142 |
| 1157 | DCAR_008153 |
| 1158 | DCAR_008154 |
| 1159 | DCAR_008161 |
| 1160 | DCAR_008165 |
| 1161 | DCAR_008168 |
| 1162 | DCAR_008170 |
| 1163 | DCAR_008188 |
| 1164 | DCAR_008190 |
| 1165 | DCAR_008192 |
| 1166 | DCAR_008201 |
| 1167 | DCAR_008205 |
| 1168 | DCAR_008221 |
| 1169 | DCAR_008222 |
| 1170 | DCAR_008235 |
| 1171 | DCAR_008241 |
| 1172 | DCAR_008256 |
| 1173 | DCAR_008268 |
| 1174 | DCAR_008277 |
| 1175 | DCAR_008285 |
| 1176 | DCAR_008286 |
| 1177 | DCAR_008288 |
| 1178 | DCAR_008292 |
| 1179 | DCAR_008293 |
| 1180 | DCAR_008296 |
| 1181 | DCAR_008326 |
| 1182 | DCAR_008327 |
| 1183 | DCAR_008330 |
| 1184 | DCAR_008336 |
| 1185 | DCAR_008337 |
| 1186 | DCAR_008346 |
| 1187 | DCAR_008367 |
| 1188 | DCAR_008371 |
| 1189 | DCAR_008373 |
| 1190 | DCAR_008377 |
| 1191 | DCAR_008379 |
| 1192 | DCAR_008400 |
| 1193 | DCAR_008409 |
| 1194 | DCAR_008415 |
| 1195 | DCAR_008419 |
| 1196 | DCAR_008427 |
| 1197 | DCAR_008450 |
| 1198 | DCAR_008457 |
| 1199 | DCAR_008462 |
| 1200 | DCAR_008465 |
| 1201 | DCAR_008467 |
| 1202 | DCAR_008493 |
| 1203 | DCAR_008500 |
| 1204 | DCAR_008522 |
| 1205 | DCAR_008527 |
| 1206 | DCAR_008546 |
| 1207 | DCAR_008553 |
| 1208 | DCAR_008566 |
| 1209 | DCAR_008580 |
| 1210 | DCAR_008599 |
| 1211 | DCAR_008601 |
| 1212 | DCAR_008603 |
| 1213 | DCAR_008614 |
| 1214 | DCAR_008616 |
| 1215 | DCAR_008620 |
| 1216 | DCAR_008623 |
| 1217 | DCAR_008624 |
| 1218 | DCAR_008627 |
| 1219 | DCAR_008636 |
| 1220 | DCAR_008641 |
| 1221 | DCAR_008646 |
| 1222 | DCAR_008652 |
| 1223 | DCAR_008654 |
| 1224 | DCAR_008656 |
| 1225 | DCAR_008661 |
| 1226 | DCAR_008663 |
| 1227 | DCAR_008665 |
| 1228 | DCAR_008690 |
| 1229 | DCAR_008692 |
| 1230 | DCAR_008693 |
| 1231 | DCAR_008695 |
| 1232 | DCAR_008704 |
| 1233 | DCAR_008713 |
| 1234 | DCAR_008715 |
| 1235 | DCAR_008728 |
| 1236 | DCAR_008733 |
| 1237 | DCAR_008748 |
| 1238 | DCAR_008755 |
| 1239 | DCAR_008760 |
| 1240 | DCAR_008786 |
| 1241 | DCAR_008790 |
| 1242 | DCAR_008802 |
| 1243 | DCAR_008808 |
| 1244 | DCAR_008811 |
| 1245 | DCAR_008813 |
| 1246 | DCAR_008867 |
| 1247 | DCAR_008869 |
| 1248 | DCAR_008873 |
| 1249 | DCAR_008883 |
| 1250 | DCAR_008897 |
| 1251 | DCAR_008911 |
| 1252 | DCAR_008916 |
| 1253 | DCAR_008919 |
| 1254 | DCAR_008921 |
| 1255 | DCAR_008946 |
| 1256 | DCAR_008953 |
| 1257 | DCAR_008960 |
| 1258 | DCAR_008962 |
| 1259 | DCAR_008963 |
| 1260 | DCAR_008965 |
| 1261 | DCAR_008978 |
| 1262 | DCAR_008980 |
| 1263 | DCAR_008989 |
| 1264 | DCAR_008993 |
| 1265 | DCAR_009004 |
| 1266 | DCAR_009012 |
| 1267 | DCAR_009019 |
| 1268 | DCAR_009034 |
| 1269 | DCAR_009036 |
| 1270 | DCAR_009037 |
| 1271 | DCAR_009044 |
| 1272 | DCAR_009049 |
| 1273 | DCAR_009057 |
| 1274 | DCAR_009060 |
| 1275 | DCAR_009062 |
| 1276 | DCAR_009074 |
| 1277 | DCAR_009108 |
| 1278 | DCAR_009112 |
| 1279 | DCAR_009121 |
| 1280 | DCAR_009123 |
| 1281 | DCAR_009136 |
| 1282 | DCAR_009140 |
| 1283 | DCAR_009150 |
| 1284 | DCAR_009200 |
| 1285 | DCAR_009226 |
| 1286 | DCAR_009242 |
| 1287 | DCAR_009244 |
| 1288 | DCAR_009258 |
| 1289 | DCAR_009261 |
| 1290 | DCAR_009300 |
| 1291 | DCAR_009307 |
| 1292 | DCAR_009319 |
| 1293 | DCAR_009326 |
| 1294 | DCAR_009328 |
| 1295 | DCAR_009330 |
| 1296 | DCAR_009331 |
| 1297 | DCAR_009337 |
| 1298 | DCAR_009364 |
| 1299 | DCAR_009374 |
| 1300 | DCAR_009382 |
| 1301 | DCAR_009385 |
| 1302 | DCAR_009391 |
| 1303 | DCAR_009392 |
| 1304 | DCAR_009404 |
| 1305 | DCAR_009406 |
| 1306 | DCAR_009409 |
| 1307 | DCAR_009424 |
| 1308 | DCAR_009425 |
| 1309 | DCAR_009426 |
| 1310 | DCAR_009451 |
| 1311 | DCAR_009455 |
| 1312 | DCAR_009460 |
| 1313 | DCAR_009465 |
| 1314 | DCAR_009480 |
| 1315 | DCAR_009482 |
| 1316 | DCAR_009484 |
| 1317 | DCAR_009493 |
| 1318 | DCAR_009496 |
| 1319 | DCAR_009497 |
| 1320 | DCAR_009514 |
| 1321 | DCAR_009515 |
| 1322 | DCAR_009520 |
| 1323 | DCAR_009523 |
| 1324 | DCAR_009533 |
| 1325 | DCAR_009544 |
| 1326 | DCAR_009547 |
| 1327 | DCAR_009561 |
| 1328 | DCAR_009571 |
| 1329 | DCAR_009578 |
| 1330 | DCAR_009585 |
| 1331 | DCAR_009588 |
| 1332 | DCAR_009594 |
| 1333 | DCAR_009600 |
| 1334 | DCAR_009617 |
| 1335 | DCAR_009619 |
| 1336 | DCAR_009623 |
| 1337 | DCAR_009627 |
| 1338 | DCAR_009630 |
| 1339 | DCAR_009652 |
| 1340 | DCAR_009654 |
| 1341 | DCAR_009655 |
| 1342 | DCAR_009687 |
| 1343 | DCAR_009688 |
| 1344 | DCAR_009692 |
| 1345 | DCAR_009700 |
| 1346 | DCAR_009714 |
| 1347 | DCAR_009724 |
| 1348 | DCAR_009735 |
| 1349 | DCAR_009754 |
| 1350 | DCAR_009762 |
| 1351 | DCAR_009771 |
| 1352 | DCAR_009775 |
| 1353 | DCAR_009778 |
| 1354 | DCAR_009779 |
| 1355 | DCAR_009790 |
| 1356 | DCAR_009791 |
| 1357 | DCAR_009820 |
| 1358 | DCAR_009828 |
| 1359 | DCAR_009834 |
| 1360 | DCAR_009842 |
| 1361 | DCAR_009846 |
| 1362 | DCAR_009850 |
| 1363 | DCAR_009854 |
| 1364 | DCAR_009862 |
| 1365 | DCAR_009899 |
| 1366 | DCAR_009903 |
| 1367 | DCAR_009904 |
| 1368 | DCAR_009906 |
| 1369 | DCAR_009913 |
| 1370 | DCAR_009921 |
| 1371 | DCAR_009922 |
| 1372 | DCAR_009930 |
| 1373 | DCAR_009934 |
| 1374 | DCAR_009937 |
| 1375 | DCAR_009942 |
| 1376 | DCAR_009950 |
| 1377 | DCAR_009952 |
| 1378 | DCAR_009955 |
| 1379 | DCAR_009970 |
| 1380 | DCAR_009992 |
| 1381 | DCAR_010001 |
| 1382 | DCAR_010008 |
| 1383 | DCAR_010009 |
| 1384 | DCAR_010033 |
| 1385 | DCAR_010053 |
| 1386 | DCAR_010058 |
| 1387 | DCAR_010061 |
| 1388 | DCAR_010069 |
| 1389 | DCAR_010076 |
| 1390 | DCAR_010077 |
| 1391 | DCAR_010079 |
| 1392 | DCAR_010088 |
| 1393 | DCAR_010118 |
| 1394 | DCAR_010124 |
| 1395 | DCAR_010138 |
| 1396 | DCAR_010146 |
| 1397 | DCAR_010170 |
| 1398 | DCAR_010176 |
| 1399 | DCAR_010190 |
| 1400 | DCAR_010202 |
| 1401 | DCAR_010233 |
| 1402 | DCAR_010278 |
| 1403 | DCAR_010287 |
| 1404 | DCAR_010312 |
| 1405 | DCAR_010319 |
| 1406 | DCAR_010340 |
| 1407 | DCAR_010345 |
| 1408 | DCAR_010395 |
| 1409 | DCAR_010396 |
| 1410 | DCAR_010401 |
| 1411 | DCAR_010402 |
| 1412 | DCAR_010405 |
| 1413 | DCAR_010436 |
| 1414 | DCAR_010446 |
| 1415 | DCAR_010454 |
| 1416 | DCAR_010455 |
| 1417 | DCAR_010469 |
| 1418 | DCAR_010489 |
| 1419 | DCAR_010500 |
| 1420 | DCAR_010514 |
| 1421 | DCAR_010538 |
| 1422 | DCAR_010542 |
| 1423 | DCAR_010545 |
| 1424 | DCAR_010557 |
| 1425 | DCAR_010569 |
| 1426 | DCAR_010579 |
| 1427 | DCAR_010582 |
| 1428 | DCAR_010590 |
| 1429 | DCAR_010607 |
| 1430 | DCAR_010617 |
| 1431 | DCAR_010631 |
| 1432 | DCAR_010646 |
| 1433 | DCAR_010650 |
| 1434 | DCAR_010652 |
| 1435 | DCAR_010658 |
| 1436 | DCAR_010659 |
| 1437 | DCAR_010684 |
| 1438 | DCAR_010685 |
| 1439 | DCAR_010687 |
| 1440 | DCAR_010695 |
| 1441 | DCAR_010698 |
| 1442 | DCAR_010720 |
| 1443 | DCAR_010735 |
| 1444 | DCAR_010737 |
| 1445 | DCAR_010772 |
| 1446 | DCAR_010778 |
| 1447 | DCAR_010790 |
| 1448 | DCAR_010835 |
| 1449 | DCAR_010839 |
| 1450 | DCAR_010876 |
| 1451 | DCAR_010882 |
| 1452 | DCAR_010909 |
| 1453 | DCAR_010948 |
| 1454 | DCAR_010953 |
| 1455 | DCAR_010956 |
| 1456 | DCAR_010999 |
| 1457 | DCAR_011000 |
| 1458 | DCAR_011007 |
| 1459 | DCAR_011020 |
| 1460 | DCAR_011034 |
| 1461 | DCAR_011045 |
| 1462 | DCAR_011048 |
| 1463 | DCAR_011053 |
| 1464 | DCAR_011054 |
| 1465 | DCAR_011063 |
| 1466 | DCAR_011074 |
| 1467 | DCAR_011078 |
| 1468 | DCAR_011086 |
| 1469 | DCAR_011088 |
| 1470 | DCAR_011097 |
| 1471 | DCAR_011111 |
| 1472 | DCAR_011132 |
| 1473 | DCAR_011134 |
| 1474 | DCAR_011141 |
| 1475 | DCAR_011148 |
| 1476 | DCAR_011153 |
| 1477 | DCAR_011159 |
| 1478 | DCAR_011162 |
| 1479 | DCAR_011163 |
| 1480 | DCAR_011176 |
| 1481 | DCAR_011180 |
| 1482 | DCAR_011187 |
| 1483 | DCAR_011197 |
| 1484 | DCAR_011208 |
| 1485 | DCAR_011215 |
| 1486 | DCAR_011231 |
| 1487 | DCAR_011234 |
| 1488 | DCAR_011255 |
| 1489 | DCAR_011266 |
| 1490 | DCAR_011283 |
| 1491 | DCAR_011290 |
| 1492 | DCAR_011292 |
| 1493 | DCAR_011295 |
| 1494 | DCAR_011305 |
| 1495 | DCAR_011309 |
| 1496 | DCAR_011317 |
| 1497 | DCAR_011327 |
| 1498 | DCAR_011329 |
| 1499 | DCAR_011346 |
| 1500 | DCAR_011353 |
| 1501 | DCAR_011356 |
| 1502 | DCAR_011383 |
| 1503 | DCAR_011388 |
| 1504 | DCAR_011389 |
| 1505 | DCAR_011404 |
| 1506 | DCAR_011407 |
| 1507 | DCAR_011413 |
| 1508 | DCAR_011444 |
| 1509 | DCAR_011445 |
| 1510 | DCAR_011462 |
| 1511 | DCAR_011466 |
| 1512 | DCAR_011475 |
| 1513 | DCAR_011485 |
| 1514 | DCAR_011497 |
| 1515 | DCAR_011498 |
| 1516 | DCAR_011518 |
| 1517 | DCAR_011528 |
| 1518 | DCAR_011535 |
| 1519 | DCAR_011536 |
| 1520 | DCAR_011550 |
| 1521 | DCAR_011560 |
| 1522 | DCAR_011561 |
| 1523 | DCAR_011564 |
| 1524 | DCAR_011566 |
| 1525 | DCAR_011567 |
| 1526 | DCAR_011581 |
| 1527 | DCAR_011585 |
| 1528 | DCAR_011604 |
| 1529 | DCAR_011606 |
| 1530 | DCAR_011621 |
| 1531 | DCAR_011632 |
| 1532 | DCAR_011652 |
| 1533 | DCAR_011655 |
| 1534 | DCAR_011657 |
| 1535 | DCAR_011660 |
| 1536 | DCAR_011664 |
| 1537 | DCAR_011679 |
| 1538 | DCAR_011685 |
| 1539 | DCAR_011688 |
| 1540 | DCAR_011694 |
| 1541 | DCAR_011702 |
| 1542 | DCAR_011707 |
| 1543 | DCAR_011718 |
| 1544 | DCAR_011723 |
| 1545 | DCAR_011724 |
| 1546 | DCAR_011727 |
| 1547 | DCAR_011731 |
| 1548 | DCAR_011746 |
| 1549 | DCAR_011750 |
| 1550 | DCAR_011765 |
| 1551 | DCAR_011784 |
| 1552 | DCAR_011785 |
| 1553 | DCAR_011789 |
| 1554 | DCAR_011791 |
| 1555 | DCAR_011794 |
| 1556 | DCAR_011799 |
| 1557 | DCAR_011803 |
| 1558 | DCAR_011811 |
| 1559 | DCAR_011827 |
| 1560 | DCAR_011843 |
| 1561 | DCAR_011854 |
| 1562 | DCAR_011855 |
| 1563 | DCAR_011860 |
| 1564 | DCAR_011861 |
| 1565 | DCAR_011866 |
| 1566 | DCAR_011944 |
| 1567 | DCAR_011945 |
| 1568 | DCAR_011948 |
| 1569 | DCAR_011953 |
| 1570 | DCAR_011965 |
| 1571 | DCAR_011968 |
| 1572 | DCAR_011975 |
| 1573 | DCAR_011978 |
| 1574 | DCAR_011979 |
| 1575 | DCAR_011989 |
| 1576 | DCAR_011995 |
| 1577 | DCAR_012011 |
| 1578 | DCAR_012028 |
| 1579 | DCAR_012054 |
| 1580 | DCAR_012060 |
| 1581 | DCAR_012064 |
| 1582 | DCAR_012069 |
| 1583 | DCAR_012070 |
| 1584 | DCAR_012086 |
| 1585 | DCAR_012087 |
| 1586 | DCAR_012088 |
| 1587 | DCAR_012089 |
| 1588 | DCAR_012097 |
| 1589 | DCAR_012103 |
| 1590 | DCAR_012112 |
| 1591 | DCAR_012167 |
| 1592 | DCAR_012175 |
| 1593 | DCAR_012182 |
| 1594 | DCAR_012183 |
| 1595 | DCAR_012192 |
| 1596 | DCAR_012193 |
| 1597 | DCAR_012210 |
| 1598 | DCAR_012230 |
| 1599 | DCAR_012248 |
| 1600 | DCAR_012264 |
| 1601 | DCAR_012278 |
| 1602 | DCAR_012279 |
| 1603 | DCAR_012280 |
| 1604 | DCAR_012281 |
| 1605 | DCAR_012290 |
| 1606 | DCAR_012296 |
| 1607 | DCAR_012334 |
| 1608 | DCAR_012335 |
| 1609 | DCAR_012353 |
| 1610 | DCAR_012360 |
| 1611 | DCAR_012367 |
| 1612 | DCAR_012393 |
| 1613 | DCAR_012395 |
| 1614 | DCAR_012409 |
| 1615 | DCAR_012421 |
| 1616 | DCAR_012436 |
| 1617 | DCAR_012437 |
| 1618 | DCAR_012439 |
| 1619 | DCAR_012460 |
| 1620 | DCAR_012484 |
| 1621 | DCAR_012500 |
| 1622 | DCAR_012520 |
| 1623 | DCAR_012528 |
| 1624 | DCAR_012529 |
| 1625 | DCAR_012547 |
| 1626 | DCAR_012550 |
| 1627 | DCAR_012551 |
| 1628 | DCAR_012561 |
| 1629 | DCAR_012568 |
| 1630 | DCAR_012588 |
| 1631 | DCAR_012618 |
| 1632 | DCAR_012623 |
| 1633 | DCAR_012643 |
| 1634 | DCAR_012647 |
| 1635 | DCAR_012654 |
| 1636 | DCAR_012682 |
| 1637 | DCAR_012683 |
| 1638 | DCAR_012699 |
| 1639 | DCAR_012718 |
| 1640 | DCAR_012725 |
| 1641 | DCAR_012729 |
| 1642 | DCAR_012739 |
| 1643 | DCAR_012771 |
| 1644 | DCAR_012777 |
| 1645 | DCAR_012835 |
| 1646 | DCAR_012841 |
| 1647 | DCAR_012846 |
| 1648 | DCAR_012855 |
| 1649 | DCAR_012862 |
| 1650 | DCAR_012873 |
| 1651 | DCAR_012882 |
| 1652 | DCAR_012886 |
| 1653 | DCAR_012898 |
| 1654 | DCAR_012906 |
| 1655 | DCAR_012937 |
| 1656 | DCAR_012943 |
| 1657 | DCAR_012949 |
| 1658 | DCAR_012950 |
| 1659 | DCAR_012964 |
| 1660 | DCAR_012965 |
| 1661 | DCAR_012969 |
| 1662 | DCAR_012972 |
| 1663 | DCAR_012973 |
| 1664 | DCAR_012974 |
| 1665 | DCAR_013007 |
| 1666 | DCAR_013008 |
| 1667 | DCAR_013009 |
| 1668 | DCAR_013012 |
| 1669 | DCAR_013018 |
| 1670 | DCAR_013027 |
| 1671 | DCAR_013046 |
| 1672 | DCAR_013051 |
| 1673 | DCAR_013059 |
| 1674 | DCAR_013064 |
| 1675 | DCAR_013065 |
| 1676 | DCAR_013066 |
| 1677 | DCAR_013080 |
| 1678 | DCAR_013103 |
| 1679 | DCAR_013117 |
| 1680 | DCAR_013125 |
| 1681 | DCAR_013147 |
| 1682 | DCAR_013150 |
| 1683 | DCAR_013153 |
| 1684 | DCAR_013154 |
| 1685 | DCAR_013168 |
| 1686 | DCAR_013169 |
| 1687 | DCAR_013172 |
| 1688 | DCAR_013174 |
| 1689 | DCAR_013175 |
| 1690 | DCAR_013185 |
| 1691 | DCAR_013195 |
| 1692 | DCAR_013206 |
| 1693 | DCAR_013210 |
| 1694 | DCAR_013214 |
| 1695 | DCAR_013218 |
| 1696 | DCAR_013223 |
| 1697 | DCAR_013246 |
| 1698 | DCAR_013249 |
| 1699 | DCAR_013256 |
| 1700 | DCAR_013257 |
| 1701 | DCAR_013264 |
| 1702 | DCAR_013281 |
| 1703 | DCAR_013282 |
| 1704 | DCAR_013289 |
| 1705 | DCAR_013303 |
| 1706 | DCAR_013318 |
| 1707 | DCAR_013322 |
| 1708 | DCAR_013327 |
| 1709 | DCAR_013330 |
| 1710 | DCAR_013331 |
| 1711 | DCAR_013333 |
| 1712 | DCAR_013343 |
| 1713 | DCAR_013344 |
| 1714 | DCAR_013345 |
| 1715 | DCAR_013349 |
| 1716 | DCAR_013363 |
| 1717 | DCAR_013366 |
| 1718 | DCAR_013368 |
| 1719 | DCAR_013373 |
| 1720 | DCAR_013384 |
| 1721 | DCAR_013387 |
| 1722 | DCAR_013410 |
| 1723 | DCAR_013415 |
| 1724 | DCAR_013424 |
| 1725 | DCAR_013425 |
| 1726 | DCAR_013426 |
| 1727 | DCAR_013448 |
| 1728 | DCAR_013460 |
| 1729 | DCAR_013465 |
| 1730 | DCAR_013468 |
| 1731 | DCAR_013474 |
| 1732 | DCAR_013479 |
| 1733 | DCAR_013485 |
| 1734 | DCAR_013487 |
| 1735 | DCAR_013497 |
| 1736 | DCAR_013498 |
| 1737 | DCAR_013500 |
| 1738 | DCAR_013513 |
| 1739 | DCAR_013514 |
| 1740 | DCAR_013528 |
| 1741 | DCAR_013529 |
| 1742 | DCAR_013530 |
| 1743 | DCAR_013545 |
| 1744 | DCAR_013548 |
| 1745 | DCAR_013553 |
| 1746 | DCAR_013558 |
| 1747 | DCAR_013559 |
| 1748 | DCAR_013561 |
| 1749 | DCAR_013562 |
| 1750 | DCAR_013565 |
| 1751 | DCAR_013569 |
| 1752 | DCAR_013571 |
| 1753 | DCAR_013581 |
| 1754 | DCAR_013593 |
| 1755 | DCAR_013594 |
| 1756 | DCAR_013596 |
| 1757 | DCAR_013597 |
| 1758 | DCAR_013599 |
| 1759 | DCAR_013629 |
| 1760 | DCAR_013635 |
| 1761 | DCAR_013636 |
| 1762 | DCAR_013639 |
| 1763 | DCAR_013654 |
| 1764 | DCAR_013660 |
| 1765 | DCAR_013673 |
| 1766 | DCAR_013678 |
| 1767 | DCAR_013689 |
| 1768 | DCAR_013714 |
| 1769 | DCAR_013719 |
| 1770 | DCAR_013723 |
| 1771 | DCAR_013724 |
| 1772 | DCAR_013727 |
| 1773 | DCAR_013737 |
| 1774 | DCAR_013739 |
| 1775 | DCAR_013741 |
| 1776 | DCAR_013750 |
| 1777 | DCAR_013751 |
| 1778 | DCAR_013752 |
| 1779 | DCAR_013763 |
| 1780 | DCAR_013764 |
| 1781 | DCAR_013765 |
| 1782 | DCAR_013771 |
| 1783 | DCAR_013778 |
| 1784 | DCAR_013782 |
| 1785 | DCAR_013788 |
| 1786 | DCAR_013793 |
| 1787 | DCAR_013805 |
| 1788 | DCAR_013806 |
| 1789 | DCAR_013816 |
| 1790 | DCAR_013817 |
| 1791 | DCAR_013821 |
| 1792 | DCAR_013841 |
| 1793 | DCAR_013844 |
| 1794 | DCAR_013850 |
| 1795 | DCAR_013851 |
| 1796 | DCAR_013857 |
| 1797 | DCAR_013858 |
| 1798 | DCAR_013859 |
| 1799 | DCAR_013873 |
| 1800 | DCAR_013875 |
| 1801 | DCAR_013876 |
| 1802 | DCAR_013880 |
| 1803 | DCAR_013883 |
| 1804 | DCAR_013895 |
| 1805 | DCAR_013907 |
| 1806 | DCAR_013924 |
| 1807 | DCAR_013925 |
| 1808 | DCAR_013926 |
| 1809 | DCAR_013929 |
| 1810 | DCAR_013933 |
| 1811 | DCAR_013938 |
| 1812 | DCAR_013958 |
| 1813 | DCAR_013960 |
| 1814 | DCAR_013972 |
| 1815 | DCAR_013974 |
| 1816 | DCAR_013987 |
| 1817 | DCAR_014008 |
| 1818 | DCAR_014011 |
| 1819 | DCAR_014043 |
| 1820 | DCAR_014049 |
| 1821 | DCAR_014054 |
| 1822 | DCAR_014060 |
| 1823 | DCAR_014073 |
| 1824 | DCAR_014088 |
| 1825 | DCAR_014101 |
| 1826 | DCAR_014128 |
| 1827 | DCAR_014145 |
| 1828 | DCAR_014158 |
| 1829 | DCAR_014159 |
| 1830 | DCAR_014164 |
| 1831 | DCAR_014170 |
| 1832 | DCAR_014174 |
| 1833 | DCAR_014182 |
| 1834 | DCAR_014192 |
| 1835 | DCAR_014206 |
| 1836 | DCAR_014209 |
| 1837 | DCAR_014215 |
| 1838 | DCAR_014224 |
| 1839 | DCAR_014226 |
| 1840 | DCAR_014237 |
| 1841 | DCAR_014239 |
| 1842 | DCAR_014245 |
| 1843 | DCAR_014252 |
| 1844 | DCAR_014271 |
| 1845 | DCAR_014300 |
| 1846 | DCAR_014314 |
| 1847 | DCAR_014335 |
| 1848 | DCAR_014338 |
| 1849 | DCAR_014347 |
| 1850 | DCAR_014358 |
| 1851 | DCAR_014361 |
| 1852 | DCAR_014380 |
| 1853 | DCAR_014387 |
| 1854 | DCAR_014390 |
| 1855 | DCAR_014391 |
| 1856 | DCAR_014392 |
| 1857 | DCAR_014395 |
| 1858 | DCAR_014400 |
| 1859 | DCAR_014409 |
| 1860 | DCAR_014419 |
| 1861 | DCAR_014420 |
| 1862 | DCAR_014429 |
| 1863 | DCAR_014431 |
| 1864 | DCAR_014432 |
| 1865 | DCAR_014443 |
| 1866 | DCAR_014444 |
| 1867 | DCAR_014445 |
| 1868 | DCAR_014447 |
| 1869 | DCAR_014451 |
| 1870 | DCAR_014456 |
| 1871 | DCAR_014463 |
| 1872 | DCAR_014468 |
| 1873 | DCAR_014474 |
| 1874 | DCAR_014477 |
| 1875 | DCAR_014478 |
| 1876 | DCAR_014488 |
| 1877 | DCAR_014499 |
| 1878 | DCAR_014505 |
| 1879 | DCAR_014507 |
| 1880 | DCAR_014517 |
| 1881 | DCAR_014520 |
| 1882 | DCAR_014521 |
| 1883 | DCAR_014549 |
| 1884 | DCAR_014553 |
| 1885 | DCAR_014567 |
| 1886 | DCAR_014570 |
| 1887 | DCAR_014575 |
| 1888 | DCAR_014635 |
| 1889 | DCAR_014646 |
| 1890 | DCAR_014656 |
| 1891 | DCAR_014658 |
| 1892 | DCAR_014677 |
| 1893 | DCAR_014690 |
| 1894 | DCAR_014692 |
| 1895 | DCAR_014711 |
| 1896 | DCAR_014714 |
| 1897 | DCAR_014726 |
| 1898 | DCAR_014737 |
| 1899 | DCAR_014770 |
| 1900 | DCAR_014775 |
| 1901 | DCAR_014789 |
| 1902 | DCAR_014804 |
| 1903 | DCAR_014808 |
| 1904 | DCAR_014811 |
| 1905 | DCAR_014827 |
| 1906 | DCAR_014828 |
| 1907 | DCAR_014833 |
| 1908 | DCAR_014843 |
| 1909 | DCAR_014848 |
| 1910 | DCAR_014874 |
| 1911 | DCAR_014883 |
| 1912 | DCAR_014885 |
| 1913 | DCAR_014909 |
| 1914 | DCAR_014922 |
| 1915 | DCAR_014959 |
| 1916 | DCAR_014960 |
| 1917 | DCAR_014963 |
| 1918 | DCAR_014965 |
| 1919 | DCAR_014981 |
| 1920 | DCAR_014982 |
| 1921 | DCAR_014993 |
| 1922 | DCAR_015001 |
| 1923 | DCAR_015004 |
| 1924 | DCAR_015012 |
| 1925 | DCAR_015016 |
| 1926 | DCAR_015018 |
| 1927 | DCAR_015026 |
| 1928 | DCAR_015052 |
| 1929 | DCAR_015053 |
| 1930 | DCAR_015054 |
| 1931 | DCAR_015067 |
| 1932 | DCAR_015085 |
| 1933 | DCAR_015103 |
| 1934 | DCAR_015123 |
| 1935 | DCAR_015133 |
| 1936 | DCAR_015154 |
| 1937 | DCAR_015168 |
| 1938 | DCAR_015173 |
| 1939 | DCAR_015188 |
| 1940 | DCAR_015201 |
| 1941 | DCAR_015210 |
| 1942 | DCAR_015215 |
| 1943 | DCAR_015228 |
| 1944 | DCAR_015231 |
| 1945 | DCAR_015235 |
| 1946 | DCAR_015240 |
| 1947 | DCAR_015242 |
| 1948 | DCAR_015247 |
| 1949 | DCAR_015250 |
| 1950 | DCAR_015251 |
| 1951 | DCAR_015258 |
| 1952 | DCAR_015262 |
| 1953 | DCAR_015263 |
| 1954 | DCAR_015268 |
| 1955 | DCAR_015270 |
| 1956 | DCAR_015292 |
| 1957 | DCAR_015309 |
| 1958 | DCAR_015311 |
| 1959 | DCAR_015315 |
| 1960 | DCAR_015319 |
| 1961 | DCAR_015322 |
| 1962 | DCAR_015325 |
| 1963 | DCAR_015328 |
| 1964 | DCAR_015336 |
| 1965 | DCAR_015337 |
| 1966 | DCAR_015339 |
| 1967 | DCAR_015352 |
| 1968 | DCAR_015381 |
| 1969 | DCAR_015387 |
| 1970 | DCAR_015393 |
| 1971 | DCAR_015400 |
| 1972 | DCAR_015407 |
| 1973 | DCAR_015417 |
| 1974 | DCAR_015460 |
| 1975 | DCAR_015482 |
| 1976 | DCAR_015485 |
| 1977 | DCAR_015486 |
| 1978 | DCAR_015506 |
| 1979 | DCAR_015532 |
| 1980 | DCAR_015545 |
| 1981 | DCAR_015549 |
| 1982 | DCAR_015560 |
| 1983 | DCAR_015577 |
| 1984 | DCAR_015583 |
| 1985 | DCAR_015585 |
| 1986 | DCAR_015594 |
| 1987 | DCAR_015598 |
| 1988 | DCAR_015614 |
| 1989 | DCAR_015615 |
| 1990 | DCAR_015624 |
| 1991 | DCAR_015627 |
| 1992 | DCAR_015633 |
| 1993 | DCAR_015634 |
| 1994 | DCAR_015638 |
| 1995 | DCAR_015643 |
| 1996 | DCAR_015644 |
| 1997 | DCAR_015651 |
| 1998 | DCAR_015664 |
| 1999 | DCAR_015665 |
| 2000 | DCAR_015666 |
| 2001 | DCAR_015668 |
| 2002 | DCAR_015669 |
| 2003 | DCAR_015673 |
| 2004 | DCAR_015674 |
| 2005 | DCAR_015675 |
| 2006 | DCAR_015684 |
| 2007 | DCAR_015693 |
| 2008 | DCAR_015711 |
| 2009 | DCAR_015724 |
| 2010 | DCAR_015753 |
| 2011 | DCAR_015756 |
| 2012 | DCAR_015765 |
| 2013 | DCAR_015774 |
| 2014 | DCAR_015775 |
| 2015 | DCAR_015776 |
| 2016 | DCAR_015785 |
| 2017 | DCAR_015787 |
| 2018 | DCAR_015790 |
| 2019 | DCAR_015863 |
| 2020 | DCAR_015867 |
| 2021 | DCAR_015889 |
| 2022 | DCAR_015896 |
| 2023 | DCAR_015928 |
| 2024 | DCAR_015934 |
| 2025 | DCAR_015936 |
| 2026 | DCAR_015950 |
| 2027 | DCAR_015968 |
| 2028 | DCAR_015997 |
| 2029 | DCAR_016001 |
| 2030 | DCAR_016032 |
| 2031 | DCAR_016049 |
| 2032 | DCAR_016084 |
| 2033 | DCAR_016086 |
| 2034 | DCAR_016089 |
| 2035 | DCAR_016090 |
| 2036 | DCAR_016091 |
| 2037 | DCAR_016092 |
| 2038 | DCAR_016106 |
| 2039 | DCAR_016131 |
| 2040 | DCAR_016134 |
| 2041 | DCAR_016139 |
| 2042 | DCAR_016150 |
| 2043 | DCAR_016151 |
| 2044 | DCAR_016153 |
| 2045 | DCAR_016159 |
| 2046 | DCAR_016162 |
| 2047 | DCAR_016163 |
| 2048 | DCAR_016164 |
| 2049 | DCAR_016181 |
| 2050 | DCAR_016190 |
| 2051 | DCAR_016198 |
| 2052 | DCAR_016201 |
| 2053 | DCAR_016206 |
| 2054 | DCAR_016209 |
| 2055 | DCAR_016211 |
| 2056 | DCAR_016217 |
| 2057 | DCAR_016223 |
| 2058 | DCAR_016229 |
| 2059 | DCAR_016232 |
| 2060 | DCAR_016237 |
| 2061 | DCAR_016239 |
| 2062 | DCAR_016249 |
| 2063 | DCAR_016258 |
| 2064 | DCAR_016275 |
| 2065 | DCAR_016277 |
| 2066 | DCAR_016286 |
| 2067 | DCAR_016291 |
| 2068 | DCAR_016293 |
| 2069 | DCAR_016298 |
| 2070 | DCAR_016309 |
| 2071 | DCAR_016310 |
| 2072 | DCAR_016320 |
| 2073 | DCAR_016324 |
| 2074 | DCAR_016330 |
| 2075 | DCAR_016335 |
| 2076 | DCAR_016350 |
| 2077 | DCAR_016352 |
| 2078 | DCAR_016354 |
| 2079 | DCAR_016355 |
| 2080 | DCAR_016361 |
| 2081 | DCAR_016362 |
| 2082 | DCAR_016373 |
| 2083 | DCAR_016383 |
| 2084 | DCAR_016384 |
| 2085 | DCAR_016387 |
| 2086 | DCAR_016403 |
| 2087 | DCAR_016404 |
| 2088 | DCAR_016408 |
| 2089 | DCAR_016414 |
| 2090 | DCAR_016419 |
| 2091 | DCAR_016420 |
| 2092 | DCAR_016425 |
| 2093 | DCAR_016433 |
| 2094 | DCAR_016458 |
| 2095 | DCAR_016486 |
| 2096 | DCAR_016501 |
| 2097 | DCAR_016522 |
| 2098 | DCAR_016531 |
| 2099 | DCAR_016537 |
| 2100 | DCAR_016549 |
| 2101 | DCAR_016565 |
| 2102 | DCAR_016569 |
| 2103 | DCAR_016573 |
| 2104 | DCAR_016594 |
| 2105 | DCAR_016595 |
| 2106 | DCAR_016600 |
| 2107 | DCAR_016603 |
| 2108 | DCAR_016619 |
| 2109 | DCAR_016646 |
| 2110 | DCAR_016648 |
| 2111 | DCAR_016660 |
| 2112 | DCAR_016674 |
| 2113 | DCAR_016682 |
| 2114 | DCAR_016684 |
| 2115 | DCAR_016686 |
| 2116 | DCAR_016687 |
| 2117 | DCAR_016689 |
| 2118 | DCAR_016693 |
| 2119 | DCAR_016698 |
| 2120 | DCAR_016705 |
| 2121 | DCAR_016708 |
| 2122 | DCAR_016716 |
| 2123 | DCAR_016724 |
| 2124 | DCAR_016729 |
| 2125 | DCAR_016740 |
| 2126 | DCAR_016750 |
| 2127 | DCAR_016767 |
| 2128 | DCAR_016785 |
| 2129 | DCAR_016794 |
| 2130 | DCAR_016815 |
| 2131 | DCAR_016836 |
| 2132 | DCAR_016837 |
| 2133 | DCAR_016838 |
| 2134 | DCAR_016845 |
| 2135 | DCAR_016846 |
| 2136 | DCAR_016855 |
| 2137 | DCAR_016868 |
| 2138 | DCAR_016892 |
| 2139 | DCAR_016904 |
| 2140 | DCAR_016909 |
| 2141 | DCAR_016918 |
| 2142 | DCAR_016922 |
| 2143 | DCAR_016935 |
| 2144 | DCAR_016944 |
| 2145 | DCAR_016962 |
| 2146 | DCAR_016976 |
| 2147 | DCAR_016999 |
| 2148 | DCAR_017036 |
| 2149 | DCAR_017037 |
| 2150 | DCAR_017048 |
| 2151 | DCAR_017053 |
| 2152 | DCAR_017062 |
| 2153 | DCAR_017063 |
| 2154 | DCAR_017065 |
| 2155 | DCAR_017070 |
| 2156 | DCAR_017072 |
| 2157 | DCAR_017074 |
| 2158 | DCAR_017075 |
| 2159 | DCAR_017085 |
| 2160 | DCAR_017089 |
| 2161 | DCAR_017094 |
| 2162 | DCAR_017102 |
| 2163 | DCAR_017106 |
| 2164 | DCAR_017107 |
| 2165 | DCAR_017109 |
| 2166 | DCAR_017167 |
| 2167 | DCAR_017203 |
| 2168 | DCAR_017219 |
| 2169 | DCAR_017221 |
| 2170 | DCAR_017225 |
| 2171 | DCAR_017270 |
| 2172 | DCAR_017291 |
| 2173 | DCAR_017304 |
| 2174 | DCAR_017308 |
| 2175 | DCAR_017316 |
| 2176 | DCAR_017317 |
| 2177 | DCAR_017335 |
| 2178 | DCAR_017347 |
| 2179 | DCAR_017348 |
| 2180 | DCAR_017393 |
| 2181 | DCAR_017408 |
| 2182 | DCAR_017442 |
| 2183 | DCAR_017497 |
| 2184 | DCAR_017510 |
| 2185 | DCAR_017559 |
| 2186 | DCAR_017573 |
| 2187 | DCAR_017575 |
| 2188 | DCAR_017585 |
| 2189 | DCAR_017590 |
| 2190 | DCAR_017596 |
| 2191 | DCAR_017601 |
| 2192 | DCAR_017606 |
| 2193 | DCAR_017644 |
| 2194 | DCAR_017645 |
| 2195 | DCAR_017649 |
| 2196 | DCAR_017678 |
| 2197 | DCAR_017682 |
| 2198 | DCAR_017688 |
| 2199 | DCAR_017696 |
| 2200 | DCAR_017712 |
| 2201 | DCAR_017719 |
| 2202 | DCAR_017723 |
| 2203 | DCAR_017744 |
| 2204 | DCAR_017752 |
| 2205 | DCAR_017765 |
| 2206 | DCAR_017775 |
| 2207 | DCAR_017776 |
| 2208 | DCAR_017778 |
| 2209 | DCAR_017793 |
| 2210 | DCAR_017796 |
| 2211 | DCAR_017813 |
| 2212 | DCAR_017815 |
| 2213 | DCAR_017816 |
| 2214 | DCAR_017826 |
| 2215 | DCAR_017828 |
| 2216 | DCAR_017867 |
| 2217 | DCAR_017885 |
| 2218 | DCAR_017901 |
| 2219 | DCAR_017902 |
| 2220 | DCAR_017909 |
| 2221 | DCAR_017910 |
| 2222 | DCAR_017911 |
| 2223 | DCAR_017912 |
| 2224 | DCAR_017916 |
| 2225 | DCAR_017938 |
| 2226 | DCAR_017955 |
| 2227 | DCAR_017956 |
| 2228 | DCAR_017957 |
| 2229 | DCAR_017966 |
| 2230 | DCAR_017968 |
| 2231 | DCAR_017973 |
| 2232 | DCAR_017981 |
| 2233 | DCAR_018029 |
| 2234 | DCAR_018047 |
| 2235 | DCAR_018048 |
| 2236 | DCAR_018109 |
| 2237 | DCAR_018131 |
| 2238 | DCAR_018138 |
| 2239 | DCAR_018140 |
| 2240 | DCAR_018141 |
| 2241 | DCAR_018153 |
| 2242 | DCAR_018154 |
| 2243 | DCAR_018183 |
| 2244 | DCAR_018200 |
| 2245 | DCAR_018201 |
| 2246 | DCAR_018205 |
| 2247 | DCAR_018210 |
| 2248 | DCAR_018216 |
| 2249 | DCAR_018228 |
| 2250 | DCAR_018231 |
| 2251 | DCAR_018237 |
| 2252 | DCAR_018248 |
| 2253 | DCAR_018255 |
| 2254 | DCAR_018280 |
| 2255 | DCAR_018298 |
| 2256 | DCAR_018310 |
| 2257 | DCAR_018320 |
| 2258 | DCAR_018321 |
| 2259 | DCAR_018324 |
| 2260 | DCAR_018325 |
| 2261 | DCAR_018340 |
| 2262 | DCAR_018366 |
| 2263 | DCAR_018380 |
| 2264 | DCAR_018381 |
| 2265 | DCAR_018402 |
| 2266 | DCAR_018412 |
| 2267 | DCAR_018419 |
| 2268 | DCAR_018422 |
| 2269 | DCAR_018425 |
| 2270 | DCAR_018431 |
| 2271 | DCAR_018445 |
| 2272 | DCAR_018446 |
| 2273 | DCAR_018489 |
| 2274 | DCAR_018494 |
| 2275 | DCAR_018500 |
| 2276 | DCAR_018503 |
| 2277 | DCAR_018505 |
| 2278 | DCAR_018521 |
| 2279 | DCAR_018545 |
| 2280 | DCAR_018554 |
| 2281 | DCAR_018555 |
| 2282 | DCAR_018578 |
| 2283 | DCAR_018580 |
| 2284 | DCAR_018581 |
| 2285 | DCAR_018582 |
| 2286 | DCAR_018592 |
| 2287 | DCAR_018595 |
| 2288 | DCAR_018596 |
| 2289 | DCAR_018600 |
| 2290 | DCAR_018606 |
| 2291 | DCAR_018610 |
| 2292 | DCAR_018617 |
| 2293 | DCAR_018637 |
| 2294 | DCAR_018674 |
| 2295 | DCAR_018682 |
| 2296 | DCAR_018684 |
| 2297 | DCAR_018690 |
| 2298 | DCAR_018713 |
| 2299 | DCAR_018735 |
| 2300 | DCAR_018739 |
| 2301 | DCAR_018740 |
| 2302 | DCAR_018747 |
| 2303 | DCAR_018748 |
| 2304 | DCAR_018749 |
| 2305 | DCAR_018750 |
| 2306 | DCAR_018754 |
| 2307 | DCAR_018757 |
| 2308 | DCAR_018790 |
| 2309 | DCAR_018791 |
| 2310 | DCAR_018813 |
| 2311 | DCAR_018833 |
| 2312 | DCAR_018834 |
| 2313 | DCAR_018836 |
| 2314 | DCAR_018839 |
| 2315 | DCAR_018843 |
| 2316 | DCAR_018873 |
| 2317 | DCAR_018900 |
| 2318 | DCAR_018930 |
| 2319 | DCAR_018937 |
| 2320 | DCAR_018939 |
| 2321 | DCAR_018957 |
| 2322 | DCAR_018958 |
| 2323 | DCAR_018962 |
| 2324 | DCAR_018967 |
| 2325 | DCAR_018977 |
| 2326 | DCAR_018978 |
| 2327 | DCAR_018985 |
| 2328 | DCAR_018991 |
| 2329 | DCAR_019007 |
| 2330 | DCAR_019012 |
| 2331 | DCAR_019035 |
| 2332 | DCAR_019042 |
| 2333 | DCAR_019061 |
| 2334 | DCAR_019070 |
| 2335 | DCAR_019079 |
| 2336 | DCAR_019085 |
| 2337 | DCAR_019086 |
| 2338 | DCAR_019089 |
| 2339 | DCAR_019092 |
| 2340 | DCAR_019097 |
| 2341 | DCAR_019122 |
| 2342 | DCAR_019129 |
| 2343 | DCAR_019132 |
| 2344 | DCAR_019149 |
| 2345 | DCAR_019162 |
| 2346 | DCAR_019164 |
| 2347 | DCAR_019167 |
| 2348 | DCAR_019192 |
| 2349 | DCAR_019198 |
| 2350 | DCAR_019212 |
| 2351 | DCAR_019222 |
| 2352 | DCAR_019227 |
| 2353 | DCAR_019228 |
| 2354 | DCAR_019245 |
| 2355 | DCAR_019248 |
| 2356 | DCAR_019257 |
| 2357 | DCAR_019268 |
| 2358 | DCAR_019274 |
| 2359 | DCAR_019284 |
| 2360 | DCAR_019288 |
| 2361 | DCAR_019292 |
| 2362 | DCAR_019300 |
| 2363 | DCAR_019305 |
| 2364 | DCAR_019334 |
| 2365 | DCAR_019337 |
| 2366 | DCAR_019349 |
| 2367 | DCAR_019380 |
| 2368 | DCAR_019381 |
| 2369 | DCAR_019404 |
| 2370 | DCAR_019406 |
| 2371 | DCAR_019420 |
| 2372 | DCAR_019429 |
| 2373 | DCAR_019448 |
| 2374 | DCAR_019453 |
| 2375 | DCAR_019466 |
| 2376 | DCAR_019495 |
| 2377 | DCAR_019496 |
| 2378 | DCAR_019515 |
| 2379 | DCAR_019523 |
| 2380 | DCAR_019524 |
| 2381 | DCAR_019525 |
| 2382 | DCAR_019530 |
| 2383 | DCAR_019532 |
| 2384 | DCAR_019533 |
| 2385 | DCAR_019544 |
| 2386 | DCAR_019548 |
| 2387 | DCAR_019550 |
| 2388 | DCAR_019570 |
| 2389 | DCAR_019573 |
| 2390 | DCAR_019584 |
| 2391 | DCAR_019588 |
| 2392 | DCAR_019590 |
| 2393 | DCAR_019594 |
| 2394 | DCAR_019596 |
| 2395 | DCAR_019610 |
| 2396 | DCAR_019628 |
| 2397 | DCAR_019664 |
| 2398 | DCAR_019667 |
| 2399 | DCAR_019670 |
| 2400 | DCAR_019671 |
| 2401 | DCAR_019673 |
| 2402 | DCAR_019674 |
| 2403 | DCAR_019677 |
| 2404 | DCAR_019707 |
| 2405 | DCAR_019735 |
| 2406 | DCAR_019739 |
| 2407 | DCAR_019745 |
| 2408 | DCAR_019750 |
| 2409 | DCAR_019755 |
| 2410 | DCAR_019766 |
| 2411 | DCAR_019767 |
| 2412 | DCAR_019784 |
| 2413 | DCAR_019786 |
| 2414 | DCAR_019791 |
| 2415 | DCAR_019794 |
| 2416 | DCAR_019795 |
| 2417 | DCAR_019801 |
| 2418 | DCAR_019818 |
| 2419 | DCAR_019829 |
| 2420 | DCAR_019837 |
| 2421 | DCAR_019844 |
| 2422 | DCAR_019851 |
| 2423 | DCAR_019857 |
| 2424 | DCAR_019878 |
| 2425 | DCAR_019879 |
| 2426 | DCAR_019884 |
| 2427 | DCAR_019889 |
| 2428 | DCAR_019902 |
| 2429 | DCAR_019904 |
| 2430 | DCAR_019913 |
| 2431 | DCAR_019918 |
| 2432 | DCAR_019919 |
| 2433 | DCAR_019926 |
| 2434 | DCAR_019928 |
| 2435 | DCAR_019937 |
| 2436 | DCAR_019957 |
| 2437 | DCAR_019964 |
| 2438 | DCAR_019970 |
| 2439 | DCAR_019987 |
| 2440 | DCAR_019990 |
| 2441 | DCAR_020023 |
| 2442 | DCAR_020028 |
| 2443 | DCAR_020034 |
| 2444 | DCAR_020038 |
| 2445 | DCAR_020052 |
| 2446 | DCAR_020079 |
| 2447 | DCAR_020086 |
| 2448 | DCAR_020089 |
| 2449 | DCAR_020092 |
| 2450 | DCAR_020108 |
| 2451 | DCAR_020121 |
| 2452 | DCAR_020124 |
| 2453 | DCAR_020129 |
| 2454 | DCAR_020136 |
| 2455 | DCAR_020144 |
| 2456 | DCAR_020157 |
| 2457 | DCAR_020164 |
| 2458 | DCAR_020176 |
| 2459 | DCAR_020192 |
| 2460 | DCAR_020201 |
| 2461 | DCAR_020203 |
| 2462 | DCAR_020206 |
| 2463 | DCAR_020209 |
| 2464 | DCAR_020225 |
| 2465 | DCAR_020230 |
| 2466 | DCAR_020232 |
| 2467 | DCAR_020237 |
| 2468 | DCAR_020241 |
| 2469 | DCAR_020243 |
| 2470 | DCAR_020256 |
| 2471 | DCAR_020264 |
| 2472 | DCAR_020272 |
| 2473 | DCAR_020274 |
| 2474 | DCAR_020276 |
| 2475 | DCAR_020288 |
| 2476 | DCAR_020294 |
| 2477 | DCAR_020297 |
| 2478 | DCAR_020320 |
| 2479 | DCAR_020325 |
| 2480 | DCAR_020337 |
| 2481 | DCAR_020339 |
| 2482 | DCAR_020349 |
| 2483 | DCAR_020352 |
| 2484 | DCAR_020361 |
| 2485 | DCAR_020392 |
| 2486 | DCAR_020396 |
| 2487 | DCAR_020398 |
| 2488 | DCAR_020404 |
| 2489 | DCAR_020432 |
| 2490 | DCAR_020436 |
| 2491 | DCAR_020446 |
| 2492 | DCAR_020447 |
| 2493 | DCAR_020450 |
| 2494 | DCAR_020451 |
| 2495 | DCAR_020453 |
| 2496 | DCAR_020455 |
| 2497 | DCAR_020460 |
| 2498 | DCAR_020479 |
| 2499 | DCAR_020490 |
| 2500 | DCAR_020493 |
| 2501 | DCAR_020497 |
| 2502 | DCAR_020512 |
| 2503 | DCAR_020525 |
| 2504 | DCAR_020526 |
| 2505 | DCAR_020543 |
| 2506 | DCAR_020564 |
| 2507 | DCAR_020565 |
| 2508 | DCAR_020575 |
| 2509 | DCAR_020581 |
| 2510 | DCAR_020582 |
| 2511 | DCAR_020584 |
| 2512 | DCAR_020586 |
| 2513 | DCAR_020587 |
| 2514 | DCAR_020601 |
| 2515 | DCAR_020604 |
| 2516 | DCAR_020609 |
| 2517 | DCAR_020614 |
| 2518 | DCAR_020626 |
| 2519 | DCAR_020627 |
| 2520 | DCAR_020628 |
| 2521 | DCAR_020631 |
| 2522 | DCAR_020633 |
| 2523 | DCAR_020636 |
| 2524 | DCAR_020640 |
| 2525 | DCAR_020655 |
| 2526 | DCAR_020659 |
| 2527 | DCAR_020674 |
| 2528 | DCAR_020688 |
| 2529 | DCAR_020694 |
| 2530 | DCAR_020703 |
| 2531 | DCAR_020705 |
| 2532 | DCAR_020709 |
| 2533 | DCAR_020711 |
| 2534 | DCAR_020723 |
| 2535 | DCAR_020736 |
| 2536 | DCAR_020740 |
| 2537 | DCAR_020752 |
| 2538 | DCAR_020762 |
| 2539 | DCAR_020770 |
| 2540 | DCAR_020776 |
| 2541 | DCAR_020777 |
| 2542 | DCAR_020781 |
| 2543 | DCAR_020782 |
| 2544 | DCAR_020795 |
| 2545 | DCAR_020802 |
| 2546 | DCAR_020805 |
| 2547 | DCAR_020806 |
| 2548 | DCAR_020814 |
| 2549 | DCAR_020819 |
| 2550 | DCAR_020822 |
| 2551 | DCAR_020842 |
| 2552 | DCAR_020846 |
| 2553 | DCAR_020847 |
| 2554 | DCAR_020853 |
| 2555 | DCAR_020857 |
| 2556 | DCAR_020864 |
| 2557 | DCAR_020871 |
| 2558 | DCAR_020875 |
| 2559 | DCAR_020886 |
| 2560 | DCAR_020920 |
| 2561 | DCAR_020922 |
| 2562 | DCAR_020929 |
| 2563 | DCAR_020940 |
| 2564 | DCAR_020943 |
| 2565 | DCAR_020951 |
| 2566 | DCAR_020963 |
| 2567 | DCAR_020967 |
| 2568 | DCAR_020970 |
| 2569 | DCAR_020971 |
| 2570 | DCAR_020972 |
| 2571 | DCAR_020983 |
| 2572 | DCAR_020992 |
| 2573 | DCAR_020994 |
| 2574 | DCAR_021003 |
| 2575 | DCAR_021015 |
| 2576 | DCAR_021021 |
| 2577 | DCAR_021022 |
| 2578 | DCAR_021025 |
| 2579 | DCAR_021035 |
| 2580 | DCAR_021036 |
| 2581 | DCAR_021047 |
| 2582 | DCAR_021051 |
| 2583 | DCAR_021060 |
| 2584 | DCAR_021075 |
| 2585 | DCAR_021084 |
| 2586 | DCAR_021089 |
| 2587 | DCAR_021093 |
| 2588 | DCAR_021102 |
| 2589 | DCAR_021104 |
| 2590 | DCAR_021109 |
| 2591 | DCAR_021112 |
| 2592 | DCAR_021148 |
| 2593 | DCAR_021170 |
| 2594 | DCAR_021194 |
| 2595 | DCAR_021214 |
| 2596 | DCAR_021233 |
| 2597 | DCAR_021236 |
| 2598 | DCAR_021237 |
| 2599 | DCAR_021238 |
| 2600 | DCAR_021249 |
| 2601 | DCAR_021262 |
| 2602 | DCAR_021270 |
| 2603 | DCAR_021289 |
| 2604 | DCAR_021291 |
| 2605 | DCAR_021296 |
| 2606 | DCAR_021300 |
| 2607 | DCAR_021314 |
| 2608 | DCAR_021318 |
| 2609 | DCAR_021320 |
| 2610 | DCAR_021325 |
| 2611 | DCAR_021328 |
| 2612 | DCAR_021349 |
| 2613 | DCAR_021356 |
| 2614 | DCAR_021358 |
| 2615 | DCAR_021360 |
| 2616 | DCAR_021370 |
| 2617 | DCAR_021383 |
| 2618 | DCAR_021386 |
| 2619 | DCAR_021388 |
| 2620 | DCAR_021389 |
| 2621 | DCAR_021395 |
| 2622 | DCAR_021420 |
| 2623 | DCAR_021423 |
| 2624 | DCAR_021424 |
| 2625 | DCAR_021426 |
| 2626 | DCAR_021441 |
| 2627 | DCAR_021442 |
| 2628 | DCAR_021447 |
| 2629 | DCAR_021459 |
| 2630 | DCAR_021470 |
| 2631 | DCAR_021491 |
| 2632 | DCAR_021495 |
| 2633 | DCAR_021505 |
| 2634 | DCAR_021510 |
| 2635 | DCAR_021518 |
| 2636 | DCAR_021527 |
| 2637 | DCAR_021532 |
| 2638 | DCAR_021533 |
| 2639 | DCAR_021534 |
| 2640 | DCAR_021540 |
| 2641 | DCAR_021564 |
| 2642 | DCAR_021568 |
| 2643 | DCAR_021583 |
| 2644 | DCAR_021601 |
| 2645 | DCAR_021603 |
| 2646 | DCAR_021604 |
| 2647 | DCAR_021612 |
| 2648 | DCAR_021625 |
| 2649 | DCAR_021646 |
| 2650 | DCAR_021651 |
| 2651 | DCAR_021663 |
| 2652 | DCAR_021682 |
| 2653 | DCAR_021687 |
| 2654 | DCAR_021694 |
| 2655 | DCAR_021704 |
| 2656 | DCAR_021708 |
| 2657 | DCAR_021732 |
| 2658 | DCAR_021742 |
| 2659 | DCAR_021749 |
| 2660 | DCAR_021753 |
| 2661 | DCAR_021759 |
| 2662 | DCAR_021762 |
| 2663 | DCAR_021764 |
| 2664 | DCAR_021768 |
| 2665 | DCAR_021771 |
| 2666 | DCAR_021791 |
| 2667 | DCAR_021796 |
| 2668 | DCAR_021803 |
| 2669 | DCAR_021810 |
| 2670 | DCAR_021818 |
| 2671 | DCAR_021835 |
| 2672 | DCAR_021841 |
| 2673 | DCAR_021847 |
| 2674 | DCAR_021850 |
| 2675 | DCAR_021861 |
| 2676 | DCAR_021865 |
| 2677 | DCAR_021873 |
| 2678 | DCAR_021876 |
| 2679 | DCAR_021878 |
| 2680 | DCAR_021887 |
| 2681 | DCAR_021901 |
| 2682 | DCAR_021904 |
| 2683 | DCAR_021911 |
| 2684 | DCAR_021917 |
| 2685 | DCAR_021918 |
| 2686 | DCAR_021923 |
| 2687 | DCAR_021938 |
| 2688 | DCAR_021954 |
| 2689 | DCAR_021968 |
| 2690 | DCAR_021982 |
| 2691 | DCAR_021994 |
| 2692 | DCAR_021998 |
| 2693 | DCAR_022001 |
| 2694 | DCAR_022007 |
| 2695 | DCAR_022067 |
| 2696 | DCAR_022068 |
| 2697 | DCAR_022087 |
| 2698 | DCAR_022090 |
| 2699 | DCAR_022097 |
| 2700 | DCAR_022102 |
| 2701 | DCAR_022104 |
| 2702 | DCAR_022114 |
| 2703 | DCAR_022116 |
| 2704 | DCAR_022117 |
| 2705 | DCAR_022124 |
| 2706 | DCAR_022129 |
| 2707 | DCAR_022132 |
| 2708 | DCAR_022148 |
| 2709 | DCAR_022174 |
| 2710 | DCAR_022176 |
| 2711 | DCAR_022193 |
| 2712 | DCAR_022254 |
| 2713 | DCAR_022274 |
| 2714 | DCAR_022280 |
| 2715 | DCAR_022281 |
| 2716 | DCAR_022334 |
| 2717 | DCAR_022337 |
| 2718 | DCAR_022339 |
| 2719 | DCAR_022344 |
| 2720 | DCAR_022349 |
| 2721 | DCAR_022370 |
| 2722 | DCAR_022380 |
| 2723 | DCAR_022384 |
| 2724 | DCAR_022409 |
| 2725 | DCAR_022418 |
| 2726 | DCAR_022423 |
| 2727 | DCAR_022438 |
| 2728 | DCAR_022439 |
| 2729 | DCAR_022445 |
| 2730 | DCAR_022446 |
| 2731 | DCAR_022470 |
| 2732 | DCAR_022482 |
| 2733 | DCAR_022491 |
| 2734 | DCAR_022496 |
| 2735 | DCAR_022497 |
| 2736 | DCAR_022498 |
| 2737 | DCAR_022517 |
| 2738 | DCAR_022531 |
| 2739 | DCAR_022551 |
| 2740 | DCAR_022552 |
| 2741 | DCAR_022555 |
| 2742 | DCAR_022563 |
| 2743 | DCAR_022565 |
| 2744 | DCAR_022577 |
| 2745 | DCAR_022578 |
| 2746 | DCAR_022586 |
| 2747 | DCAR_022595 |
| 2748 | DCAR_022599 |
| 2749 | DCAR_022600 |
| 2750 | DCAR_022602 |
| 2751 | DCAR_022610 |
| 2752 | DCAR_022614 |
| 2753 | DCAR_022617 |
| 2754 | DCAR_022627 |
| 2755 | DCAR_022630 |
| 2756 | DCAR_022648 |
| 2757 | DCAR_022649 |
| 2758 | DCAR_022650 |
| 2759 | DCAR_022662 |
| 2760 | DCAR_022669 |
| 2761 | DCAR_022673 |
| 2762 | DCAR_022675 |
| 2763 | DCAR_022680 |
| 2764 | DCAR_022687 |
| 2765 | DCAR_022688 |
| 2766 | DCAR_022700 |
| 2767 | DCAR_022703 |
| 2768 | DCAR_022712 |
| 2769 | DCAR_022768 |
| 2770 | DCAR_022772 |
| 2771 | DCAR_022775 |
| 2772 | DCAR_022789 |
| 2773 | DCAR_022816 |
| 2774 | DCAR_022817 |
| 2775 | DCAR_022835 |
| 2776 | DCAR_022837 |
| 2777 | DCAR_022865 |
| 2778 | DCAR_022868 |
| 2779 | DCAR_022872 |
| 2780 | DCAR_022902 |
| 2781 | DCAR_022905 |
| 2782 | DCAR_022907 |
| 2783 | DCAR_022909 |
| 2784 | DCAR_022919 |
| 2785 | DCAR_022921 |
| 2786 | DCAR_022924 |
| 2787 | DCAR_022931 |
| 2788 | DCAR_022933 |
| 2789 | DCAR_022934 |
| 2790 | DCAR_022937 |
| 2791 | DCAR_022947 |
| 2792 | DCAR_022950 |
| 2793 | DCAR_022952 |
| 2794 | DCAR_022955 |
| 2795 | DCAR_022959 |
| 2796 | DCAR_022960 |
| 2797 | DCAR_022966 |
| 2798 | DCAR_022973 |
| 2799 | DCAR_022989 |
| 2800 | DCAR_022994 |
| 2801 | DCAR_023005 |
| 2802 | DCAR_023011 |
| 2803 | DCAR_023014 |
| 2804 | DCAR_023032 |
| 2805 | DCAR_023040 |
| 2806 | DCAR_023061 |
| 2807 | DCAR_023095 |
| 2808 | DCAR_023123 |
| 2809 | DCAR_023135 |
| 2810 | DCAR_023207 |
| 2811 | DCAR_023224 |
| 2812 | DCAR_023226 |
| 2813 | DCAR_023227 |
| 2814 | DCAR_023228 |
| 2815 | DCAR_023233 |
| 2816 | DCAR_023280 |
| 2817 | DCAR_023283 |
| 2818 | DCAR_023287 |
| 2819 | DCAR_023289 |
| 2820 | DCAR_023296 |
| 2821 | DCAR_023301 |
| 2822 | DCAR_023327 |
| 2823 | DCAR_023329 |
| 2824 | DCAR_023335 |
| 2825 | DCAR_023336 |
| 2826 | DCAR_023337 |
| 2827 | DCAR_023341 |
| 2828 | DCAR_023347 |
| 2829 | DCAR_023367 |
| 2830 | DCAR_023375 |
| 2831 | DCAR_023377 |
| 2832 | DCAR_023398 |
| 2833 | DCAR_023402 |
| 2834 | DCAR_023413 |
| 2835 | DCAR_023441 |
| 2836 | DCAR_023442 |
| 2837 | DCAR_023443 |
| 2838 | DCAR_023445 |
| 2839 | DCAR_023458 |
| 2840 | DCAR_023465 |
| 2841 | DCAR_023473 |
| 2842 | DCAR_023483 |
| 2843 | DCAR_023495 |
| 2844 | DCAR_023505 |
| 2845 | DCAR_023521 |
| 2846 | DCAR_023534 |
| 2847 | DCAR_023536 |
| 2848 | DCAR_023542 |
| 2849 | DCAR_023564 |
| 2850 | DCAR_023577 |
| 2851 | DCAR_023583 |
| 2852 | DCAR_023608 |
| 2853 | DCAR_023618 |
| 2854 | DCAR_023619 |
| 2855 | DCAR_023648 |
| 2856 | DCAR_023656 |
| 2857 | DCAR_023665 |
| 2858 | DCAR_023667 |
| 2859 | DCAR_023671 |
| 2860 | DCAR_023691 |
| 2861 | DCAR_023707 |
| 2862 | DCAR_023720 |
| 2863 | DCAR_023762 |
| 2864 | DCAR_023783 |
| 2865 | DCAR_023796 |
| 2866 | DCAR_023806 |
| 2867 | DCAR_023818 |
| 2868 | DCAR_023822 |
| 2869 | DCAR_023830 |
| 2870 | DCAR_023834 |
| 2871 | DCAR_023861 |
| 2872 | DCAR_023870 |
| 2873 | DCAR_023872 |
| 2874 | DCAR_023882 |
| 2875 | DCAR_023898 |
| 2876 | DCAR_023905 |
| 2877 | DCAR_023912 |
| 2878 | DCAR_023917 |
| 2879 | DCAR_023944 |
| 2880 | DCAR_023945 |
| 2881 | DCAR_023946 |
| 2882 | DCAR_023948 |
| 2883 | DCAR_023955 |
| 2884 | DCAR_023959 |
| 2885 | DCAR_023960 |
| 2886 | DCAR_023965 |
| 2887 | DCAR_023975 |
| 2888 | DCAR_023982 |
| 2889 | DCAR_023983 |
| 2890 | DCAR_023988 |
| 2891 | DCAR_024006 |
| 2892 | DCAR_024018 |
| 2893 | DCAR_024021 |
| 2894 | DCAR_024024 |
| 2895 | DCAR_024025 |
| 2896 | DCAR_024038 |
| 2897 | DCAR_024040 |
| 2898 | DCAR_024042 |
| 2899 | DCAR_024043 |
| 2900 | DCAR_024057 |
| 2901 | DCAR_024071 |
| 2902 | DCAR_024110 |
| 2903 | DCAR_024111 |
| 2904 | DCAR_024120 |
| 2905 | DCAR_024126 |
| 2906 | DCAR_024129 |
| 2907 | DCAR_024140 |
| 2908 | DCAR_024141 |
| 2909 | DCAR_024159 |
| 2910 | DCAR_024164 |
| 2911 | DCAR_024174 |
| 2912 | DCAR_024188 |
| 2913 | DCAR_024194 |
| 2914 | DCAR_024195 |
| 2915 | DCAR_024203 |
| 2916 | DCAR_024227 |
| 2917 | DCAR_024228 |
| 2918 | DCAR_024250 |
| 2919 | DCAR_024256 |
| 2920 | DCAR_024258 |
| 2921 | DCAR_024259 |
| 2922 | DCAR_024262 |
| 2923 | DCAR_024291 |
| 2924 | DCAR_024319 |
| 2925 | DCAR_024372 |
| 2926 | DCAR_024375 |
| 2927 | DCAR_024377 |
| 2928 | DCAR_024386 |
| 2929 | DCAR_024393 |
| 2930 | DCAR_024413 |
| 2931 | DCAR_024418 |
| 2932 | DCAR_024439 |
| 2933 | DCAR_024444 |
| 2934 | DCAR_024451 |
| 2935 | DCAR_024454 |
| 2936 | DCAR_024459 |
| 2937 | DCAR_024460 |
| 2938 | DCAR_024463 |
| 2939 | DCAR_024464 |
| 2940 | DCAR_024465 |
| 2941 | DCAR_024469 |
| 2942 | DCAR_024479 |
| 2943 | DCAR_024500 |
| 2944 | DCAR_024515 |
| 2945 | DCAR_024517 |
| 2946 | DCAR_024533 |
| 2947 | DCAR_024535 |
| 2948 | DCAR_024550 |
| 2949 | DCAR_024551 |
| 2950 | DCAR_024559 |
| 2951 | DCAR_024561 |
| 2952 | DCAR_024584 |
| 2953 | DCAR_024593 |
| 2954 | DCAR_024636 |
| 2955 | DCAR_024645 |
| 2956 | DCAR_024649 |
| 2957 | DCAR_024654 |
| 2958 | DCAR_024658 |
| 2959 | DCAR_024695 |
| 2960 | DCAR_024701 |
| 2961 | DCAR_024706 |
| 2962 | DCAR_024710 |
| 2963 | DCAR_024719 |
| 2964 | DCAR_024720 |
| 2965 | DCAR_024732 |
| 2966 | DCAR_024734 |
| 2967 | DCAR_024741 |
| 2968 | DCAR_024757 |
| 2969 | DCAR_024765 |
| 2970 | DCAR_024768 |
| 2971 | DCAR_024773 |
| 2972 | DCAR_024776 |
| 2973 | DCAR_024777 |
| 2974 | DCAR_024791 |
| 2975 | DCAR_024828 |
| 2976 | DCAR_024829 |
| 2977 | DCAR_024844 |
| 2978 | DCAR_024855 |
| 2979 | DCAR_024857 |
| 2980 | DCAR_024867 |
| 2981 | DCAR_024875 |
| 2982 | DCAR_024878 |
| 2983 | DCAR_024887 |
| 2984 | DCAR_024893 |
| 2985 | DCAR_024898 |
| 2986 | DCAR_024911 |
| 2987 | DCAR_024912 |
| 2988 | DCAR_024913 |
| 2989 | DCAR_024916 |
| 2990 | DCAR_024929 |
| 2991 | DCAR_024936 |
| 2992 | DCAR_024946 |
| 2993 | DCAR_024951 |
| 2994 | DCAR_024956 |
| 2995 | DCAR_024964 |
| 2996 | DCAR_024965 |
| 2997 | DCAR_024972 |
| 2998 | DCAR_024976 |
| 2999 | DCAR_024985 |
| 3000 | DCAR_025010 |
| 3001 | DCAR_025011 |
| 3002 | DCAR_025022 |
| 3003 | DCAR_025025 |
| 3004 | DCAR_025032 |
| 3005 | DCAR_025033 |
| 3006 | DCAR_025035 |
| 3007 | DCAR_025042 |
| 3008 | DCAR_025055 |
| 3009 | DCAR_025058 |
| 3010 | DCAR_025064 |
| 3011 | DCAR_025139 |
| 3012 | DCAR_025143 |
| 3013 | DCAR_025145 |
| 3014 | DCAR_025148 |
| 3015 | DCAR_025170 |
| 3016 | DCAR_025190 |
| 3017 | DCAR_025215 |
| 3018 | DCAR_025227 |
| 3019 | DCAR_025241 |
| 3020 | DCAR_025245 |
| 3021 | DCAR_025251 |
| 3022 | DCAR_025253 |
| 3023 | DCAR_025264 |
| 3024 | DCAR_025274 |
| 3025 | DCAR_025282 |
| 3026 | DCAR_025327 |
| 3027 | DCAR_025355 |
| 3028 | DCAR_025366 |
| 3029 | DCAR_025380 |
| 3030 | DCAR_025412 |
| 3031 | DCAR_025414 |
| 3032 | DCAR_025416 |
| 3033 | DCAR_025419 |
| 3034 | DCAR_025447 |
| 3035 | DCAR_025471 |
| 3036 | DCAR_025477 |
| 3037 | DCAR_025491 |
| 3038 | DCAR_025505 |
| 3039 | DCAR_025506 |
| 3040 | DCAR_025515 |
| 3041 | DCAR_025517 |
| 3042 | DCAR_025520 |
| 3043 | DCAR_025525 |
| 3044 | DCAR_025528 |
| 3045 | DCAR_025567 |
| 3046 | DCAR_025584 |
| 3047 | DCAR_025616 |
| 3048 | DCAR_025630 |
| 3049 | DCAR_025636 |
| 3050 | DCAR_025640 |
| 3051 | DCAR_025646 |
| 3052 | DCAR_025652 |
| 3053 | DCAR_025657 |
| 3054 | DCAR_025659 |
| 3055 | DCAR_025668 |
| 3056 | DCAR_025682 |
| 3057 | DCAR_025701 |
| 3058 | DCAR_025709 |
| 3059 | DCAR_025710 |
| 3060 | DCAR_025711 |
| 3061 | DCAR_025730 |
| 3062 | DCAR_025770 |
| 3063 | DCAR_025771 |
| 3064 | DCAR_025782 |
| 3065 | DCAR_025786 |
| 3066 | DCAR_025788 |
| 3067 | DCAR_025793 |
| 3068 | DCAR_025797 |
| 3069 | DCAR_025811 |
| 3070 | DCAR_025812 |
| 3071 | DCAR_025814 |
| 3072 | DCAR_025824 |
| 3073 | DCAR_025826 |
| 3074 | DCAR_025835 |
| 3075 | DCAR_025839 |
| 3076 | DCAR_025844 |
| 3077 | DCAR_025859 |
| 3078 | DCAR_025863 |
| 3079 | DCAR_025864 |
| 3080 | DCAR_025879 |
| 3081 | DCAR_025892 |
| 3082 | DCAR_025913 |
| 3083 | DCAR_025915 |
| 3084 | DCAR_025918 |
| 3085 | DCAR_025919 |
| 3086 | DCAR_025922 |
| 3087 | DCAR_025927 |
| 3088 | DCAR_025929 |
| 3089 | DCAR_025955 |
| 3090 | DCAR_025969 |
| 3091 | DCAR_025991 |
| 3092 | DCAR_025992 |
| 3093 | DCAR_025997 |
| 3094 | DCAR_026004 |
| 3095 | DCAR_026006 |
| 3096 | DCAR_026053 |
| 3097 | DCAR_026055 |
| 3098 | DCAR_026064 |
| 3099 | DCAR_026066 |
| 3100 | DCAR_026070 |
| 3101 | DCAR_026071 |
| 3102 | DCAR_026076 |
| 3103 | DCAR_026081 |
| 3104 | DCAR_026083 |
| 3105 | DCAR_026119 |
| 3106 | DCAR_026128 |
| 3107 | DCAR_026131 |
| 3108 | DCAR_026139 |
| 3109 | DCAR_026169 |
| 3110 | DCAR_026170 |
| 3111 | DCAR_026197 |
| 3112 | DCAR_026215 |
| 3113 | DCAR_026217 |
| 3114 | DCAR_026220 |
| 3115 | DCAR_026222 |
| 3116 | DCAR_026227 |
| 3117 | DCAR_026253 |
| 3118 | DCAR_026273 |
| 3119 | DCAR_026291 |
| 3120 | DCAR_026296 |
| 3121 | DCAR_026298 |
| 3122 | DCAR_026302 |
| 3123 | DCAR_026311 |
| 3124 | DCAR_026315 |
| 3125 | DCAR_026324 |
| 3126 | DCAR_026353 |
| 3127 | DCAR_026383 |
| 3128 | DCAR_026387 |
| 3129 | DCAR_026406 |
| 3130 | DCAR_026409 |
| 3131 | DCAR_026412 |
| 3132 | DCAR_026417 |
| 3133 | DCAR_026422 |
| 3134 | DCAR_026426 |
| 3135 | DCAR_026439 |
| 3136 | DCAR_026441 |
| 3137 | DCAR_026442 |
| 3138 | DCAR_026446 |
| 3139 | DCAR_026469 |
| 3140 | DCAR_026471 |
| 3141 | DCAR_026472 |
| 3142 | DCAR_026480 |
| 3143 | DCAR_026486 |
| 3144 | DCAR_026494 |
| 3145 | DCAR_026496 |
| 3146 | DCAR_026513 |
| 3147 | DCAR_026514 |
| 3148 | DCAR_026516 |
| 3149 | DCAR_026527 |
| 3150 | DCAR_026530 |
| 3151 | DCAR_026540 |
| 3152 | DCAR_026554 |
| 3153 | DCAR_026571 |
| 3154 | DCAR_026575 |
| 3155 | DCAR_026585 |
| 3156 | DCAR_026598 |
| 3157 | DCAR_026599 |
| 3158 | DCAR_026600 |
| 3159 | DCAR_026606 |
| 3160 | DCAR_026626 |
| 3161 | DCAR_026633 |
| 3162 | DCAR_026664 |
| 3163 | DCAR_026666 |
| 3164 | DCAR_026692 |
| 3165 | DCAR_026704 |
| 3166 | DCAR_026716 |
| 3167 | DCAR_026723 |
| 3168 | DCAR_026724 |
| 3169 | DCAR_026745 |
| 3170 | DCAR_026782 |
| 3171 | DCAR_026786 |
| 3172 | DCAR_026791 |
| 3173 | DCAR_026795 |
| 3174 | DCAR_026809 |
| 3175 | DCAR_026811 |
| 3176 | DCAR_026823 |
| 3177 | DCAR_026838 |
| 3178 | DCAR_026859 |
| 3179 | DCAR_026873 |
| 3180 | DCAR_026874 |
| 3181 | DCAR_026875 |
| 3182 | DCAR_026877 |
| 3183 | DCAR_026880 |
| 3184 | DCAR_026881 |
| 3185 | DCAR_026895 |
| 3186 | DCAR_026909 |
| 3187 | DCAR_026923 |
| 3188 | DCAR_026949 |
| 3189 | DCAR_026957 |
| 3190 | DCAR_026958 |
| 3191 | DCAR_026965 |
| 3192 | DCAR_026969 |
| 3193 | DCAR_026975 |
| 3194 | DCAR_026981 |
| 3195 | DCAR_026982 |
| 3196 | DCAR_026989 |
| 3197 | DCAR_026992 |
| 3198 | DCAR_026993 |
| 3199 | DCAR_027010 |
| 3200 | DCAR_027059 |
| 3201 | DCAR_027068 |
| 3202 | DCAR_027072 |
| 3203 | DCAR_027075 |
| 3204 | DCAR_027095 |
| 3205 | DCAR_027114 |
| 3206 | DCAR_027125 |
| 3207 | DCAR_027143 |
| 3208 | DCAR_027149 |
| 3209 | DCAR_027165 |
| 3210 | DCAR_027171 |
| 3211 | DCAR_027179 |
| 3212 | DCAR_027187 |
| 3213 | DCAR_027211 |
| 3214 | DCAR_027230 |
| 3215 | DCAR_027237 |
| 3216 | DCAR_027246 |
| 3217 | DCAR_027247 |
| 3218 | DCAR_027249 |
| 3219 | DCAR_027259 |
| 3220 | DCAR_027261 |
| 3221 | DCAR_027265 |
| 3222 | DCAR_027281 |
| 3223 | DCAR_027296 |
| 3224 | DCAR_027307 |
| 3225 | DCAR_027314 |
| 3226 | DCAR_027315 |
| 3227 | DCAR_027318 |
| 3228 | DCAR_027320 |
| 3229 | DCAR_027322 |
| 3230 | DCAR_027333 |
| 3231 | DCAR_027334 |
| 3232 | DCAR_027336 |
| 3233 | DCAR_027339 |
| 3234 | DCAR_027344 |
| 3235 | DCAR_027345 |
| 3236 | DCAR_027348 |
| 3237 | DCAR_027353 |
| 3238 | DCAR_027357 |
| 3239 | DCAR_027363 |
| 3240 | DCAR_027372 |
| 3241 | DCAR_027375 |
| 3242 | DCAR_027377 |
| 3243 | DCAR_027395 |
| 3244 | DCAR_027409 |
| 3245 | DCAR_027423 |
| 3246 | DCAR_027439 |
| 3247 | DCAR_027444 |
| 3248 | DCAR_027446 |
| 3249 | DCAR_027451 |
| 3250 | DCAR_027453 |
| 3251 | DCAR_027460 |
| 3252 | DCAR_027476 |
| 3253 | DCAR_027478 |
| 3254 | DCAR_027484 |
| 3255 | DCAR_027487 |
| 3256 | DCAR_027489 |
| 3257 | DCAR_027490 |
| 3258 | DCAR_027499 |
| 3259 | DCAR_027508 |
| 3260 | DCAR_027511 |
| 3261 | DCAR_027516 |
| 3262 | DCAR_027518 |
| 3263 | DCAR_027520 |
| 3264 | DCAR_027530 |
| 3265 | DCAR_027537 |
| 3266 | DCAR_027591 |
| 3267 | DCAR_027613 |
| 3268 | DCAR_027650 |
| 3269 | DCAR_027680 |
| 3270 | DCAR_027685 |
| 3271 | DCAR_027691 |
| 3272 | DCAR_027703 |
| 3273 | DCAR_027708 |
| 3274 | DCAR_027723 |
| 3275 | DCAR_027733 |
| 3276 | DCAR_027740 |
| 3277 | DCAR_027741 |
| 3278 | DCAR_027747 |
| 3279 | DCAR_027764 |
| 3280 | DCAR_027777 |
| 3281 | DCAR_027817 |
| 3282 | DCAR_027826 |
| 3283 | DCAR_027852 |
| 3284 | DCAR_027858 |
| 3285 | DCAR_027862 |
| 3286 | DCAR_027867 |
| 3287 | DCAR_027872 |
| 3288 | DCAR_027879 |
| 3289 | DCAR_027883 |
| 3290 | DCAR_027899 |
| 3291 | DCAR_027900 |
| 3292 | DCAR_027919 |
| 3293 | DCAR_027925 |
| 3294 | DCAR_027928 |
| 3295 | DCAR_027944 |
| 3296 | DCAR_027958 |
| 3297 | DCAR_027960 |
| 3298 | DCAR_027965 |
| 3299 | DCAR_027975 |
| 3300 | DCAR_027984 |
| 3301 | DCAR_028009 |
| 3302 | DCAR_028010 |
| 3303 | DCAR_028011 |
| 3304 | DCAR_028025 |
| 3305 | DCAR_028034 |
| 3306 | DCAR_028041 |
| 3307 | DCAR_028068 |
| 3308 | DCAR_028095 |
| 3309 | DCAR_028097 |
| 3310 | DCAR_028119 |
| 3311 | DCAR_028131 |
| 3312 | DCAR_028146 |
| 3313 | DCAR_028152 |
| 3314 | DCAR_028172 |
| 3315 | DCAR_028186 |
| 3316 | DCAR_028187 |
| 3317 | DCAR_028194 |
| 3318 | DCAR_028227 |
| 3319 | DCAR_028228 |
| 3320 | DCAR_028246 |
| 3321 | DCAR_028250 |
| 3322 | DCAR_028270 |
| 3323 | DCAR_028273 |
| 3324 | DCAR_028277 |
| 3325 | DCAR_028326 |
| 3326 | DCAR_028335 |
| 3327 | DCAR_028336 |
| 3328 | DCAR_028345 |
| 3329 | DCAR_028347 |
| 3330 | DCAR_028357 |
| 3331 | DCAR_028395 |
| 3332 | DCAR_028398 |
| 3333 | DCAR_028412 |
| 3334 | DCAR_028433 |
| 3335 | DCAR_028446 |
| 3336 | DCAR_028447 |
| 3337 | DCAR_028450 |
| 3338 | DCAR_028453 |
| 3339 | DCAR_028467 |
| 3340 | DCAR_028471 |
| 3341 | DCAR_028527 |
| 3342 | DCAR_028530 |
| 3343 | DCAR_028547 |
| 3344 | DCAR_028548 |
| 3345 | DCAR_028582 |
| 3346 | DCAR_028589 |
| 3347 | DCAR_028601 |
| 3348 | DCAR_028627 |
| 3349 | DCAR_028628 |
| 3350 | DCAR_028653 |
| 3351 | DCAR_028657 |
| 3352 | DCAR_028658 |
| 3353 | DCAR_028668 |
| 3354 | DCAR_028671 |
| 3355 | DCAR_028673 |
| 3356 | DCAR_028678 |
| 3357 | DCAR_028698 |
| 3358 | DCAR_028706 |
| 3359 | DCAR_028737 |
| 3360 | DCAR_028768 |
| 3361 | DCAR_028769 |
| 3362 | DCAR_028771 |
| 3363 | DCAR_028774 |
| 3364 | DCAR_028797 |
| 3365 | DCAR_028811 |
| 3366 | DCAR_028813 |
| 3367 | DCAR_028817 |
| 3368 | DCAR_028819 |
| 3369 | DCAR_028822 |
| 3370 | DCAR_028825 |
| 3371 | DCAR_028838 |
| 3372 | DCAR_028844 |
| 3373 | DCAR_028849 |
| 3374 | DCAR_028861 |
| 3375 | DCAR_028864 |
| 3376 | DCAR_028872 |
| 3377 | DCAR_028876 |
| 3378 | DCAR_028880 |
| 3379 | DCAR_028884 |
| 3380 | DCAR_028898 |
| 3381 | DCAR_028901 |
| 3382 | DCAR_028905 |
| 3383 | DCAR_028916 |
| 3384 | DCAR_028919 |
| 3385 | DCAR_028923 |
| 3386 | DCAR_028932 |
| 3387 | DCAR_028945 |
| 3388 | DCAR_028978 |
| 3389 | DCAR_028990 |
| 3390 | DCAR_029020 |
| 3391 | DCAR_029027 |
| 3392 | DCAR_029058 |
| 3393 | DCAR_029073 |
| 3394 | DCAR_029076 |
| 3395 | DCAR_029077 |
| 3396 | DCAR_029171 |
| 3397 | DCAR_029196 |
| 3398 | DCAR_029205 |
| 3399 | DCAR_029208 |
| 3400 | DCAR_029212 |
| 3401 | DCAR_029238 |
| 3402 | DCAR_029240 |
| 3403 | DCAR_029249 |
| 3404 | DCAR_029251 |
| 3405 | DCAR_029280 |
| 3406 | DCAR_029286 |
| 3407 | DCAR_029301 |
| 3408 | DCAR_029305 |
| 3409 | DCAR_029311 |
| 3410 | DCAR_029342 |
| 3411 | DCAR_029357 |
| 3412 | DCAR_029358 |
| 3413 | DCAR_029359 |
| 3414 | DCAR_029360 |
| 3415 | DCAR_029363 |
| 3416 | DCAR_029367 |
| 3417 | DCAR_029371 |
| 3418 | DCAR_029392 |
| 3419 | DCAR_029394 |
| 3420 | DCAR_029405 |
| 3421 | DCAR_029422 |
| 3422 | DCAR_029435 |
| 3423 | DCAR_029443 |
| 3424 | DCAR_029470 |
| 3425 | DCAR_029472 |
| 3426 | DCAR_029494 |
| 3427 | DCAR_029504 |
| 3428 | DCAR_029523 |
| 3429 | DCAR_029533 |
| 3430 | DCAR_029537 |
| 3431 | DCAR_029539 |
| 3432 | DCAR_029542 |
| 3433 | DCAR_029571 |
| 3434 | DCAR_029589 |
| 3435 | DCAR_029596 |
| 3436 | DCAR_029635 |
| 3437 | DCAR_029638 |
| 3438 | DCAR_029663 |
| 3439 | DCAR_029683 |
| 3440 | DCAR_029684 |
| 3441 | DCAR_029686 |
| 3442 | DCAR_029704 |
| 3443 | DCAR_029705 |
| 3444 | DCAR_029706 |
| 3445 | DCAR_029715 |
| 3446 | DCAR_029730 |
| 3447 | DCAR_029734 |
| 3448 | DCAR_029743 |
| 3449 | DCAR_029744 |
| 3450 | DCAR_029751 |
| 3451 | DCAR_029787 |
| 3452 | DCAR_029792 |
| 3453 | DCAR_029800 |
| 3454 | DCAR_029803 |
| 3455 | DCAR_029806 |
| 3456 | DCAR_029807 |
| 3457 | DCAR_029808 |
| 3458 | DCAR_029809 |
| 3459 | DCAR_029840 |
| 3460 | DCAR_029851 |
| 3461 | DCAR_029852 |
| 3462 | DCAR_029862 |
| 3463 | DCAR_029898 |
| 3464 | DCAR_029900 |
| 3465 | DCAR_029910 |
| 3466 | DCAR_029913 |
| 3467 | DCAR_029923 |
| 3468 | DCAR_029930 |
| 3469 | DCAR_029950 |
| 3470 | DCAR_029952 |
| 3471 | DCAR_029956 |
| 3472 | DCAR_029978 |
| 3473 | DCAR_029980 |
| 3474 | DCAR_029982 |
| 3475 | DCAR_029986 |
| 3476 | DCAR_029989 |
| 3477 | DCAR_029994 |
| 3478 | DCAR_030000 |
| 3479 | DCAR_030004 |
| 3480 | DCAR_030005 |
| 3481 | DCAR_030028 |
| 3482 | DCAR_030047 |
| 3483 | DCAR_030049 |
| 3484 | DCAR_030065 |
| 3485 | DCAR_030091 |
| 3486 | DCAR_030095 |
| 3487 | DCAR_030132 |
| 3488 | DCAR_030145 |
| 3489 | DCAR_030174 |
| 3490 | DCAR_030186 |
| 3491 | DCAR_030187 |
| 3492 | DCAR_030214 |
| 3493 | DCAR_030221 |
| 3494 | DCAR_030222 |
| 3495 | DCAR_030252 |
| 3496 | DCAR_030253 |
| 3497 | DCAR_030255 |
| 3498 | DCAR_030283 |
| 3499 | DCAR_030285 |
| 3500 | DCAR_030297 |
| 3501 | DCAR_030311 |
| 3502 | DCAR_030314 |
| 3503 | DCAR_030317 |
| 3504 | DCAR_030343 |
| 3505 | DCAR_030345 |
| 3506 | DCAR_030349 |
| 3507 | DCAR_030350 |
| 3508 | DCAR_030363 |
| 3509 | DCAR_030390 |
| 3510 | DCAR_030398 |
| 3511 | DCAR_030405 |
| 3512 | DCAR_030421 |
| 3513 | DCAR_030423 |
| 3514 | DCAR_030424 |
| 3515 | DCAR_030425 |
| 3516 | DCAR_030463 |
| 3517 | DCAR_030465 |
| 3518 | DCAR_030480 |
| 3519 | DCAR_030495 |
| 3520 | DCAR_030511 |
| 3521 | DCAR_030520 |
| 3522 | DCAR_030523 |
| 3523 | DCAR_030529 |
| 3524 | DCAR_030532 |
| 3525 | DCAR_030538 |
| 3526 | DCAR_030562 |
| 3527 | DCAR_030577 |
| 3528 | DCAR_030602 |
| 3529 | DCAR_030612 |
| 3530 | DCAR_030624 |
| 3531 | DCAR_030627 |
| 3532 | DCAR_030638 |
| 3533 | DCAR_030678 |
| 3534 | DCAR_030691 |
| 3535 | DCAR_030708 |
| 3536 | DCAR_030726 |
| 3537 | DCAR_030736 |
| 3538 | DCAR_030741 |
| 3539 | DCAR_030742 |
| 3540 | DCAR_030750 |
| 3541 | DCAR_030751 |
| 3542 | DCAR_030777 |
| 3543 | DCAR_030790 |
| 3544 | DCAR_030796 |
| 3545 | DCAR_030822 |
| 3546 | DCAR_030860 |
| 3547 | DCAR_030879 |
| 3548 | DCAR_030890 |
| 3549 | DCAR_030893 |
| 3550 | DCAR_030897 |
| 3551 | DCAR_030963 |
| 3552 | DCAR_030971 |
| 3553 | DCAR_030992 |
| 3554 | DCAR_030995 |
| 3555 | DCAR_031049 |
| 3556 | DCAR_031069 |
| 3557 | DCAR_031089 |
| 3558 | DCAR_031090 |
| 3559 | DCAR_031104 |
| 3560 | DCAR_031110 |
| 3561 | DCAR_031119 |
| 3562 | DCAR_031129 |
| 3563 | DCAR_031137 |
| 3564 | DCAR_031149 |
| 3565 | DCAR_031150 |
| 3566 | DCAR_031166 |
| 3567 | DCAR_031174 |
| 3568 | DCAR_031180 |
| 3569 | DCAR_031184 |
| 3570 | DCAR_031185 |
| 3571 | DCAR_031186 |
| 3572 | DCAR_031187 |
| 3573 | DCAR_031190 |
| 3574 | DCAR_031191 |
| 3575 | DCAR_031203 |
| 3576 | DCAR_031207 |
| 3577 | DCAR_031244 |
| 3578 | DCAR_031273 |
| 3579 | DCAR_031309 |
| 3580 | DCAR_031311 |
| 3581 | DCAR_031351 |
| 3582 | DCAR_031403 |
| 3583 | DCAR_031444 |
| 3584 | DCAR_031452 |
| 3585 | DCAR_031490 |
| 3586 | DCAR_031497 |
| 3587 | DCAR_031498 |
| 3588 | DCAR_031519 |
| 3589 | DCAR_031522 |
| 3590 | DCAR_031554 |
| 3591 | DCAR_031599 |
| 3592 | DCAR_031600 |
| 3593 | DCAR_031608 |
| 3594 | DCAR_031620 |
| 3595 | DCAR_031640 |
| 3596 | DCAR_031673 |
| 3597 | DCAR_031721 |
| 3598 | DCAR_031742 |
| 3599 | DCAR_031774 |
| 3600 | DCAR_031775 |
| 3601 | DCAR_031813 |
| 3602 | DCAR_031825 |
| 3603 | DCAR_031827 |
| 3604 | DCAR_031832 |
| 3605 | DCAR_031837 |
| 3606 | DCAR_031843 |
| 3607 | DCAR_031865 |
| 3608 | DCAR_031872 |
| 3609 | DCAR_031873 |
| 3610 | DCAR_031877 |
| 3611 | DCAR_031993 |
| 3612 | DCAR_032010 |
| 3613 | DCAR_032012 |
| 3614 | DCAR_032067 |
| 3615 | DCAR_032139 |
| 3616 | DCAR_032140 |
| 3617 | DCAR_032154 |
| 3618 | DCAR_032171 |
| 3619 | DCAR_032186 |
| 3620 | DCAR_032192 |
| 3621 | DCAR_032211 |
| 3622 | DCAR_032234 |
| 3623 | DCAR_032248 |
| 3624 | DCAR_032251 |
| 3625 | DCAR_032252 |
| 3626 | DCAR_032428 |
| 3627 | DcCysP8 |
| 3628 | DcNADP-ICDH1 |
| 3629 | DcPA |
| 3630 | DcPAL4 |
| 3631 | DXR |
| 3632 | ECPP44 |
| 3633 | EFTS |
| 3634 | EIF6 |
| 3635 | F3H |
| 3636 | FATA |
| 3637 | FPS |
| 3638 | GATB |
| 3639 | GGR-1 |
| 3640 | GLN2 |
| 3641 | GMP |
| 3642 | H3-7 |
| 3643 | h4 |
| 3644 | HDR3 |
| 3645 | HDS-1 |
| 3646 | HDS-2 |
| 3647 | HRGP |
| 3648 | idhc |
| 3649 | IDI |
| 3650 | INV*DC4 |
| 3651 | INV1 |
| 3652 | IPPI |
| 3653 | IPPI-2 |
| 3654 | LCYE |
| 3655 | LHC0 |
| 3656 | LSM5 |
| 3657 | LSM8 |
| 3658 | MAA45 |
| 3659 | MaT2 |
| 3660 | matK |
| 3661 | MECPS |
| 3662 | MKK2 |
| 3663 | MPK3b |
| 3664 | MTD |
| 3665 | MVD |
| 3666 | MVK |
| 3667 | nad1 |
| 3668 | nad2 |
| 3669 | nad4 |
| 3670 | nad6 |
| 3671 | nad9 |
| 3672 | NADP-ME |
| 3673 | ndhB |
| 3674 | ndhE |
| 3675 | NMCP1-like |
| 3676 | OSCCAS |
| 3677 | OSCCCS |
| 3678 | PAL1 |
| 3679 | PCM3 |
| 3680 | PCUBI4-1 |
| 3681 | pDAHPS2 |
| 3682 | PDS |
| 3683 | petG |
| 3684 | PFP-ALPHA |
| 3685 | POR1 |
| 3686 | PP2-1 |
| 3687 | PR2 |
| 3688 | PRF5 |
| 3689 | psaJ |
| 3690 | psbC |
| 3691 | psbD |
| 3692 | psbF |
| 3693 | psbL |
| 3694 | psbN |
| 3695 | PURA |
| 3696 | rpl14 |
| 3697 | rpl2 |
| 3698 | rpl23 |
| 3699 | RPL23A |
| 3700 | rpl32 |
| 3701 | RPL36 |
| 3702 | rps11 |
| 3703 | rps12-A |
| 3704 | rps14 |
| 3705 | RPS15A |
| 3706 | rps18 |
| 3707 | rps19 |
| 3708 | rps7-A |
| 3709 | RRF |
| 3710 | SAHH |
| 3711 | SAMS2 |
| 3712 | SAT |
| 3713 | SQS |
| 3714 | STE24 |
| 3715 | TBA |
| 3716 | THI1 |
| 3717 | TUBB1 |
| 3718 | TUBB2 |
| 3719 | UGT709K1 |
| 3720 | UGT709L1 |
| 3721 | UGT71A31 |
| 3722 | UGT71A32 |
| 3723 | UGT73A22 |
| 3724 | UGT73AD1 |
| 3725 | UGT73AH1 |
| 3726 | UGT74AG2 |
| 3727 | UGT84K1 |
| 3728 | UGT87K2 |
| 3729 | UGT94M2 |
| 3730 | ycf2-A |
| 3731 | ycf3 |
| 3732 | ycf4 |
| 3733 | ZDS-1 |
| 3734 | ZEP |

Table S4

**Table S4. List of Centella Asiatica-derived exosomes (CAEs) lipids**

| No. | Rej. | Lipid Ion | Ion Formula |
| --- | --- | --- | --- |
| 1 | NEG100 | Cer(t18:0_18:0)+HCOO | C37 H74 O6 N1 |
| 2 | NEG102 | Cer(t17:0_19:1)+HCOO | C37 H72 O6 N1 |
| 3 | NEG103 | Cer(t17:1_19:0)+HCOO | C37 H72 O6 N1 |
| 4 | NEG109 | Cer(t18:1_18:3)+HCOO | C37 H66 O6 N1 |
| 5 | NEG113 | Cer(t18:0_20:0)+HCOO | C39 H78 O6 N1 |
| 6 | NEG116 | Cer(t18:1_20:0)+HCOO | C39 H76 O6 N1 |
| 7 | NEG118 | Cer(t18:0_21:0)+HCOO | C40 H80 O6 N1 |
| 8 | NEG120 | Cer(t18:1_21:0)+HCOO | C40 H78 O6 N1 |
| 9 | NEG122 | Cer(t18:0_22:0)+HCOO | C41 H82 O6 N1 |
| 10 | NEG127 | Cer(t18:1_22:0)+HCOO | C41 H80 O6 N1 |
| 11 | NEG131 | Cer(t18:0_23:0)+HCOO | C42 H84 O6 N1 |
| 12 | NEG134 | Cer(t18:1_23:0)+HCOO | C42 H82 O6 N1 |
| 13 | NEG139 | Cer(t18:0_24:0)+HCOO | C43 H86 O6 N1 |
| 14 | NEG144 | Cer(t18:1_24:0)+HCOO | C43 H84 O6 N1 |
| 15 | NEG153 | Cer(t18:0_25:0)+HCOO | C44 H88 O6 N1 |
| 16 | NEG155 | Cer(t18:1_25:0)+HCOO | C44 H86 O6 N1 |
| 17 | NEG161 | Cer(t18:1_26:0)+HCOO | C45 H88 O6 N1 |
| 18 | NEG206 | DGDG(14:0_20:5)+HCOO | C50 H83 O17 |
| 19 | NEG216 | DGDG(18:3_18:3)+HCOO | C52 H85 O17 |
| 20 | NEG221 | DGDG(18:2_21:0)+HCOO | C55 H99 O17 |
| 21 | NEG224 | DGDG(18:2_24:0)+HCOO | C58 H105 O17 |
| 22 | NEG312 | LPA(18:2)-H | C21 H38 O7 N0 P1 |
| 23 | NEG316 | LPC(16:0)+HCOO | C25 H51 O9 N1 P1 |
| 24 | NEG318 | LPC(18:0)+HCOO | C27 H55 O9 N1 P1 |
| 25 | NEG319 | LPC(18:1)+HCOO | C27 H53 O9 N1 P1 |
| 26 | NEG320 | LPC(18:2)+HCOO | C27 H51 O9 N1 P1 |
| 27 | NEG325 | LPE(18:2)-H | C23 H43 O7 N1 P1 |
| 28 | NEG327 | LPI(18:2)-H | C27 H48 O12 N0 P1 |
| 29 | NEG346 | MGDG(18:3_16:3)+HCOO | C44 H71 O12 |
| 30 | NEG355 | MGDG(18:3_18:3)+HCOO | C46 H75 O12 |
| 31 | NEG371 | PA(8:1e_10:1)-H | C21 H38 O7 N0 P1 |
| 32 | NEG379 | PA(15:0_18:2)-H | C36 H66 O8 N0 P1 |
| 33 | NEG38 | Cer(d18:1_18:0)+HCOO | C37 H72 O5 N1 |
| 34 | NEG384 | PA(16:0_18:2)-H | C37 H68 O8 N0 P1 |
| 35 | NEG393 | PA(16:0_18:3)-H | C37 H66 O8 N0 P1 |
| 36 | NEG395 | PA(18:4_16:0)-H | C37 H64 O8 N0 P1 |
| 37 | NEG400 | PA(17:0_18:2)-H | C38 H70 O8 N0 P1 |
| 38 | NEG406 | PA(18:0_18:2)-H | C39 H72 O8 N0 P1 |
| 39 | NEG413 | PA(18:2_18:2)-H | C39 H68 O8 N0 P1 |
| 40 | NEG424 | PA(18:3_18:2)-H | C39 H66 O8 N0 P1 |
| 41 | NEG427 | PA(18:3_18:3)-H | C39 H64 O8 N0 P1 |
| 42 | NEG433 | PA(20:0_18:2)-H | C41 H76 O8 N0 P1 |
| 43 | NEG438 | PA(20:1_18:2)-H | C41 H74 O8 N0 P1 |
| 44 | NEG439 | PA(20:2_18:2)-H | C41 H72 O8 N0 P1 |
| 45 | NEG441 | PA(18:2_21:0)-H | C42 H78 O8 N0 P1 |
| 46 | NEG445 | PA(18:3_21:1)-H | C42 H74 O8 N0 P1 |
| 47 | NEG447 | PA(22:0_18:2)-H | C43 H80 O8 N0 P1 |
| 48 | NEG453 | PA(18:2_23:0)-H | C44 H82 O8 N0 P1 |
| 49 | NEG460 | PC(16:0_14:0)+HCOO | C39 H77 O10 N1 P1 |
| 50 | NEG461 | PC(16:0_16:0)+HCOO | C41 H81 O10 N1 P1 |
| 51 | NEG462 | PC(16:0_16:1)+HCOO | C41 H79 O10 N1 P1 |
| 52 | NEG463 | PC(18:0_16:0)+HCOO | C43 H85 O10 N1 P1 |
| 53 | NEG464 | PC(16:0_18:1)+HCOO | C43 H83 O10 N1 P1 |
| 54 | NEG465 | PC(16:0_18:2)+HCOO | C43 H81 O10 N1 P1 |
| 55 | NEG466 | PC(16:1_18:2)+HCOO | C43 H79 O10 N1 P1 |
| 56 | NEG467 | PC(18:0_18:1)+HCOO | C45 H87 O10 N1 P1 |
| 57 | NEG468 | PC(18:0_18:2)+HCOO | C45 H85 O10 N1 P1 |
| 58 | NEG469 | PC(18:1_18:2)+HCOO | C45 H83 O10 N1 P1 |
| 59 | NEG470 | PC(16:0_20:4)+HCOO | C45 H81 O10 N1 P1 |
| 60 | NEG471 | PC(16:0_20:5)+HCOO | C45 H79 O10 N1 P1 |
| 61 | NEG472 | PC(18:0_20:4)+HCOO | C47 H85 O10 N1 P1 |
| 62 | NEG473 | PC(18:1_20:4)+HCOO | C47 H83 O10 N1 P1 |
| 63 | NEG474 | PC(16:0_22:6)+HCOO | C47 H81 O10 N1 P1 |
| 64 | NEG475 | PC(18:0_22:6)+HCOO | C49 H85 O10 N1 P1 |
| 65 | NEG478 | PE(16:0_18:1)-H | C39 H75 O8 N1 P1 |
| 66 | NEG48 | Cer(d15:1_26:0)+HCOO | C42 H82 O5 N1 |
| 67 | NEG480 | PE(16:0_18:2)-H | C39 H73 O8 N1 P1 |
| 68 | NEG481 | PE(16:0_18:3)-H | C39 H71 O8 N1 P1 |
| 69 | NEG483 | PE(18:0_18:1)-H | C41 H79 O8 N1 P1 |
| 70 | NEG484 | PE(18:1_18:1)-H | C41 H77 O8 N1 P1 |
| 71 | NEG489 | PE(18:2_18:2)-H | C41 H73 O8 N1 P1 |
| 72 | NEG494 | PE(20:0_18:2)-H | C43 H81 O8 N1 P1 |
| 73 | NEG495 | PE(20:1_18:2)-H | C43 H79 O8 N1 P1 |
| 74 | NEG496 | PE(18:0_20:4)-H | C43 H77 O8 N1 P1 |
| 75 | NEG500 | PE(18:2_21:0)-H | C44 H83 O8 N1 P1 |
| 76 | NEG502 | PE(22:0_18:2)-H | C45 H85 O8 N1 P1 |
| 77 | NEG505 | PE(18:0_22:6)-H | C45 H77 O8 N1 P1 |
| 78 | NEG508 | PE(18:2_23:0)-H | C46 H87 O8 N1 P1 |
| 79 | NEG509 | PE(24:0_18:2)-H | C47 H89 O8 N1 P1 |
| 80 | NEG53 | Cer(d18:1_24:0)+HCOO | C43 H84 O5 N1 |
| 81 | NEG562 | PI(16:0_16:0)-H | C41 H78 O13 N0 P1 |
| 82 | NEG563 | PI(16:0_16:1)-H | C41 H76 O13 N0 P1 |
| 83 | NEG565 | PI(16:0_18:1)-H | C43 H80 O13 N0 P1 |
| 84 | NEG566 | PI(16:0_18:2)-H | C43 H78 O13 N0 P1 |
| 85 | NEG567 | PI(16:0_18:3)-H | C43 H76 O13 N0 P1 |
| 86 | NEG568 | PI(18:4_16:0)-H | C43 H74 O13 N0 P1 |
| 87 | NEG569 | PI(17:0_18:2)-H | C44 H80 O13 N0 P1 |
| 88 | NEG570 | PI(18:0_18:1)-H | C45 H84 O13 N0 P1 |
| 89 | NEG571 | PI(18:0_18:2)-H | C45 H82 O13 N0 P1 |
| 90 | NEG572 | PI(18:1_18:2)-H | C45 H80 O13 N0 P1 |
| 91 | NEG573 | PI(18:2_18:2)-H | C45 H78 O13 N0 P1 |
| 92 | NEG574 | PI(18:3_18:2)-H | C45 H76 O13 N0 P1 |
| 93 | NEG575 | PI(18:3_18:3)-H | C45 H74 O13 N0 P1 |
| 94 | NEG576 | PI(18:0_20:4)-H | C47 H82 O13 N0 P1 |
| 95 | NEG592 | PS(20:0_18:2)-H | C44 H81 O10 N1 P1 |
| 96 | NEG597 | PS(18:2_21:0)-H | C45 H83 O10 N1 P1 |
| 97 | NEG598 | PS(22:0_18:2)-H | C46 H85 O10 N1 P1 |
| 98 | NEG617 | SQDG(16:0_16:0)-H | C41 H77 O12 S1 |
| 99 | NEG639 | SQDG(18:2_18:2)-H | C45 H77 O12 S1 |
| 100 | NEG641 | SQDG(18:3_18:3)-H | C45 H73 O12 S1 |
| 101 | NEG7 | CL(18:2_18:2_18:2_18:2)-H | C81 H141 O17 P2 |
| 102 | NEG83 | Cer(t18:0_16:0)+HCOO | C35 H70 O6 N1 |
| 103 | NEG87 | Cer(t18:1_16:0)+HCOO | C35 H68 O6 N1 |
| 104 | NEG89 | Cer(t16:1_18:0)+HCOO | C35 H68 O6 N1 |
| 105 | NEG93 | Cer(t16:1_18:1)+HCOO | C35 H66 O6 N1 |
| 106 | NEG98 | Cer(t17:0_18:1)+HCOO | C36 H70 O6 N1 |
| 107 | POS1000 | TG(16:0_18:2_21:0)+NH4 | C58 H112 O6 N1 |
| 108 | POS1002 | TG(19:0_18:2_18:2)+NH4 | C58 H108 O6 N1 |
| 109 | POS1006 | TG(16:0_18:2_22:0)+Na | C59 H110 O6 Na1 |
| 110 | POS1007 | TG(16:0_18:3_22:0)+NH4 | C59 H112 O6 N1 |
| 111 | POS1008 | TG(20:0_18:2_18:2)+NH4 | C59 H110 O6 N1 |
| 112 | POS1010 | TG(20:0_18:2_18:3)+NH4 | C59 H108 O6 N1 |
| 113 | POS1013 | TG(16:0_18:2_23:0)+NH4 | C60 H116 O6 N1 |
| 114 | POS1014 | TG(18:2_18:2_21:0)+NH4 | C60 H112 O6 N1 |
| 115 | POS1015 | TG(18:3_18:2_21:0)+NH4 | C60 H110 O6 N1 |
| 116 | POS1019 | TG(22:0_18:2_18:2)+NH4 | C61 H114 O6 N1 |
| 117 | POS1020 | TG(22:1_18:2_18:2)+NH4 | C61 H112 O6 N1 |
| 118 | POS1022 | TG(18:2_18:2_23:0)+NH4 | C62 H116 O6 N1 |
| 119 | POS1026 | TG(24:0_18:2_18:2)+NH4 | C63 H118 O6 N1 |
| 120 | POS1027 | TG(22:0_18:2_20:5)+H | C63 H109 O6 |
| 121 | POS193 | Co(Q10)+NH4 | C59 H94 O4 N1 |
| 122 | POS194 | Co(Q9)+H | C54 H83 O4 |
| 123 | POS250 | DG(16:0_18:1)+NH4 | C37 H74 O5 N1 |
| 124 | POS252 | DG(16:0_18:2)+NH4 | C37 H72 O5 N1 |
| 125 | POS257 | DG(16:0_18:3)+H | C37 H67 O5 |
| 126 | POS258 | DG(16:0_18:3)+NH4 | C37 H70 O5 N1 |
| 127 | POS269 | DG(18:4_16:0)+NH4 | C37 H68 O5 N1 |
| 128 | POS280 | DG(17:0_18:2)+NH4 | C38 H74 O5 N1 |
| 129 | POS282 | DG(18:0_18:2)+NH4 | C39 H76 O5 N1 |
| 130 | POS283 | DG(18:1_18:2)+NH4 | C39 H74 O5 N1 |
| 131 | POS287 | DG(18:2_18:2)+H | C39 H69 O5 |
| 132 | POS288 | DG(18:2_18:2)+NH4 | C39 H72 O5 N1 |
| 133 | POS294 | DG(18:3_18:2)+H | C39 H67 O5 |
| 134 | POS295 | DG(18:3_18:2)+NH4 | C39 H70 O5 N1 |
| 135 | POS299 | DG(18:3_18:3)+H | C39 H65 O5 |
| 136 | POS301 | DG(18:3_18:3)+NH4 | C39 H68 O5 N1 |
| 137 | POS304 | DG(18:4_18:3)+H | C39 H63 O5 |
| 138 | POS307 | DG(20:0_18:2)+NH4 | C41 H80 O5 N1 |
| 139 | POS308 | DG(20:1_18:2)+NH4 | C41 H78 O5 N1 |
| 140 | POS310 | DG(20:2_18:2)+NH4 | C41 H76 O5 N1 |
| 141 | POS314 | DG(18:2_21:0)+NH4 | C42 H82 O5 N1 |
| 142 | POS315 | DG(22:0_18:2)+NH4 | C43 H84 O5 N1 |
| 143 | POS321 | DG(18:2_23:0)+NH4 | C44 H86 O5 N1 |
| 144 | POS322 | DG(24:0_18:2)+NH4 | C45 H88 O5 N1 |
| 145 | POS333 | DGDG(16:0_16:0)+Na | C47 H88 O15 Na1 |
| 146 | POS335 | DGDG(16:0_16:1)+Na | C47 H86 O15 Na1 |
| 147 | POS337 | DGDG(16:1_16:1)+Na | C47 H84 O15 Na1 |
| 148 | POS338 | DGDG(16:0_18:0)+Na | C49 H92 O15 Na1 |
| 149 | POS340 | DGDG(16:0_18:1)+Na | C49 H90 O15 Na1 |
| 150 | POS343 | DGDG(16:0_18:2)+Na | C49 H88 O15 Na1 |
| 151 | POS344 | DGDG(16:0_18:3)+Na | C49 H86 O15 Na1 |
| 152 | POS346 | DGDG(18:2_16:2)+Na | C49 H84 O15 Na1 |
| 153 | POS348 | DGDG(18:3_16:2)+Na | C49 H82 O15 Na1 |
| 154 | POS350 | DGDG(18:3_16:3)+Na | C49 H80 O15 Na1 |
| 155 | POS353 | DGDG(17:0_18:2)+Na | C50 H90 O15 Na1 |
| 156 | POS354 | DGDG(17:0_18:3)+Na | C50 H88 O15 Na1 |
| 157 | POS355 | DGDG(18:0_18:1)+NH4 | C51 H98 O15 N1 |
| 158 | POS356 | DGDG(18:0_18:1)+Na | C51 H94 O15 Na1 |
| 159 | POS360 | DGDG(18:0_18:2)+Na | C51 H92 O15 Na1 |
| 160 | POS361 | DGDG(18:0_18:3)+Na | C51 H90 O15 Na1 |
| 161 | POS363 | DGDG(18:2_18:2)+Na | C51 H88 O15 Na1 |
| 162 | POS365 | DGDG(18:2_18:3)+Na | C51 H86 O15 Na1 |
| 163 | POS367 | DGDG(16:0_20:5)+Na | C51 H86 O15 Na1 |
| 164 | POS369 | DGDG(18:3_18:3)+Na | C51 H84 O15 Na1 |
| 165 | POS374 | DGDG(20:0_18:2)+Na | C53 H96 O15 Na1 |
| 166 | POS378 | DGDG(18:3_20:3)+NH4 | C53 H92 O15 N1 |
| 167 | POS379 | DGDG(18:2_22:0)+Na | C55 H100 O15 Na1 |
| 168 | POS382 | DGDG(18:2_23:0)+Na | C56 H102 O15 Na1 |
| 169 | POS383 | DGDG(18:2_25:0)+NH4 | C58 H110 O15 N1 |
| 170 | POS387 | Hex1Cer(d18:1_16:0+O)+H | C40 H78 O9 N1 |
| 171 | POS390 | Hex1Cer(d18:2_16:0+O)+H | C40 H76 O9 N1 |
| 172 | POS391 | Hex1Cer(d19:2_16:0+O)+H | C41 H78 O9 N1 |
| 173 | POS393 | Hex1Cer(d19:2_18:1+O)+H | C43 H80 O9 N1 |
| 174 | POS396 | Hex1Cer(d18:2_20:0+O)+H | C44 H84 O9 N1 |
| 175 | POS400 | Hex1Cer(d18:2_22:0+O)+H | C46 H88 O9 N1 |
| 176 | POS403 | Hex1Cer(d18:2_23:0+O)+H | C47 H90 O9 N1 |
| 177 | POS406 | Hex1Cer(d18:2_24:0+O)+H | C48 H92 O9 N1 |
| 178 | POS408 | Hex1Cer(d18:2_25:0+O)+H | C49 H94 O9 N1 |
| 179 | POS418 | Hex1Cer(t17:1_15:0+O)+H | C38 H74 O10 N1 |
| 180 | POS420 | Hex1Cer(t18:1_16:0+O)+H | C40 H78 O10 N1 |
| 181 | POS425 | Hex1Cer(t18:0_22:0+O)+H | C46 H92 O10 N1 |
| 182 | POS428 | Hex1Cer(t18:1_22:0+O)+H | C46 H90 O10 N1 |
| 183 | POS441 | Hex1SPH(t18:1)+H | C24 H48 O8 N1 |
| 184 | POS486 | MGDG(16:0_20:5)+NH4 | C45 H80 O10 N1 |
| 185 | POS487 | MGDG(18:3_18:3)+NH4 | C45 H78 O10 N1 |
| 186 | POS493 | MGDG(18:3_20:2)+NH4 | C47 H84 O10 N1 |
| 187 | POS508 | PA(16:0_18:2)+NH4 | C37 H73 O8 N1 P1 |
| 188 | POS531 | PA(16:0_18:3)+NH4 | C37 H71 O8 N1 P1 |
| 189 | POS535 | PA(18:1_18:1)+NH4 | C39 H77 O8 N1 P1 |
| 190 | POS536 | PA(18:0_18:2)+NH4 | C39 H77 O8 N1 P1 |
| 191 | POS545 | PA(18:2_18:2)+NH4 | C39 H73 O8 N1 P1 |
| 192 | POS561 | PA(18:3_18:2)+NH4 | C39 H71 O8 N1 P1 |
| 193 | POS575 | PA(22:0_18:2)+NH4 | C43 H85 O8 N1 P1 |
| 194 | POS588 | PC(18:0e_6:0)+H | C32 H67 O7 N1 P1 |
| 195 | POS595 | PC(16:1_16:1)+H | C40 H77 O8 N1 P1 |
| 196 | POS600 | PC(22:0_12:1)+H | C42 H83 O8 N1 P1 |
| 197 | POS616 | PC(18:2_18:2)+Na | C44 H80 O8 N1 P1 Na1 |
| 198 | POS644 | PE(16:0_18:1)+H | C39 H77 O8 N1 P1 |
| 199 | POS646 | PE(16:0_18:2)+H | C39 H75 O8 N1 P1 |
| 200 | POS668 | PE(18:1_18:2)+H | C41 H77 O8 N1 P1 |
| 201 | POS672 | PE(16:0_20:4)+H | C41 H75 O8 N1 P1 |
| 202 | POS673 | PE(16:0p_20:4)+H | C41 H75 O7 N1 P1 |
| 203 | POS687 | PE(16:0p_22:6)+H | C43 H75 O7 N1 P1 |
| 204 | POS694 | PE(18:0p_22:6)+H | C45 H79 O7 N1 P1 |
| 205 | POS709 | PG(16:0_18:2)+NH4 | C40 H79 O10 N1 P1 |
| 206 | POS742 | PI(18:0_18:2)+NH4 | C45 H87 O13 N1 P1 |
| 207 | POS773 | PS(22:0_18:2)+H | C46 H87 O10 N1 P1 |
| 208 | POS823 | SQDG(16:0_16:1)+NH4 | C41 H80 O12 S1 N1 |
| 209 | POS833 | SQDG(18:0_18:2)+NH4 | C45 H86 O12 S1 N1 |
| 210 | POS834 | SQDG(18:1_18:2)+NH4 | C45 H84 O12 S1 N1 |
| 211 | POS868 | TG(8:0_8:0_11:4)+NH4 | C30 H52 O6 N1 |
| 212 | POS871 | TG(9:0_11:3_12:0)+NH4 | C35 H64 O6 N1 |
| 213 | POS872 | TG(9:0_11:3_12:1)+NH4 | C35 H62 O6 N1 |
| 214 | POS873 | TG(4:0_11:3_18:3)+H | C36 H57 O6 |
| 215 | POS874 | TG(4:0_11:0_19:1)+NH4 | C37 H72 O6 N1 |
| 216 | POS875 | TG(16:0_8:0_10:1)+NH4 | C37 H72 O6 N1 |
| 217 | POS876 | TG(16:0_6:0_12:2)+Na | C37 H66 O6 Na1 |
| 218 | POS877 | TG(16:0_6:0_12:2)+H | C37 H67 O6 |
| 219 | POS878 | TG(12:1e_11:0_11:3)+H | C37 H65 O5 |
| 220 | POS887 | TG(4:0_14:1_18:2)+NH4 | C39 H72 O6 N1 |
| 221 | POS890 | TG(6:0_12:2_18:2)+H | C39 H67 O6 |
| 222 | POS891 | TG(16:0_6:0_14:4)+H | C39 H67 O6 |
| 223 | POS903 | TG(6:0_14:4_18:2)+H | C41 H67 O6 |
| 224 | POS904 | TG(9:0_11:4_18:3)+H | C41 H65 O6 |
| 225 | POS915 | TG(16:0_11:2_14:2)+H | C44 H77 O6 |
| 226 | POS919 | TG(16:0_14:0_14:0)+NH4 | C47 H94 O6 N1 |
| 227 | POS923 | TG(22:5_11:2_12:3)+NH4 | C48 H76 O6 N1 |
| 228 | POS924 | TG(16:0_14:0_16:0)+NH4 | C49 H98 O6 N1 |
| 229 | POS925 | TG(16:0_14:0_16:1)+NH4 | C49 H96 O6 N1 |
| 230 | POS926 | TG(15:0_16:0_16:0)+NH4 | C50 H100 O6 N1 |
| 231 | POS927 | TG(15:0_16:0_16:1)+NH4 | C50 H98 O6 N1 |
| 232 | POS933 | TG(16:0_16:0_16:0)+NH4 | C51 H102 O6 N1 |
| 233 | POS935 | TG(16:0_16:0_16:1)+NH4 | C51 H100 O6 N1 |
| 234 | POS936 | TG(16:0_16:1_16:1)+NH4 | C51 H98 O6 N1 |
| 235 | POS937 | TG(16:1_16:1_16:1)+NH4 | C51 H96 O6 N1 |
| 236 | POS938 | TG(16:0_16:0_17:0)+NH4 | C52 H104 O6 N1 |
| 237 | POS939 | TG(15:0_16:0_18:1)+NH4 | C52 H102 O6 N1 |
| 238 | POS941 | TG(15:0_16:0_18:2)+NH4 | C52 H100 O6 N1 |
| 239 | POS942 | TG(18:0_16:0_16:0)+NH4 | C53 H106 O6 N1 |
| 240 | POS944 | TG(16:0_16:0_18:1)+NH4 | C53 H104 O6 N1 |
| 241 | POS946 | TG(16:0_16:0_18:2)+NH4 | C53 H102 O6 N1 |
| 242 | POS948 | TG(16:0_16:0_18:3)+NH4 | C53 H100 O6 N1 |
| 243 | POS949 | TG(14:0_18:2_18:2)+NH4 | C53 H98 O6 N1 |
| 244 | POS950 | TG(18:2_14:1_18:2)+NH4 | C53 H96 O6 N1 |
| 245 | POS951 | TG(16:0_17:0_18:1)+NH4 | C54 H106 O6 N1 |
| 246 | POS952 | TG(16:0_17:1_18:1)+NH4 | C54 H104 O6 N1 |
| 247 | POS953 | TG(15:0_18:1_18:2)+NH4 | C54 H102 O6 N1 |
| 248 | POS954 | TG(15:0_18:2_18:2)+NH4 | C54 H100 O6 N1 |
| 249 | POS955 | TG(15:0_18:2_18:3)+NH4 | C54 H98 O6 N1 |
| 250 | POS957 | TG(18:3_10:3_23:0)+NH4 | C54 H96 O6 N1 |
| 251 | POS958 | TG(18:0_16:0_18:0)+NH4 | C55 H110 O6 N1 |
| 252 | POS960 | TG(18:0_16:0_18:1)+NH4 | C55 H108 O6 N1 |
| 253 | POS962 | TG(16:0_18:1_18:1)+NH4 | C55 H106 O6 N1 |
| 254 | POS966 | TG(16:0_18:1_18:2)+NH4 | C55 H104 O6 N1 |
| 255 | POS968 | TG(16:0_18:2_18:2)+NH4 | C55 H102 O6 N1 |
| 256 | POS970 | TG(16:0_18:2_18:3)+H | C55 H97 O6 |
| 257 | POS971 | TG(16:0_18:2_18:3)+NH4 | C55 H100 O6 N1 |
| 258 | POS973 | TG(16:0_18:3_18:3)+NH4 | C55 H98 O6 N1 |
| 259 | POS975 | TG(18:2_11:4_23:1)+NH4 | C55 H96 O6 N1 |
| 260 | POS976 | TG(18:3_11:4_23:1)+NH4 | C55 H94 O6 N1 |
| 261 | POS977 | TG(18:0_17:0_18:1)+NH4 | C56 H110 O6 N1 |
| 262 | POS978 | TG(17:0_18:1_18:1)+NH4 | C56 H108 O6 N1 |
| 263 | POS979 | TG(17:0_18:1_18:2)+NH4 | C56 H106 O6 N1 |
| 264 | POS980 | TG(17:0_18:2_18:2)+NH4 | C56 H104 O6 N1 |
| 265 | POS981 | TG(17:0_18:2_18:3)+NH4 | C56 H102 O6 N1 |
| 266 | POS982 | TG(15:0_18:2_20:5)+H | C56 H95 O6 |
| 267 | POS985 | TG(18:0_18:0_18:1)+NH4 | C57 H112 O6 N1 |
| 268 | POS986 | TG(18:0_18:1_18:1)+NH4 | C57 H110 O6 N1 |
| 269 | POS987 | TG(18:0_18:1_18:2)+NH4 | C57 H108 O6 N1 |
| 270 | POS989 | TG(18:0_18:2_18:2)+NH4 | C57 H106 O6 N1 |
| 271 | POS991 | TG(18:0_18:2_18:3)+NH4 | C57 H104 O6 N1 |
| 272 | POS993 | TG(18:2_18:2_18:2)+NH4 | C57 H102 O6 N1 |
| 273 | POS995 | TG(18:3_18:2_18:2)+NH4 | C57 H100 O6 N1 |
| 274 | POS997 | TG(18:3_18:2_18:3)+NH4 | C57 H98 O6 N1 |
| 275 | POS999 | TG(18:3_18:3_18:3)+NH4 | C57 H96 O6 N1 |
| 276 | NEG101 | Cer(t17:0_19:0+O)+HCOO | C37 H74 O7 N1 |
| 277 | NEG105 | Cer(t18:1_18:1)+HCOO | C37 H70 O6 N1 |
| 278 | NEG106 | Cer(t18:1_18:1+O)+HCOO | C37 H70 O7 N1 |
| 279 | NEG111 | Cer(t17:1_20:0+O)+HCOO | C38 H74 O7 N1 |
| 280 | NEG112 | Cer(t17:0_20:1+O)-H | C37 H72 O5 N1 |
| 281 | NEG114 | Cer(t18:0_20:0+O)+HCOO | C39 H78 O7 N1 |
| 282 | NEG117 | Cer(t17:1_21:0+O)+HCOO | C39 H76 O7 N1 |
| 283 | NEG119 | Cer(t17:0_22:0+O)+HCOO | C40 H80 O7 N1 |
| 284 | NEG121 | Cer(t17:1_22:0+O)+HCOO | C40 H78 O7 N1 |
| 285 | NEG125 | Cer(t17:0_23:0+O)+HCOO | C41 H82 O7 N1 |
| 286 | NEG126 | Cer(t20:0_20:1)+HCOO | C41 H80 O6 N1 |
| 287 | NEG129 | Cer(t17:1_23:0+O)+HCOO | C41 H80 O7 N1 |
| 288 | NEG133 | Cer(t17:0_24:0+O)+HCOO | C42 H84 O7 N1 |
| 289 | NEG136 | Cer(t17:1_24:0+O)+HCOO | C42 H82 O7 N1 |
| 290 | NEG141 | Cer(t17:0_25:0+O)+HCOO | C43 H86 O7 N1 |
| 291 | NEG143 | Cer(t16:0_26:1)+HCOO | C43 H84 O6 N1 |
| 292 | NEG147 | Cer(t17:1_25:0+O)+HCOO | C43 H84 O7 N1 |
| 293 | NEG154 | Cer(t17:0_26:0+O)+HCOO | C44 H88 O7 N1 |
| 294 | NEG157 | Cer(t17:1_26:0+O)+HCOO | C44 H86 O7 N1 |
| 295 | NEG229 | DGMG(16:0)+HCOO | C32 H59 O16 |
| 296 | NEG230 | DGMG(18:0)+HCOO | C34 H63 O16 |
| 297 | NEG231 | DGMG(18:1)+HCOO | C34 H61 O16 |
| 298 | NEG232 | DGMG(18:2)+HCOO | C34 H59 O16 |
| 299 | NEG233 | DGMG(18:3)+HCOO | C34 H57 O16 |
| 300 | NEG272 | Hex1Cer(t16:0_18:2)-H | C40 H74 O9 N1 |
| 301 | NEG28 | Cer(d15:1_17:1)+HCOO | C33 H62 O5 N1 |
| 302 | NEG29 | Cer(d15:1_18:1)+HCOO | C34 H64 O5 N1 |
| 303 | NEG317 | LPC(16:1)-CH3 | C23 H45 O7 N1 P1 |
| 304 | NEG321 | LPE(16:0)-H | C21 H43 O7 N1 P1 |
| 305 | NEG322 | LPE(18:0)-H | C23 H47 O7 N1 P1 |
| 306 | NEG323 | LPE(18:1)-H | C23 H45 O7 N1 P1 |
| 307 | NEG326 | LPG(16:1)-H | C22 H42 O9 N0 P1 |
| 308 | NEG339 | MGDG(16:0_18:2)+HCOO | C44 H79 O12 |
| 309 | NEG34 | Cer(d18:1_16:0)+HCOO | C35 H68 O5 N1 |
| 310 | NEG341 | MGDG(16:0_18:3)+HCOO | C44 H77 O12 |
| 311 | NEG343 | MGDG(18:2_16:3)+HCOO | C44 H73 O12 |
| 312 | NEG36 | Cer(d18:0_16:1+O)+HCOO | C35 H68 O6 N1 |
| 313 | NEG365 | MGMG(16:0)+HCOO | C26 H49 O11 |
| 314 | NEG366 | MGMG(16:3)+HCOO | C26 H43 O11 |
| 315 | NEG367 | MGMG(18:2)+HCOO | C28 H49 O11 |
| 316 | NEG368 | MGMG(18:3)+HCOO | C28 H47 O11 |
| 317 | NEG369 | MGMG(27:6)+HCOO | C37 H59 O11 |
| 318 | NEG37 | Cer(d18:2_16:0)+HCOO | C35 H66 O5 N1 |
| 319 | NEG373 | PA(16:0_16:0)-H | C35 H68 O8 N0 P1 |
| 320 | NEG380 | PA(19:0_14:3)-H | C36 H64 O8 N0 P1 |
| 321 | NEG39 | Cer(d18:0_18:1+2O)+HCOO | C37 H72 O7 N1 |
| 322 | NEG397 | PA(17:0_18:1)-H | C38 H72 O8 N0 P1 |
| 323 | NEG40 | Cer(d23:2_13:0)+HCOO | C37 H70 O5 N1 |
| 324 | NEG405 | PA(19:1_17:0)-H | C39 H74 O8 N0 P1 |
| 325 | NEG41 | Cer(d21:2_15:0)+HCOO | C37 H70 O5 N1 |
| 326 | NEG428 | PA(19:0_18:1)-H | C40 H76 O8 N0 P1 |
| 327 | NEG430 | PA(19:1_18:2)-H | C40 H72 O8 N0 P1 |
| 328 | NEG476 | PE(16:0_16:0)-H | C37 H73 O8 N1 P1 |
| 329 | NEG477 | PE(17:1_16:0)-H | C38 H73 O8 N1 P1 |
| 330 | NEG482 | PE(17:0_18:2)-H | C40 H75 O8 N1 P1 |
| 331 | NEG486 | PE(18:1_18:2)-H | C41 H75 O8 N1 P1 |
| 332 | NEG490 | PE(18:3_18:2)-H | C41 H71 O8 N1 P1 |
| 333 | NEG492 | PE(16:1e_20:4)-H | C41 H73 O7 N1 P1 |
| 334 | NEG493 | PE(18:3_18:3)-H | C41 H69 O8 N1 P1 |
| 335 | NEG497 | PE(18:1e_20:4)-H | C43 H77 O7 N1 P1 |
| 336 | NEG501 | PE(18:1_22:0)-H | C45 H87 O8 N1 P1 |
| 337 | NEG503 | PE(18:0_22:4)-H | C45 H81 O8 N1 P1 |
| 338 | NEG506 | PE(18:1e_22:6)-H | C45 H77 O7 N1 P1 |
| 339 | NEG510 | PG(20:0_11:2)-H | C37 H68 O10 N0 P1 |
| 340 | NEG511 | PG(20:1_11:2)-H | C37 H66 O10 N0 P1 |
| 341 | NEG512 | PG(16:0_16:0)-H | C38 H74 O10 N0 P1 |
| 342 | NEG513 | PG(16:0_16:1)-H | C38 H72 O10 N0 P1 |
| 343 | NEG516 | PG(21:0_11:2)-H | C38 H70 O10 N0 P1 |
| 344 | NEG519 | PG(18:0_16:0)-H | C40 H78 O10 N0 P1 |
| 345 | NEG520 | PG(16:0_18:1)-H | C40 H76 O10 N0 P1 |
| 346 | NEG521 | PG(18:0_16:1)-H | C40 H76 O10 N0 P1 |
| 347 | NEG522 | PG(16:0_18:2)-H | C40 H74 O10 N0 P1 |
| 348 | NEG523 | PG(16:1_18:1)-H | C40 H74 O10 N0 P1 |
| 349 | NEG524 | PG(16:0_18:3)-H | C40 H72 O10 N0 P1 |
| 350 | NEG525 | PG(16:1_18:2)-H | C40 H72 O10 N0 P1 |
| 351 | NEG527 | PG(16:1_18:3)-H | C40 H70 O10 N0 P1 |
| 352 | NEG532 | PG(12:0_22:5)-H | C40 H68 O10 N0 P1 |
| 353 | NEG540 | PG(18:0_18:1)-H | C42 H80 O10 N0 P1 |
| 354 | NEG541 | PG(18:0_18:2)-H | C42 H78 O10 N0 P1 |
| 355 | NEG542 | PG(18:2_18:2)-H | C42 H74 O10 N0 P1 |
| 356 | NEG543 | PG(16:0_20:5)-H | C42 H72 O10 N0 P1 |
| 357 | NEG546 | PG(16:1_20:5)-H | C42 H70 O10 N0 P1 |
| 358 | NEG577 | PI(22:0_18:2)-H | C49 H90 O13 N0 P1 |
| 359 | NEG591 | PS(18:2_18:2)-H | C42 H73 O10 N1 P1 |
| 360 | NEG6 | CL(18:2_16:0_18:2_18:2)-H | C79 H141 O17 P2 |
| 361 | NEG602 | PS(18:2_23:0)-H | C47 H87 O10 N1 P1 |
| 362 | NEG614 | SQDG(14:0e_17:0)+HCOO | C41 H79 O13 S1 |
| 363 | NEG643 | SQDG(18:3_22:4)+HCOO | C50 H81 O14 S1 |
| 364 | NEG661 | SQMG(16:0)-H | C25 H47 O11 S1 |
| 365 | NEG665 | SQMG(24:1)+HCOO | C34 H63 O13 S1 |
| 366 | NEG666 | SQMG(28:0)+HCOO | C38 H73 O13 S1 |
| 367 | NEG667 | SQMG(29:1)+HCOO | C39 H73 O13 S1 |
| 368 | NEG668 | SQMG(32:0)+HCOO | C42 H81 O13 S1 |
| 369 | NEG669 | SQMG(33:0)+HCOO | C43 H83 O13 S1 |
| 370 | NEG671 | SQMG(34:0)+HCOO | C44 H85 O13 S1 |
| 371 | NEG8 | CL(18:3_18:2_18:2_18:2)-H | C81 H139 O17 P2 |
| 372 | NEG86 | Cer(t17:0_17:0+O)+HCOO | C35 H70 O7 N1 |
| 373 | NEG9 | CL(20:5_18:2_18:2_18:2)-H | C83 H139 O17 P2 |
| 374 | NEG95 | Cer(t16:1_18:2)+HCOO | C35 H64 O6 N1 |
| 375 | NEG96 | Cer(t16:1_18:3)+HCOO | C35 H62 O6 N1 |
| 376 | POS100 | Cer(d18:1_25:0+O)+H | C43 H86 O4 N1 |
| 377 | POS1001 | TG(22:0_11:3_22:1)+NH4 | C58 H108 O6 N1 |
| 378 | POS1005 | TG(22:0_12:2_22:0)+NH4 | C59 H114 O6 N1 |
| 379 | POS101 | Cer(d18:2_25:0+O)+H | C43 H84 O4 N1 |
| 380 | POS1011 | TG(20:0_18:3_18:3)+NH4 | C59 H106 O6 N1 |
| 381 | POS1012 | TG(22:2_12:4_22:2)+NH4 | C59 H102 O6 N1 |
| 382 | POS1017 | TG(22:0_14:2_22:0)+NH4 | C61 H118 O6 N1 |
| 383 | POS1028 | WE(2:0_16:3)+NH4 | H34 C18 O2 N1 |
| 384 | POS1029 | WE(2:0_16:4)+NH4 | H32 C18 O2 N1 |
| 385 | POS103 | Cer(d18:0_26:0)+H | C44 H90 O3 N1 |
| 386 | POS1030 | WE(3:0_16:4)+H | H31 C19 O2 |
| 387 | POS1031 | WE(2:0_18:3)+NH4 | H38 C20 O2 N1 |
| 388 | POS1033 | WE(2:0_20:2)+NH4 | H44 C22 O2 N1 |
| 389 | POS1034 | WE(6:0_16:3)+NH4 | H42 C22 O2 N1 |
| 390 | POS1037 | WE(24:0_17:3)+H | H77 C41 O2 |
| 391 | POS1038 | WE(24:0_17:4)+H | H75 C41 O2 |
| 392 | POS106 | Cer(d18:2_26:0)+H | C44 H86 O3 N1 |
| 393 | POS107 | Cer(d20:0_26:0)+H | C46 H94 O3 N1 |
| 394 | POS108 | Cer(d20:1_26:0)+H | C46 H92 O3 N1 |
| 395 | POS109 | Cer(d24:2_22:5)+H | C46 H80 O3 N1 |
| 396 | POS112 | Cer(m19:0_14:0+O)+H | C33 H68 O3 N1 |
| 397 | POS113 | Cer(m19:1_14:0+O)+H | C33 H66 O3 N1 |
| 398 | POS114 | Cer(m19:0_15:0+O)+H | C34 H70 O3 N1 |
| 399 | POS115 | Cer(m19:1_15:0+O)+H | C34 H68 O3 N1 |
| 400 | POS116 | Cer(m19:1_16:0+2O)+H | C35 H70 O4 N1 |
| 401 | POS117 | Cer(m19:1_16:0+O)+H | C35 H70 O3 N1 |
| 402 | POS118 | Cer(m19:1_16:1+O)+H | C35 H68 O3 N1 |
| 403 | POS119 | Cer(m19:0_17:0+O)+H | C36 H74 O3 N1 |
| 404 | POS120 | Cer(m19:1_17:0+O)+H | C36 H72 O3 N1 |
| 405 | POS121 | Cer(m20:1_18:4)+Na | C38 H67 O2 N1 Na1 |
| 406 | POS122 | Cer(m19:0_22:2)+H | C41 H80 O2 N1 |
| 407 | POS123 | Cer(m19:0_23:2)+H | C42 H82 O2 N1 |
| 408 | POS130 | Cer(t18:1_16:0+O)+H | C34 H68 O5 N1 |
| 409 | POS132 | Cer(t18:0_17:0+O)+H | C35 H72 O5 N1 |
| 410 | POS138 | Cer(t18:0_19:0+O)+H | C37 H76 O5 N1 |
| 411 | POS145 | Cer(t18:1_20:4)+H | C38 H68 O4 N1 |
| 412 | POS149 | Cer(t18:1_21:0+O)+H | C39 H78 O5 N1 |
| 413 | POS154 | Cer(t18:1_22:0+O)+H | C40 H80 O5 N1 |
| 414 | POS156 | Cer(t20:1_20:1+O)+H | C40 H78 O5 N1 |
| 415 | POS16 | Cer(d20:0_12:0)+H | C32 H66 O3 N1 |
| 416 | POS160 | Cer(t18:1_23:0+O)+H | C41 H82 O5 N1 |
| 417 | POS164 | Cer(t18:1_24:0)+H | C42 H84 O4 N1 |
| 418 | POS166 | Cer(t18:1_24:0+O)+H | C42 H84 O5 N1 |
| 419 | POS17 | Cer(d17:0_15:0+O)+H | C32 H66 O4 N1 |
| 420 | POS171 | Cer(t18:1_25:0+O)+H | C43 H86 O5 N1 |
| 421 | POS178 | Cer(t20:0_25:0)+H | C45 H92 O4 N1 |
| 422 | POS18 | Cer(d20:1_12:0)+H | C32 H64 O3 N1 |
| 423 | POS190 | CerP(d21:1_18:2+2O)+H | C39 H75 O8 N1 P1 |
| 424 | POS195 | DG(8:0e_6:0)+Na | C17 H34 O4 Na1 |
| 425 | POS199 | DG(8:0_11:2)+NH4 | C22 H42 O5 N1 |
| 426 | POS213 | DG(16:0_11:1)+NH4 | C30 H60 O5 N1 |
| 427 | POS215 | DG(16:1_11:4)+NH4 | C30 H52 O5 N1 |
| 428 | POS218 | DG(16:1_14:3)+NH4 | C33 H60 O5 N1 |
| 429 | POS22 | Cer(d18:2_15:0)+H | C33 H64 O3 N1 |
| 430 | POS220 | DG(18:1e_12:4)+NH4 | C33 H60 O4 N1 |
| 431 | POS223 | DG(11:0_20:1)+NH4 | C34 H68 O5 N1 |
| 432 | POS224 | DG(17:0_14:2)+NH4 | C34 H66 O5 N1 |
| 433 | POS23 | Cer(d15:2_18:0+2O)+H | C33 H64 O5 N1 |
| 434 | POS24 | Cer(d15:2_18:2+2O)+H | C33 H60 O5 N1 |
| 435 | POS243 | DG(14:0_18:3)+Na | C35 H62 O5 Na1 |
| 436 | POS244 | DG(12:0_20:3)+NH4 | C35 H66 O5 N1 |
| 437 | POS247 | DG(15:0_18:2)+NH4 | C36 H70 O5 N1 |
| 438 | POS276 | DG(18:4_16:1)+H | C37 H63 O5 |
| 439 | POS277 | DG(23:1_11:4)+NH4 | C37 H66 O5 N1 |
| 440 | POS286 | DG(18:1_18:3)+H | C39 H69 O5 |
| 441 | POS31 | Cer(d16:0_18:0+O)+H | C34 H70 O4 N1 |
| 442 | POS312 | DG(20:5_18:2)+H | C41 H67 O5 |
| 443 | POS313 | DG(19:0_20:1)+NH4 | C42 H84 O5 N1 |
| 444 | POS317 | DG(20:0_20:5)+H | C43 H75 O5 |
| 445 | POS325 | DG(25:0_18:2)+NH4 | C46 H90 O5 N1 |
| 446 | POS326 | DG(20:5_23:0)+H | C46 H81 O5 |
| 447 | POS33 | Cer(d16:0_18:1)+H | C34 H68 O3 N1 |
| 448 | POS36 | Cer(d18:1_16:0+O)+H | C34 H68 O4 N1 |
| 449 | POS386 | Hex1Cer(d16:0_16:0+O)+Na | C38 H75 O9 N1 Na1 |
| 450 | POS39 | Cer(d18:2_16:0+O)+H | C34 H66 O4 N1 |
| 451 | POS392 | Hex1Cer(d20:1_16:1)+H-H2O | C42 H78 O7 N1 |
| 452 | POS394 | Hex1Cer(d22:1_16:0)+H-H2O | C44 H84 O7 N1 |
| 453 | POS395 | Hex1Cer(d22:1_16:1)+H-H2O | C44 H82 O7 N1 |
| 454 | POS397 | Hex1Cer(d22:2_16:1)+H-H2O | C44 H80 O7 N1 |
| 455 | POS401 | Hex1Cer(d20:1_20:5)+H-H2O | C46 H78 O7 N1 |
| 456 | POS402 | Hex1Cer(d20:2_20:5)+H-H2O | C46 H76 O7 N1 |
| 457 | POS407 | Hex1Cer(d22:2_20:5)+H-H2O | C48 H80 O7 N1 |
| 458 | POS41 | Cer(d18:2_16:1)+H | C34 H64 O3 N1 |
| 459 | POS410 | Hex1Cer(m18:1_16:1)+H | C40 H76 O7 N1 |
| 460 | POS411 | Hex1Cer(m20:1_16:1)+H | C42 H80 O7 N1 |
| 461 | POS413 | Hex1Cer(m20:1_18:2)+H | C44 H82 O7 N1 |
| 462 | POS414 | Hex1Cer(m18:1_20:5)+H | C44 H76 O7 N1 |
| 463 | POS415 | Hex1Cer(m20:1_20:5)+H | C46 H80 O7 N1 |
| 464 | POS444 | Hex3Cer(d24:1_18:2+O)+NH4 | C60 H113 O19 N2 |
| 465 | POS45 | Cer(d18:2_16:1+O)+H | C34 H64 O4 N1 |
| 466 | POS450 | LPC(18:3)+H | C26 H49 O7 N1 P1 |
| 467 | POS457 | MGDG(16:0_16:1)+Na | C41 H76 O10 Na1 |
| 468 | POS459 | MGDG(16:0_16:3)+Na | C41 H72 O10 Na1 |
| 469 | POS46 | Cer(d18:1_17:0)+H | C35 H70 O3 N1 |
| 470 | POS463 | MGDG(16:0_18:1)+Na | C43 H80 O10 Na1 |
| 471 | POS465 | MGDG(16:0_18:2)+Na | C43 H78 O10 Na1 |
| 472 | POS467 | MGDG(16:0_18:3)+Na | C43 H76 O10 Na1 |
| 473 | POS469 | MGDG(18:2_16:2)+Na | C43 H74 O10 Na1 |
| 474 | POS47 | Cer(d21:1_14:0+O)+H | C35 H70 O4 N1 |
| 475 | POS471 | MGDG(18:3_16:2)+Na | C43 H72 O10 Na1 |
| 476 | POS473 | MGDG(14:0_20:5)+Na | C43 H72 O10 Na1 |
| 477 | POS475 | MGDG(18:3_16:3)+Na | C43 H70 O10 Na1 |
| 478 | POS478 | MGDG(18:0_18:2)+Na | C45 H82 O10 Na1 |
| 479 | POS48 | Cer(d18:1_17:1)+H | C35 H68 O3 N1 |
| 480 | POS480 | MGDG(18:1_18:2)+Na | C45 H80 O10 Na1 |
| 481 | POS482 | MGDG(18:2_18:2)+Na | C45 H78 O10 Na1 |
| 482 | POS483 | MGDG(18:2_18:3)+NH4 | C45 H80 O10 N1 |
| 483 | POS485 | MGDG(18:2_18:3)+Na | C45 H76 O10 Na1 |
| 484 | POS489 | MGDG(18:3_18:3)+Na | C45 H74 O10 Na1 |
| 485 | POS49 | Cer(d18:2_17:1)+H | C35 H66 O3 N1 |
| 486 | POS495 | MGDG(20:5_20:5)+Na | C49 H74 O10 Na1 |
| 487 | POS497 | MGDG(18:3_25:7)+Na | C52 H80 O10 Na1 |
| 488 | POS499 | MGDG(20:3_23:8)+Na | C52 H78 O10 Na1 |
| 489 | POS5 | Cer(d12:0_16:0)+H | C28 H58 O3 N1 |
| 490 | POS500 | MGDG(18:3_25:8)+Na | C52 H78 O10 Na1 |
| 491 | POS52 | Cer(d16:0_20:0+O)+H | C36 H74 O4 N1 |
| 492 | POS534 | PA(17:0_18:2)+NH4 | C38 H75 O8 N1 P1 |
| 493 | POS55 | Cer(d18:1_18:0+O)+H | C36 H72 O4 N1 |
| 494 | POS56 | Cer(d18:0_18:2)+H | C36 H70 O3 N1 |
| 495 | POS572 | PA(20:2_18:2)+NH4 | C41 H77 O8 N1 P1 |
| 496 | POS574 | PA(28:1_11:1)+NH4 | C42 H83 O8 N1 P1 |
| 497 | POS587 | PC(12:1e_11:4)+Na | C31 H54 O7 N1 P1 Na1 |
| 498 | POS59 | Cer(d18:1_18:1+O)+H | C36 H70 O4 N1 |
| 499 | POS590 | PC(6:0_24:1)+H | C38 H75 O8 N1 P1 |
| 500 | POS593 | PC(16:0_16:1)+H | C40 H79 O8 N1 P1 |
| 501 | POS594 | PC(18:0_14:1)+H | C40 H79 O8 N1 P1 |
| 502 | POS6 | Cer(d12:0_16:1)+H | C28 H56 O3 N1 |
| 503 | POS606 | PC(16:1_18:2)+H | C42 H79 O8 N1 P1 |
| 504 | POS608 | PC(16:1_18:3)+H | C42 H77 O8 N1 P1 |
| 505 | POS609 | PC(19:1_16:0)+H | C43 H85 O8 N1 P1 |
| 506 | POS610 | PC(17:1_18:1)+H | C43 H83 O8 N1 P1 |
| 507 | POS621 | PC(16:0_22:3)+H | C46 H87 O8 N1 P1 |
| 508 | POS630 | PE(17:0_11:4)+Na | C33 H58 O8 N1 P1 Na1 |
| 509 | POS64 | Cer(d18:2_18:2+O)+H | C36 H66 O4 N1 |
| 510 | POS65 | Cer(d18:2_18:3)+H | C36 H64 O3 N1 |
| 511 | POS67 | Cer(d18:0_20:0)+H | C38 H78 O3 N1 |
| 512 | POS680 | PE(19:1_18:2)+H | C42 H79 O8 N1 P1 |
| 513 | POS681 | PE(19:1_18:3)+H | C42 H77 O8 N1 P1 |
| 514 | POS684 | PE(26:0_12:3)+H | C43 H81 O8 N1 P1 |
| 515 | POS69 | Cer(d18:1_20:0)+H | C38 H76 O3 N1 |
| 516 | POS695 | PE(19:1_22:0)+H | C46 H91 O8 N1 P1 |
| 517 | POS71 | Cer(d18:2_21:0+O)+H | C39 H76 O4 N1 |
| 518 | POS718 | PG(30:0_8:0)+NH4 | C44 H91 O10 N1 P1 |
| 519 | POS719 | PG(19:1_20:3)+H | C45 H82 O10 N0 P1 |
| 520 | POS721 | PG(31:0_9:0)+NH4 | C46 H95 O10 N1 P1 |
| 521 | POS729 | PG(27:1_19:1)+H | C52 H100 O10 N0 P1 |
| 522 | POS737 | PI(16:0_18:3)+NH4 | C43 H81 O13 N1 P1 |
| 523 | POS74 | Cer(d18:0_22:0+O)+H | C40 H82 O4 N1 |
| 524 | POS747 | PI(19:1_18:3)+H | C46 H82 O13 N0 P1 |
| 525 | POS748 | PI(20:1_18:3)+H | C47 H84 O13 N0 P1 |
| 526 | POS75 | Cer(d22:1_18:0)+H | C40 H80 O3 N1 |
| 527 | POS750 | PI(16:0_22:4)+Na | C47 H83 O13 N0 P1 Na1 |
| 528 | POS752 | PI(18:3_20:2)+H | C47 H82 O13 N0 P1 |
| 529 | POS765 | PI(19:1_22:6)+H | C50 H84 O13 N0 P1 |
| 530 | POS767 | PI(35:1_16:0)+H | C60 H116 O13 N0 P1 |
| 531 | POS77 | Cer(d18:1_22:0+O)+H | C40 H80 O4 N1 |
| 532 | POS778 | SM(d18:2_24:0)+H | C47 H94 O6 N2 P1 |
| 533 | POS79 | Cer(d18:2_22:0+O)+H | C40 H78 O4 N1 |
| 534 | POS81 | Cer(d18:2_22:1)+H | C40 H76 O3 N1 |
| 535 | POS82 | Cer(d18:0_23:0)+H | C41 H84 O3 N1 |
| 536 | POS85 | Cer(d18:2_23:0+O)+H | C41 H80 O4 N1 |
| 537 | POS851 | SiE(18:2)+NH4 | C47 H84 O2 N1 |
| 538 | POS86 | Cer(d18:0_24:0)+H | C42 H86 O3 N1 |
| 539 | POS862 | TG(4:0_6:0_9:0)+H | C22 H41 O6 |
| 540 | POS866 | TG(4:0_6:0_11:2)+H | C24 H41 O6 |
| 541 | POS867 | TG(4:0_8:0_10:1)+NH4 | C25 H48 O6 N1 |
| 542 | POS870 | TG(4:0_9:0_18:3)+Na | C34 H58 O6 Na1 |
| 543 | POS879 | TG(12:0e_11:3_11:3)+NH4 | C37 H64 O5 N1 |
| 544 | POS882 | TG(15:0_6:0_15:0)+NH4 | C39 H78 O6 N1 |
| 545 | POS883 | TG(16:0_6:0_14:1)+NH4 | C39 H76 O6 N1 |
| 546 | POS886 | TG(18:0_6:0_12:2)+NH4 | C39 H74 O6 N1 |
| 547 | POS888 | TG(19:1_6:0_11:2)+NH4 | C39 H72 O6 N1 |
| 548 | POS889 | TG(16:0_8:0_12:4)+Na | C39 H66 O6 Na1 |
| 549 | POS89 | Cer(d18:1_24:0+O)+H | C42 H84 O4 N1 |
| 550 | POS892 | TG(12:1e_6:0_18:3)+H | C39 H69 O5 |
| 551 | POS895 | TG(15:0_6:0_16:0)+NH4 | C40 H80 O6 N1 |
| 552 | POS896 | TG(16:1_10:2_11:4)+H | C40 H63 O6 |
| 553 | POS897 | TG(4:0_15:0_19:0)+NH4 | C41 H82 O6 N1 |
| 554 | POS898 | TG(11:0_9:0_18:3)+NH4 | C41 H76 O6 N1 |
| 555 | POS9 | Cer(d14:0_16:1)+H | C30 H60 O3 N1 |
| 556 | POS901 | TG(16:1e_11:2_11:2)+NH4 | C41 H74 O5 N1 |
| 557 | POS902 | TG(16:2e_10:0_12:3)+H | C41 H71 O5 |
| 558 | POS906 | TG(15:0_8:0_16:0)+NH4 | C42 H84 O6 N1 |
| 559 | POS907 | TG(18:1e_10:4_11:3)+Na | C42 H66 O5 Na1 |
| 560 | POS908 | TG(16:1e_11:3_12:4)+Na | C42 H66 O5 Na1 |
| 561 | POS91 | Cer(d18:2_24:0)+H | C42 H82 O3 N1 |
| 562 | POS910 | TG(15:0_6:0_19:0)+NH4 | C43 H86 O6 N1 |
| 563 | POS911 | TG(11:0_15:0_15:0)+NH4 | C44 H88 O6 N1 |
| 564 | POS912 | TG(20:4e_10:2_11:4)+Na | C44 H66 O5 Na1 |
| 565 | POS913 | TG(18:3e_11:3_12:4)+Na | C44 H66 O5 Na1 |
| 566 | POS916 | TG(20:4e_10:2_12:4)+Na | C45 H68 O5 Na1 |
| 567 | POS917 | TG(20:3e_10:4_12:4)+Na | C45 H66 O5 Na1 |
| 568 | POS920 | TG(16:0e_12:0_16:1)+NH4 | C47 H94 O5 N1 |
| 569 | POS921 | TG(18:0e_10:4_16:1)+NH4 | C47 H86 O5 N1 |
| 570 | POS928 | TG(22:5_11:2_14:4)+NH4 | C50 H78 O6 N1 |
| 571 | POS931 | TG(22:5_11:4_14:3)+NH4 | C50 H76 O6 N1 |
| 572 | POS932 | TG(22:5_11:4_14:4)+NH4 | C50 H74 O6 N1 |
| 573 | POS94 | Cer(d18:2_24:0+O)+H | C42 H82 O4 N1 |
| 574 | POS96 | Cer(d18:2_24:1)+H | C42 H80 O3 N1 |
| 575 | POS97 | Cer(d18:0_25:0)+H | C43 H88 O3 N1 |
| 576 | NEG1 | CL(51:3)-H | C60 H109 O17 P2 |
| 577 | NEG104 | Cer(t36:1+O)-H | C36 H70 O5 N1 |
| 578 | NEG107 | Cer(t36:3)+HCOO | C37 H68 O6 N1 |
| 579 | NEG12 | CL(78:14)-2H | C87 H140 O17 P2 |
| 580 | NEG149 | Cer(t42:1+O)+HCOO | C43 H84 O7 N1 |
| 581 | NEG158 | Cer(t44:0)+HCOO | C45 H90 O6 N1 |
| 582 | NEG159 | Cer(t44:0+O)+HCOO | C45 H90 O7 N1 |
| 583 | NEG162 | Cer(t44:1+O)+HCOO | C45 H88 O7 N1 |
| 584 | NEG170 | CerG2GNAc1(d42:4)+HCOO | C63 H111 O20 N2 |
| 585 | NEG171 | CerG2GNAc1(d50:7)-H | C70 H119 O18 N2 |
| 586 | NEG172 | CerG2GNAc1(m37:0)-H | C57 H107 O17 N2 |
| 587 | NEG184 | DGDG(25:4)+HCOO | C41 H67 O17 |
| 588 | NEG237 | Hex1Cer(d34:1+2O)+HCOO | C41 H78 O12 N1 |
| 589 | NEG238 | Hex1Cer(d34:1+O)+HCOO | C41 H78 O11 N1 |
| 590 | NEG24 | Cer(d26:1)+HCOO | C27 H52 O5 N1 |
| 591 | NEG240 | Hex1Cer(d34:2)+HCOO | C41 H76 O10 N1 |
| 592 | NEG241 | Hex1Cer(d34:4+2O)+HCOO | C41 H72 O12 N1 |
| 593 | NEG242 | Hex1Cer(d35:4)+HCOO | C42 H74 O10 N1 |
| 594 | NEG243 | Hex1Cer(d36:2+O)+HCOO | C43 H80 O11 N1 |
| 595 | NEG248 | Hex1Cer(d38:1+O)+HCOO | C45 H86 O11 N1 |
| 596 | NEG25 | Cer(d26:2)+HCOO | C27 H50 O5 N1 |
| 597 | NEG252 | Hex1Cer(d40:1+O)+HCOO | C47 H90 O11 N1 |
| 598 | NEG256 | Hex1Cer(d42:1+O)+HCOO | C49 H94 O11 N1 |
| 599 | NEG257 | Hex1Cer(d42:2)+HCOO | C49 H92 O10 N1 |
| 600 | NEG258 | Hex1Cer(d42:2+2O)+HCOO | C49 H92 O12 N1 |
| 601 | NEG26 | Cer(d28:2)+HCOO | C29 H54 O5 N1 |
| 602 | NEG261 | Hex1Cer(d46:7)+HCOO | C53 H90 O10 N1 |
| 603 | NEG262 | Hex1Cer(d48:1+O)+HCOO | C55 H106 O11 N1 |
| 604 | NEG263 | Hex1Cer(m35:2)+HCOO | C42 H78 O9 N1 |
| 605 | NEG264 | Hex1Cer(m36:0)+HCOO | C43 H84 O9 N1 |
| 606 | NEG265 | Hex1Cer(m37:2)+HCOO | C44 H82 O9 N1 |
| 607 | NEG267 | Hex1Cer(m40:2)+HCOO | C47 H88 O9 N1 |
| 608 | NEG268 | Hex1Cer(t34:0+O)+HCOO | C41 H80 O12 N1 |
| 609 | NEG269 | Hex1Cer(t34:1)+HCOO | C41 H78 O11 N1 |
| 610 | NEG27 | Cer(d32:1+O)+HCOO | C33 H64 O6 N1 |
| 611 | NEG274 | Hex1Cer(t34:2)+HCOO | C41 H76 O11 N1 |
| 612 | NEG275 | Hex1Cer(t34:3)+HCOO | C41 H74 O11 N1 |
| 613 | NEG277 | Hex1Cer(t35:2)+HCOO | C42 H78 O11 N1 |
| 614 | NEG279 | Hex1Cer(t38:1+O)+HCOO | C45 H86 O12 N1 |
| 615 | NEG280 | Hex1Cer(t38:2)+HCOO | C45 H84 O11 N1 |
| 616 | NEG282 | Hex1Cer(t39:1)+HCOO | C46 H88 O11 N1 |
| 617 | NEG283 | Hex1Cer(t39:1+O)+HCOO | C46 H88 O12 N1 |
| 618 | NEG284 | Hex1Cer(t39:2)+HCOO | C46 H86 O11 N1 |
| 619 | NEG285 | Hex1Cer(t40:1)+HCOO | C47 H90 O11 N1 |
| 620 | NEG286 | Hex1Cer(t40:1+O)+HCOO | C47 H90 O12 N1 |
| 621 | NEG287 | Hex1Cer(t40:2)+HCOO | C47 H88 O11 N1 |
| 622 | NEG288 | Hex1Cer(t40:2+O)+HCOO | C47 H88 O12 N1 |
| 623 | NEG289 | Hex1Cer(t41:1)+HCOO | C48 H92 O11 N1 |
| 624 | NEG290 | Hex1Cer(t41:1+O)+HCOO | C48 H92 O12 N1 |
| 625 | NEG291 | Hex1Cer(t41:2)+HCOO | C48 H90 O11 N1 |
| 626 | NEG292 | Hex1Cer(t42:0+O)+HCOO | C49 H96 O12 N1 |
| 627 | NEG293 | Hex1Cer(t42:1)+HCOO | C49 H94 O11 N1 |
| 628 | NEG294 | Hex1Cer(t42:1+O)+HCOO | C49 H94 O12 N1 |
| 629 | NEG295 | Hex1Cer(t42:2)+HCOO | C49 H92 O11 N1 |
| 630 | NEG298 | Hex1Cer(t43:1)+HCOO | C50 H96 O11 N1 |
| 631 | NEG299 | Hex1Cer(t43:1+O)+HCOO | C50 H96 O12 N1 |
| 632 | NEG30 | Cer(d33:2+O)+HCOO | C34 H64 O6 N1 |
| 633 | NEG300 | Hex1Cer(t43:2)+HCOO | C50 H94 O11 N1 |
| 634 | NEG301 | Hex1Cer(t44:1)+HCOO | C51 H98 O11 N1 |
| 635 | NEG302 | Hex1Cer(t44:1+O)+HCOO | C51 H98 O12 N1 |
| 636 | NEG303 | Hex1Cer(t44:2)+HCOO | C51 H96 O11 N1 |
| 637 | NEG304 | Hex1Cer(t45:1+O)+HCOO | C52 H100 O12 N1 |
| 638 | NEG305 | Hex1Cer(t46:1+O)+HCOO | C53 H102 O12 N1 |
| 639 | NEG31 | Cer(d34:0)+HCOO | C35 H70 O5 N1 |
| 640 | NEG310 | Hex2Cer(m37:2)+HCOO | C50 H92 O14 N1 |
| 641 | NEG32 | Cer(d34:0+O)+HCOO | C35 H70 O6 N1 |
| 642 | NEG370 | OAHFA(40:6)-H | C40 H65 O4 |
| 643 | NEG372 | PA(31:4e)-H | C34 H60 O7 N0 P1 |
| 644 | NEG376 | PA(33:0)-H | C36 H70 O8 N0 P1 |
| 645 | NEG377 | PA(33:1)-H | C36 H68 O8 N0 P1 |
| 646 | NEG378 | PA(33:2)-H | C36 H66 O8 N0 P1 |
| 647 | NEG392 | PA(34:3)-H | C37 H66 O8 N0 P1 |
| 648 | NEG396 | PA(35:0)-H | C38 H74 O8 N0 P1 |
| 649 | NEG398 | PA(35:2)-H | C38 H70 O8 N0 P1 |
| 650 | NEG402 | PA(35:3)-H | C38 H68 O8 N0 P1 |
| 651 | NEG403 | PA(35:4)-H | C38 H66 O8 N0 P1 |
| 652 | NEG429 | PA(37:2)-H | C40 H74 O8 N0 P1 |
| 653 | NEG43 | Cer(d36:4)+HCOO | C37 H66 O5 N1 |
| 654 | NEG432 | PA(38:2)-H | C41 H76 O8 N0 P1 |
| 655 | NEG452 | PA(41:2)-H | C44 H82 O8 N0 P1 |
| 656 | NEG457 | PA(42:2)-H | C45 H84 O8 N0 P1 |
| 657 | NEG459 | PA(43:2)-H | C46 H86 O8 N0 P1 |
| 658 | NEG46 | Cer(d40:0)+HCOO | C41 H82 O5 N1 |
| 659 | NEG47 | Cer(d40:1)+HCOO | C41 H80 O5 N1 |
| 660 | NEG485 | PE(36:2e)-H | C41 H79 O7 N1 P1 |
| 661 | NEG487 | PE(36:3e)-H | C41 H77 O7 N1 P1 |
| 662 | NEG49 | Cer(d41:2)+HCOO | C42 H80 O5 N1 |
| 663 | NEG498 | PE(38:6e)-H | C43 H75 O7 N1 P1 |
| 664 | NEG499 | PE(38:7e)-H | C43 H73 O7 N1 P1 |
| 665 | NEG504 | PE(40:5e)-H | C45 H81 O7 N1 P1 |
| 666 | NEG507 | PE(40:8e)-H | C45 H75 O7 N1 P1 |
| 667 | NEG51 | Cer(d42:0+O)+HCOO | C43 H86 O6 N1 |
| 668 | NEG515 | PG(32:2)-H | C38 H70 O10 N0 P1 |
| 669 | NEG517 | PG(33:4)-H | C39 H68 O10 N0 P1 |
| 670 | NEG526 | PG(34:4)-H | C40 H70 O10 N0 P1 |
| 671 | NEG529 | PG(34:5)-H | C40 H68 O10 N0 P1 |
| 672 | NEG539 | PG(34:6)-H | C40 H66 O10 N0 P1 |
| 673 | NEG54 | Cer(d42:1+2O)+HCOO | C43 H84 O7 N1 |
| 674 | NEG544 | PG(36:5)-H | C42 H72 O10 N0 P1 |
| 675 | NEG547 | PG(36:7)-H | C42 H68 O10 N0 P1 |
| 676 | NEG557 | PG(36:8)-H | C42 H66 O10 N0 P1 |
| 677 | NEG558 | PG(40:5)-H | C46 H80 O10 N0 P1 |
| 678 | NEG56 | Cer(d42:2)+HCOO | C43 H82 O5 N1 |
| 679 | NEG561 | PG(42:2)-H | C48 H90 O10 N0 P1 |
| 680 | NEG564 | PI(32:3)-H | C41 H72 O13 N0 P1 |
| 681 | NEG58 | Cer(d43:1)+HCOO | C44 H86 O5 N1 |
| 682 | NEG582 | PIP(30:2e)-H | C39 H73 O15 N0 P2 |
| 683 | NEG584 | PIP(54:0)-H | C63 H123 O16 N0 P2 |
| 684 | NEG586 | PIP(56:2)-H | C65 H123 O16 N0 P2 |
| 685 | NEG587 | PIP2(44:4)-H | C53 H96 O19 N0 P3 |
| 686 | NEG588 | PIP2(62:0)-H | C71 H140 O19 N0 P3 |
| 687 | NEG589 | PS(36:3)-H | C42 H75 O10 N1 P1 |
| 688 | NEG59 | Cer(d44:1)+HCOO | C45 H88 O5 N1 |
| 689 | NEG590 | PS(36:4)-H | C42 H73 O10 N1 P1 |
| 690 | NEG593 | PS(38:4)-H | C44 H77 O10 N1 P1 |
| 691 | NEG594 | PS(38:5)-H | C44 H75 O10 N1 P1 |
| 692 | NEG595 | PS(38:6)-H | C44 H73 O10 N1 P1 |
| 693 | NEG60 | Cer(d44:1+O)+HCOO | C45 H88 O6 N1 |
| 694 | NEG601 | PS(40:6)-H | C46 H77 O10 N1 P1 |
| 695 | NEG605 | PS(42:3)-H | C48 H87 O10 N1 P1 |
| 696 | NEG606 | PS(42:8)-H | C48 H77 O10 N1 P1 |
| 697 | NEG607 | SM(d34:1)+HCOO | C40 H80 O8 N2 P1 |
| 698 | NEG608 | SM(d36:1)+HCOO | C42 H84 O8 N2 P1 |
| 699 | NEG609 | SM(d38:1)+HCOO | C44 H88 O8 N2 P1 |
| 700 | NEG610 | SM(d40:1)+HCOO | C46 H92 O8 N2 P1 |
| 701 | NEG611 | SM(d42:1)+HCOO | C48 H96 O8 N2 P1 |
| 702 | NEG612 | SQDG(30:0)-H | C39 H73 O12 S1 |
| 703 | NEG618 | SQDG(32:0)-H | C41 H77 O12 S1 |
| 704 | NEG62 | Cer(d45:1+O)+HCOO | C46 H90 O6 N1 |
| 705 | NEG620 | SQDG(32:2)-H | C41 H73 O12 S1 |
| 706 | NEG621 | SQDG(32:3)-H | C41 H71 O12 S1 |
| 707 | NEG622 | SQDG(33:0)-H | C42 H79 O12 S1 |
| 708 | NEG624 | SQDG(33:2)-H | C42 H75 O12 S1 |
| 709 | NEG626 | SQDG(33:3)-H | C42 H73 O12 S1 |
| 710 | NEG628 | SQDG(34:0)-H | C43 H81 O12 S1 |
| 711 | NEG629 | SQDG(34:1)-H | C43 H79 O12 S1 |
| 712 | NEG631 | SQDG(34:2)-H | C43 H77 O12 S1 |
| 713 | NEG632 | SQDG(34:3)-H | C43 H75 O12 S1 |
| 714 | NEG633 | SQDG(34:5)-H | C43 H71 O12 S1 |
| 715 | NEG634 | SQDG(35:2)-H | C44 H79 O12 S1 |
| 716 | NEG635 | SQDG(35:3)-H | C44 H77 O12 S1 |
| 717 | NEG636 | SQDG(36:1)-H | C45 H83 O12 S1 |
| 718 | NEG637 | SQDG(36:2)-H | C45 H81 O12 S1 |
| 719 | NEG638 | SQDG(36:3)-H | C45 H79 O12 S1 |
| 720 | NEG640 | SQDG(36:5)-H | C45 H75 O12 S1 |
| 721 | NEG656 | SQDG(51:1)-H | C60 H113 O12 S1 |
| 722 | NEG672 | ST(m44:4)-H | C50 H90 O10 N1 S1 |
| 723 | NEG673 | ST(m44:5)-H | C50 H88 O10 N1 S1 |
| 724 | NEG674 | ST(m46:5)-H | C52 H92 O10 N1 S1 |
| 725 | NEG675 | phSM(t36:3)-CH3 | C40 H76 O8 N2 P1 |
| 726 | NEG68 | Cer(m35:1+2O)+HCOO | C36 H70 O6 N1 |
| 727 | NEG71 | Cer(m39:1+O)+HCOO | C40 H78 O5 N1 |
| 728 | NEG73 | Cer(m42:0+O)+HCOO | C43 H86 O5 N1 |
| 729 | NEG74 | Cer(m42:1+O)+HCOO | C43 H84 O5 N1 |
| 730 | NEG81 | Cer(t32:1)+HCOO | C33 H64 O6 N1 |
| 731 | NEG90 | Cer(t34:1+O)+HCOO | C35 H68 O7 N1 |
| 732 | POS10 | Cer(d31:1)+Na | C31 H61 O3 N1 Na1 |
| 733 | POS1032 | WE(21:1)+NH4 | H44 C21 O2 N1 |
| 734 | POS1035 | WE(23:1)+NH4 | H48 C23 O2 N1 |
| 735 | POS1036 | WE(27:1)+NH4 | H56 C27 O2 N1 |
| 736 | POS1039 | ZyE(32:3)+NH4 | C59 H104 O2 N1 |
| 737 | POS1040 | ZyE(34:3)+NH4 | C61 H108 O2 N1 |
| 738 | POS124 | Cer(t32:0)+H | C32 H66 O4 N1 |
| 739 | POS125 | Cer(t34:0)+H | C34 H70 O4 N1 |
| 740 | POS13 | Cer(d31:1+O)+Na | C31 H61 O4 N1 Na1 |
| 741 | POS133 | Cer(t36:0)+H | C36 H74 O4 N1 |
| 742 | POS139 | Cer(t38:0)+H | C38 H78 O4 N1 |
| 743 | POS141 | Cer(t38:0+O)+H | C38 H78 O5 N1 |
| 744 | POS15 | Cer(d32:0)+H | C32 H66 O3 N1 |
| 745 | POS183 | CerG2GNAc1(d40:1)+H | C60 H113 O18 N2 |
| 746 | POS184 | CerG2GNAc1(d42:1)+Na | C62 H116 O18 N2 Na1 |
| 747 | POS185 | CerG2GNAc1(d42:1)+H | C62 H117 O18 N2 |
| 748 | POS186 | CerG2GNAc1(d42:2)+Na | C62 H114 O18 N2 Na1 |
| 749 | POS187 | CerG2GNAc1(d44:5)+NH4 | C64 H116 O18 N3 |
| 750 | POS188 | CerG2GNAc1(m32:0)+Na | C52 H98 O17 N2 Na1 |
| 751 | POS189 | CerG2GNAc1(m42:0+O)+NH4 | C62 H122 O18 N3 |
| 752 | POS19 | Cer(d33:0+O)+H-H2O | C33 H66 O3 N1 |
| 753 | POS191 | ChE(2:0)+H | C29 H49 O2 |
| 754 | POS196 | DG(18:3e)+H | C21 H37 O4 |
| 755 | POS197 | DG(19:1e)+H | C22 H43 O4 |
| 756 | POS20 | Cer(d33:1)+Na | C33 H65 O3 N1 Na1 |
| 757 | POS201 | DG(19:3e)+NH4 | C22 H42 O4 N1 |
| 758 | POS202 | DG(20:4e)+H | C23 H39 O4 |
| 759 | POS203 | DG(21:2)+NH4 | C24 H46 O5 N1 |
| 760 | POS204 | DG(21:4)+H | C24 H39 O5 |
| 761 | POS205 | DG(24:1)+NH4 | C27 H54 O5 N1 |
| 762 | POS207 | DG(24:2)+NH4 | C27 H52 O5 N1 |
| 763 | POS208 | DG(25:1)+NH4 | C28 H56 O5 N1 |
| 764 | POS209 | DG(25:2)+NH4 | C28 H54 O5 N1 |
| 765 | POS21 | Cer(d33:1+O)+Na | C33 H65 O4 N1 Na1 |
| 766 | POS210 | DG(25:3)+Na | C28 H48 O5 Na1 |
| 767 | POS211 | DG(26:2)+NH4 | C29 H56 O5 N1 |
| 768 | POS212 | DG(26:5)+NH4 | C29 H50 O5 N1 |
| 769 | POS214 | DG(27:2)+NH4 | C30 H58 O5 N1 |
| 770 | POS216 | DG(27:5)+NH4 | C30 H52 O5 N1 |
| 771 | POS217 | DG(30:0e)+Na | C33 H66 O4 Na1 |
| 772 | POS219 | DG(30:5)+NH4 | C33 H58 O5 N1 |
| 773 | POS221 | DG(30:7e)+NH4 | C33 H56 O4 N1 |
| 774 | POS225 | DG(32:0e)+Na | C35 H70 O4 Na1 |
| 775 | POS226 | DG(32:1e)+Na | C35 H68 O4 Na1 |
| 776 | POS238 | DG(32:2e)+Na | C35 H66 O4 Na1 |
| 777 | POS241 | DG(32:2e)+H | C35 H67 O4 |
| 778 | POS245 | DG(32:3e)+Na | C35 H64 O4 Na1 |
| 779 | POS246 | DG(33:1e)+Na | C36 H70 O4 Na1 |
| 780 | POS249 | DG(33:3e)+H | C36 H67 O4 |
| 781 | POS251 | DG(34:1e)+Na | C37 H72 O4 Na1 |
| 782 | POS261 | DG(34:3e)+Na | C37 H68 O4 Na1 |
| 783 | POS27 | Cer(d33:4+O)+H | C33 H60 O4 N1 |
| 784 | POS271 | DG(34:4e)+Na | C37 H66 O4 Na1 |
| 785 | POS272 | DG(34:4e)+H | C37 H67 O4 |
| 786 | POS278 | DG(34:5e)+H | C37 H65 O4 |
| 787 | POS279 | DG(34:5e)+Na | C37 H64 O4 Na1 |
| 788 | POS28 | Cer(d34:0)+H | C34 H70 O3 N1 |
| 789 | POS285 | DG(36:3e)+Na | C39 H72 O4 Na1 |
| 790 | POS290 | DG(36:4e)+H | C39 H71 O4 |
| 791 | POS298 | DG(36:5e)+H | C39 H69 O4 |
| 792 | POS305 | DG(37:2)+Na | C40 H74 O5 Na1 |
| 793 | POS306 | DG(38:1e)+Na | C41 H80 O4 Na1 |
| 794 | POS309 | DG(38:4)+NH4 | C41 H76 O5 N1 |
| 795 | POS316 | DG(40:4e)+H | C43 H79 O4 |
| 796 | POS318 | DG(40:6)+H | C43 H73 O5 |
| 797 | POS319 | DG(40:8)+Na | C43 H68 O5 Na1 |
| 798 | POS320 | DG(40:9)+Na | C43 H66 O5 Na1 |
| 799 | POS324 | DG(42:5)+H | C45 H79 O5 |
| 800 | POS327 | DGDG(18:2e)+NH4 | C33 H62 O14 N1 |
| 801 | POS328 | DGDG(18:3e)+NH4 | C33 H60 O14 N1 |
| 802 | POS329 | DGDG(18:3e)+H | C33 H57 O14 |
| 803 | POS330 | DGDG(20:5e)+H | C35 H57 O14 |
| 804 | POS331 | DGDG(20:6e)+H | C35 H55 O14 |
| 805 | POS371 | DGDG(37:6)+NH4 | C52 H90 O15 N1 |
| 806 | POS398 | Hex1Cer(d39:2+O)+H | C45 H86 O9 N1 |
| 807 | POS4 | Cer(d28:0)+H | C28 H58 O3 N1 |
| 808 | POS409 | Hex1Cer(m31:1)+H | C37 H72 O7 N1 |
| 809 | POS430 | Hex1Cer(t40:2+O)+H | C46 H88 O10 N1 |
| 810 | POS44 | Cer(d34:3+2O)+H-H2O | C34 H62 O4 N1 |
| 811 | POS455 | MGDG(18:3e)+NH4 | C27 H50 O9 N1 |
| 812 | POS51 | Cer(d36:0)+H | C36 H74 O3 N1 |
| 813 | POS513 | PA(34:2)+Na | C37 H69 O8 N0 P1 Na1 |
| 814 | POS559 | PA(36:5)+H | C39 H68 O8 N0 P1 |
| 815 | POS562 | PA(37:1e)+NH4 | C40 H83 O7 N1 P1 |
| 816 | POS565 | PA(38:2)+NH4 | C41 H81 O8 N1 P1 |
| 817 | POS571 | PA(38:2e)+Na | C41 H79 O7 N0 P1 Na1 |
| 818 | POS585 | PA(41:2)+NH4 | C44 H87 O8 N1 P1 |
| 819 | POS589 | PC(28:1)+H | C36 H71 O8 N1 P1 |
| 820 | POS591 | PC(31:2)+Na | C39 H74 O8 N1 P1 Na1 |
| 821 | POS596 | PC(33:1)+H | C41 H81 O8 N1 P1 |
| 822 | POS597 | PC(33:2)+H | C41 H79 O8 N1 P1 |
| 823 | POS60 | Cer(d36:3)+Na | C36 H67 O3 N1 Na1 |
| 824 | POS618 | PC(36:5)+H | C44 H79 O8 N1 P1 |
| 825 | POS620 | PC(36:6)+H | C44 H77 O8 N1 P1 |
| 826 | POS622 | PC(38:4)+H | C46 H85 O8 N1 P1 |
| 827 | POS628 | PE(18:2e)+H | C23 H45 O7 N1 P1 |
| 828 | POS631 | PE(31:0)+Na | C36 H72 O8 N1 P1 Na1 |
| 829 | POS632 | PE(31:0)+H | C36 H73 O8 N1 P1 |
| 830 | POS635 | PE(33:0)+H | C38 H77 O8 N1 P1 |
| 831 | POS637 | PE(33:1)+Na | C38 H74 O8 N1 P1 Na1 |
| 832 | POS638 | PE(33:1)+H | C38 H75 O8 N1 P1 |
| 833 | POS640 | PE(33:2)+H | C38 H73 O8 N1 P1 |
| 834 | POS642 | PE(33:3)+H | C38 H71 O8 N1 P1 |
| 835 | POS643 | PE(34:1)+H | C39 H77 O8 N1 P1 |
| 836 | POS648 | PE(35:0)+H | C40 H81 O8 N1 P1 |
| 837 | POS651 | PE(35:1)+H | C40 H79 O8 N1 P1 |
| 838 | POS653 | PE(35:2)+H | C40 H77 O8 N1 P1 |
| 839 | POS655 | PE(35:3)+H | C40 H75 O8 N1 P1 |
| 840 | POS658 | PE(35:4)+H | C40 H73 O8 N1 P1 |
| 841 | POS66 | Cer(d38:0)+H | C38 H78 O3 N1 |
| 842 | POS660 | PE(36:0)+H | C41 H83 O8 N1 P1 |
| 843 | POS661 | PE(36:1)+H | C41 H81 O8 N1 P1 |
| 844 | POS666 | PE(36:3)+H | C41 H77 O8 N1 P1 |
| 845 | POS676 | PE(37:1)+H | C42 H83 O8 N1 P1 |
| 846 | POS679 | PE(37:2)+H | C42 H81 O8 N1 P1 |
| 847 | POS68 | Cer(d38:1)+H | C38 H76 O3 N1 |
| 848 | POS682 | PE(38:1)+H | C43 H85 O8 N1 P1 |
| 849 | POS688 | PE(39:1)+H | C44 H87 O8 N1 P1 |
| 850 | POS690 | PE(39:3)+H | C44 H83 O8 N1 P1 |
| 851 | POS691 | PE(40:1)+H | C45 H89 O8 N1 P1 |
| 852 | POS696 | PE(41:1)+H | C46 H91 O8 N1 P1 |
| 853 | POS7 | Cer(d30:0)+H | C30 H62 O3 N1 |
| 854 | POS70 | Cer(d38:4)+H | C38 H70 O3 N1 |
| 855 | POS700 | PE(47:0)+Na | C52 H104 O8 N1 P1 Na1 |
| 856 | POS701 | PE(47:3)+H | C52 H99 O8 N1 P1 |
| 857 | POS703 | PG(27:0)+NH4 | C33 H69 O10 N1 P1 |
| 858 | POS704 | PG(29:2e)+NH4 | C35 H71 O9 N1 P1 |
| 859 | POS707 | PG(33:8)+NH4 | C39 H65 O10 N1 P1 |
| 860 | POS716 | PG(35:1)+Na | C41 H79 O10 N0 P1 Na1 |
| 861 | POS72 | Cer(d40:0)+H | C40 H82 O3 N1 |
| 862 | POS722 | PG(41:1)+H | C47 H92 O10 N0 P1 |
| 863 | POS723 | PG(42:0)+H | C48 H96 O10 N0 P1 |
| 864 | POS724 | PG(42:1)+H | C48 H94 O10 N0 P1 |
| 865 | POS725 | PG(42:2)+H | C48 H92 O10 N0 P1 |
| 866 | POS726 | PG(44:0)+H | C50 H100 O10 N0 P1 |
| 867 | POS727 | PG(46:1)+H | C52 H102 O10 N0 P1 |
| 868 | POS730 | PI(29:2e)+NH4 | C38 H75 O12 N1 P1 |
| 869 | POS731 | PI(31:2e)+NH4 | C40 H79 O12 N1 P1 |
| 870 | POS732 | PI(31:4e)+NH4 | C40 H75 O12 N1 P1 |
| 871 | POS733 | PI(32:2e)+NH4 | C41 H81 O12 N1 P1 |
| 872 | POS741 | PI(36:1)+Na | C45 H85 O13 N0 P1 Na1 |
| 873 | POS753 | PI(38:5)+Na | C47 H81 O13 N0 P1 Na1 |
| 874 | POS755 | PI(38:5e)+H | C47 H84 O12 N0 P1 |
| 875 | POS757 | PI(38:5e)+Na | C47 H83 O12 N0 P1 Na1 |
| 876 | POS762 | PI(38:6e)+H | C47 H82 O12 N0 P1 |
| 877 | POS763 | PI(39:5)+H | C48 H84 O13 N0 P1 |
| 878 | POS764 | PI(40:3)+H | C49 H90 O13 N0 P1 |
| 879 | POS768 | PIP(34:1e)+Na | C43 H84 O15 N0 P2 Na1 |
| 880 | POS769 | PS(29:4e)+Na | C35 H62 O9 N1 P1 Na1 |
| 881 | POS771 | PS(37:1)+H | C43 H83 O10 N1 P1 |
| 882 | POS772 | PS(39:4)+H | C45 H81 O10 N1 P1 |
| 883 | POS775 | PS(49:4)+Na | C55 H100 O10 N1 P1 Na1 |
| 884 | POS790 | SPH(d18:0)+H | C18 H40 O2 N1 |
| 885 | POS791 | SPH(d18:1)+H | C18 H38 O2 N1 |
| 886 | POS792 | SPH(d18:2)+H | C18 H36 O2 N1 |
| 887 | POS797 | SPH(d20:2)+H | C20 H40 O2 N1 |
| 888 | POS8 | Cer(d30:0+O)+H | C30 H62 O4 N1 |
| 889 | POS807 | SPH(t16:0)+H | C16 H36 O3 N1 |
| 890 | POS808 | SPH(t17:1)+H | C17 H36 O3 N1 |
| 891 | POS809 | SPH(t18:0)+H | C18 H40 O3 N1 |
| 892 | POS811 | SPH(t18:1)+H | C18 H38 O3 N1 |
| 893 | POS827 | SQDG(34:0)+NH4 | C43 H86 O12 S1 N1 |
| 894 | POS840 | ST(m36:3)+H-H2O | C42 H76 O9 N1 S1 |
| 895 | POS859 | StE(24:7)+NH4 | C53 H84 O2 N1 |
| 896 | POS864 | TG(21:0)+H | C24 H45 O6 |
| 897 | POS869 | TG(28:4)+H | C31 H51 O6 |
| 898 | POS880 | TG(34:7)+H | C37 H57 O6 |
| 899 | POS894 | TG(36:5)+Na | C39 H64 O6 Na1 |
| 900 | POS899 | TG(38:3)+NH4 | C41 H76 O6 N1 |
| 901 | POS900 | TG(38:4)+NH4 | C41 H74 O6 N1 |
| 902 | POS905 | TG(38:7)+H | C41 H65 O6 |
| 903 | POS914 | TG(41:2e)+NH4 | C44 H86 O5 N1 |
| 904 | POS922 | TG(44:6e)+NH4 | C47 H84 O5 N1 |
| 905 | POS929 | TG(47:11)+NH4 | C50 H78 O6 N1 |
| 906 | POS930 | TG(47:12)+NH4 | C50 H76 O6 N1 |
| 907 | POS940 | TG(49:12)+NH4 | C52 H80 O6 N1 |
| 908 | NEG10 | CL(75:6)-2H | C84 H150 O17 P2 |
| 909 | NEG11 | CL(76:9)-H | C85 H147 O17 P2 |
| 910 | NEG124 | Cer(t40:0)+HCOO | C41 H82 O6 N1 |
| 911 | NEG13 | CL(78:18)-2H | C87 H132 O17 P2 |
| 912 | NEG137 | Cer(t41:1+O)+HCOO | C42 H82 O7 N1 |
| 913 | NEG14 | CL(80:14)-2H | C89 H144 O17 P2 |
| 914 | NEG15 | CL(81:11)-2H | C90 H152 O17 P2 |
| 915 | NEG156 | Cer(t43:1+O)+HCOO | C44 H86 O7 N1 |
| 916 | NEG16 | CL(81:14)-H | C90 H147 O17 P2 |
| 917 | NEG165 | Cer(t45:1+O)+HCOO | C46 H90 O7 N1 |
| 918 | NEG166 | Cer(t46:0+O)+HCOO | C47 H94 O7 N1 |
| 919 | NEG167 | Cer(t46:1)+HCOO | C47 H92 O6 N1 |
| 920 | NEG168 | Cer(t46:1+O)+HCOO | C47 H92 O7 N1 |
| 921 | NEG17 | CL(83:15)-2H | C92 H148 O17 P2 |
| 922 | NEG173 | CerP(m35:0)-H | C35 H71 O5 N1 P1 |
| 923 | NEG174 | CerP(m35:0+O)-H | C35 H71 O6 N1 P1 |
| 924 | NEG175 | CerP(m38:0)+HCOO | C39 H79 O7 N1 P1 |
| 925 | NEG176 | CerP(m41:0+O)-H | C41 H83 O6 N1 P1 |
| 926 | NEG177 | CerP(t33:2+2O)-H | C33 H63 O9 N1 P1 |
| 927 | NEG178 | CerP(t39:2)+HCOO | C40 H77 O9 N1 P1 |
| 928 | NEG179 | CerP(t40:2)+HCOO | C41 H79 O9 N1 P1 |
| 929 | NEG18 | CL(83:17)-2H | C92 H144 O17 P2 |
| 930 | NEG180 | CerP(t42:2)+HCOO | C43 H83 O9 N1 P1 |
| 931 | NEG181 | DGDG(24:2)-H | C39 H67 O15 |
| 932 | NEG182 | DGDG(24:2)+HCOO | C40 H69 O17 |
| 933 | NEG186 | DGDG(26:2)+HCOO | C42 H73 O17 |
| 934 | NEG187 | DGDG(28:0)-H | C43 H79 O15 |
| 935 | NEG188 | DGDG(30:0)-H | C45 H83 O15 |
| 936 | NEG189 | DGDG(30:2)+HCOO | C46 H81 O17 |
| 937 | NEG19 | CL(83:9)-2H | C92 H160 O17 P2 |
| 938 | NEG190 | DGDG(31:3e)+HCOO | C47 H83 O16 |
| 939 | NEG192 | DGDG(32:0)-H | C47 H87 O15 |
| 940 | NEG194 | DGDG(32:3)-H | C47 H81 O15 |
| 941 | NEG195 | DGDG(32:6)-H | C47 H75 O15 |
| 942 | NEG198 | DGDG(34:2)+HCOO | C50 H89 O17 |
| 943 | NEG2 | CL(55:5)-2H | C64 H112 O17 P2 |
| 944 | NEG20 | CL(84:18)-2H | C93 H144 O17 P2 |
| 945 | NEG204 | DGDG(34:4)-H | C49 H83 O15 |
| 946 | NEG208 | DGDG(34:6)-H | C49 H79 O15 |
| 947 | NEG22 | CL(85:18)-H | C94 H147 O17 P2 |
| 948 | NEG225 | DGDG(42:5)+HCOO | C58 H99 O17 |
| 949 | NEG227 | DGDG(55:8)-H | C70 H117 O15 |
| 950 | NEG228 | DGDG(55:9)-H | C70 H115 O15 |
| 951 | NEG23 | CL(87:9)-2H | C96 H168 O17 P2 |
| 952 | NEG235 | GM3(d27:1)-H | C50 H89 O21 N2 |
| 953 | NEG236 | GM3(t39:6)-2H | C62 H102 O22 N2 |
| 954 | NEG244 | Hex1Cer(d37:1)+HCOO | C44 H84 O10 N1 |
| 955 | NEG245 | Hex1Cer(d37:3)+HCOO | C44 H80 O10 N1 |
| 956 | NEG247 | Hex1Cer(d38:1)+HCOO | C45 H86 O10 N1 |
| 957 | NEG249 | Hex1Cer(d39:1)+HCOO | C46 H88 O10 N1 |
| 958 | NEG250 | Hex1Cer(d39:3)-H | C45 H82 O8 N1 |
| 959 | NEG251 | Hex1Cer(d40:1)+HCOO | C47 H90 O10 N1 |
| 960 | NEG253 | Hex1Cer(d40:3)+HCOO | C47 H86 O10 N1 |
| 961 | NEG254 | Hex1Cer(d41:1)+HCOO | C48 H92 O10 N1 |
| 962 | NEG255 | Hex1Cer(d42:1)+HCOO | C49 H94 O10 N1 |
| 963 | NEG259 | Hex1Cer(d42:3)-H | C48 H88 O8 N1 |
| 964 | NEG260 | Hex1Cer(d43:1)+HCOO | C50 H96 O10 N1 |
| 965 | NEG266 | Hex1Cer(m39:1)+HCOO | C46 H88 O9 N1 |
| 966 | NEG271 | Hex1Cer(t34:1)-H | C40 H76 O9 N1 |
| 967 | NEG276 | Hex1Cer(t34:3)-H | C40 H72 O9 N1 |
| 968 | NEG278 | Hex1Cer(t37:3)-H | C43 H78 O9 N1 |
| 969 | NEG281 | Hex1Cer(t38:3)-H | C44 H80 O9 N1 |
| 970 | NEG297 | Hex1Cer(t42:2)-H | C48 H90 O9 N1 |
| 971 | NEG3 | CL(68:4)-2H | C77 H140 O17 P2 |
| 972 | NEG306 | Hex2Cer(d32:3)-H | C44 H78 O13 N1 |
| 973 | NEG307 | Hex2Cer(d32:4)-H | C44 H76 O13 N1 |
| 974 | NEG308 | Hex2Cer(d40:3)-H | C52 H94 O13 N1 |
| 975 | NEG309 | Hex2Cer(d41:4)-H | C53 H94 O13 N1 |
| 976 | NEG311 | Hex2Cer(t42:6)+HCOO | C55 H94 O16 N1 |
| 977 | NEG328 | MGDG(18:0)+HCOO | C28 H51 O12 |
| 978 | NEG329 | MGDG(20:2)+HCOO | C30 H51 O12 |
| 979 | NEG330 | MGDG(20:3)+HCOO | C30 H49 O12 |
| 980 | NEG331 | MGDG(25:8)-H | C34 H47 O10 |
| 981 | NEG332 | MGDG(27:5)+HCOO | C37 H59 O12 |
| 982 | NEG337 | MGDG(33:3)+HCOO | C43 H75 O12 |
| 983 | NEG348 | MGDG(35:4)-H | C44 H75 O10 |
| 984 | NEG35 | Cer(d34:1+2O)+HCOO | C35 H68 O7 N1 |
| 985 | NEG359 | MGDG(43:9)+HCOO | C53 H83 O12 |
| 986 | NEG360 | MGDG(45:8)+HCOO | C55 H89 O12 |
| 987 | NEG362 | MGDG(45:9)+HCOO | C55 H87 O12 |
| 988 | NEG42 | Cer(d36:3+2O)+HCOO | C37 H68 O7 N1 |
| 989 | NEG44 | Cer(d37:1)+HCOO | C38 H74 O5 N1 |
| 990 | NEG5 | CL(70:14)-2H | C79 H124 O17 P2 |
| 991 | NEG50 | Cer(d41:2+2O)+HCOO | C42 H80 O7 N1 |
| 992 | NEG55 | Cer(d42:1+O)+HCOO | C43 H84 O6 N1 |
| 993 | NEG57 | Cer(d42:2+2O)+HCOO | C43 H82 O7 N1 |
| 994 | NEG578 | PI(44:1)-H | C53 H100 O13 N0 P1 |
| 995 | NEG579 | PI(44:2)-H | C53 H98 O13 N0 P1 |
| 996 | NEG580 | PI(46:4)-H | C55 H98 O13 N0 P1 |
| 997 | NEG581 | PIP(20:2e)-H | C29 H53 O15 N0 P2 |
| 998 | NEG583 | PIP(40:1)-H | C49 H93 O16 N0 P2 |
| 999 | NEG585 | PIP(54:6)-H | C63 H111 O16 N0 P2 |
| 1000 | NEG61 | Cer(d44:2)+HCOO | C45 H86 O5 N1 |
| 1001 | NEG613 | SQDG(30:0ep)+HCOO | C40 H77 O12 S1 |
| 1002 | NEG615 | SQDG(31:2e)+HCOO | C41 H75 O13 S1 |
| 1003 | NEG619 | SQDG(32:0ep)+HCOO | C42 H81 O12 S1 |
| 1004 | NEG623 | SQDG(33:1e)+HCOO | C43 H81 O13 S1 |
| 1005 | NEG625 | SQDG(33:2e)+HCOO | C43 H79 O13 S1 |
| 1006 | NEG627 | SQDG(33:3e)+HCOO | C43 H77 O13 S1 |
| 1007 | NEG63 | Cer(d46:0+O)+HCOO | C47 H94 O6 N1 |
| 1008 | NEG630 | SQDG(34:1ep)+HCOO | C44 H83 O12 S1 |
| 1009 | NEG64 | Cer(d48:0+O)+HCOO | C49 H98 O6 N1 |
| 1010 | NEG642 | SQDG(38:7)+HCOO | C48 H77 O14 S1 |
| 1011 | NEG645 | SQDG(40:8)+HCOO | C50 H79 O14 S1 |
| 1012 | NEG646 | SQDG(42:5e)+HCOO | C52 H91 O13 S1 |
| 1013 | NEG647 | SQDG(42:8e)+HCOO | C52 H85 O13 S1 |
| 1014 | NEG648 | SQDG(45:12e)-H | C54 H81 O11 S1 |
| 1015 | NEG649 | SQDG(47:0)-H | C56 H107 O12 S1 |
| 1016 | NEG65 | Cer(d48:1+O)+HCOO | C49 H96 O6 N1 |
| 1017 | NEG650 | SQDG(48:0)-H | C57 H109 O12 S1 |
| 1018 | NEG651 | SQDG(49:0)-H | C58 H111 O12 S1 |
| 1019 | NEG652 | SQDG(49:1)-H | C58 H109 O12 S1 |
| 1020 | NEG653 | SQDG(49:1)+HCOO | C59 H111 O14 S1 |
| 1021 | NEG654 | SQDG(49:2)-H | C58 H107 O12 S1 |
| 1022 | NEG655 | SQDG(51:0)-H | C60 H115 O12 S1 |
| 1023 | NEG657 | SQDG(51:2)-H | C60 H111 O12 S1 |
| 1024 | NEG658 | SQDG(51:3)-H | C60 H109 O12 S1 |
| 1025 | NEG66 | Cer(d50:1+O)+HCOO | C51 H100 O6 N1 |
| 1026 | NEG662 | SQMG(18:1)-H | C27 H49 O11 S1 |
| 1027 | NEG663 | SQMG(18:2)-H | C27 H47 O11 S1 |
| 1028 | NEG664 | SQMG(18:3)-H | C27 H45 O11 S1 |
| 1029 | NEG67 | Cer(m34:0+O)+HCOO | C35 H70 O5 N1 |
| 1030 | NEG69 | Cer(m36:0+O)+HCOO | C37 H74 O5 N1 |
| 1031 | NEG70 | Cer(m38:0+O)+HCOO | C39 H78 O5 N1 |
| 1032 | NEG72 | Cer(m41:0+O)+HCOO | C42 H84 O5 N1 |
| 1033 | NEG75 | Cer(m43:0+O)+HCOO | C44 H88 O5 N1 |
| 1034 | NEG76 | Cer(m44:0+O)+HCOO | C45 H90 O5 N1 |
| 1035 | NEG77 | Cer(m45:1+O)+HCOO | C46 H90 O5 N1 |
| 1036 | NEG78 | Cer(m46:0+O)+HCOO | C47 H94 O5 N1 |
| 1037 | NEG79 | Cer(m48:1+O)+HCOO | C49 H96 O5 N1 |
| 1038 | NEG80 | Cer(t32:0)+HCOO | C33 H66 O6 N1 |
| 1039 | NEG82 | Cer(t32:1+2O)+HCOO | C33 H64 O8 N1 |
| 1040 | NEG85 | Cer(t34:0)+HCOO | C35 H70 O6 N1 |
| 1041 | NEG99 | Cer(t35:5)+HCOO | C36 H62 O6 N1 |
| 1042 | POS1004 | TG(56:12)+NH4 | C59 H94 O6 N1 |
| 1043 | POS1016 | TG(58:12)+NH4 | C61 H98 O6 N1 |
| 1044 | POS179 | Cer(t45:0+O)+H | C45 H92 O5 N1 |
| 1045 | POS26 | Cer(d33:4+2O)+H | C33 H60 O5 N1 |
| 1046 | POS332 | DGDG(30:4e)+Na | C45 H78 O14 Na1 |
| 1047 | POS358 | DGDG(36:2)+NH4 | C51 H96 O15 N1 |
| 1048 | POS372 | DGDG(38:2)+NH4 | C53 H100 O15 N1 |
| 1049 | POS375 | DGDG(38:4)+NH4 | C53 H96 O15 N1 |
| 1050 | POS376 | DGDG(38:5)+NH4 | C53 H94 O15 N1 |
| 1051 | POS377 | DGDG(38:6)+NH4 | C53 H92 O15 N1 |
| 1052 | POS385 | Hex1Cer(d28:1+O)+Na | C34 H65 O9 N1 Na1 |
| 1053 | POS416 | Hex1Cer(m42:5)+H | C48 H86 O7 N1 |
| 1054 | POS417 | Hex1Cer(m46:5)+H | C52 H94 O7 N1 |
| 1055 | POS442 | Hex2Cer(d30:3)+NH4 | C42 H79 O13 N2 |
| 1056 | POS443 | Hex2Cer(d31:2)+H-H2O | C43 H78 O12 N1 |
| 1057 | POS445 | Hex3Cer(m40:1+2O)+NH4 | C58 H113 O19 N2 |
| 1058 | POS453 | MG(19:1)+H | C22 H43 O4 |
| 1059 | POS460 | MGDG(33:3)+NH4 | C42 H78 O10 N1 |
| 1060 | POS461 | MGDG(33:5)+NH4 | C42 H74 O10 N1 |
| 1061 | POS476 | MGDG(36:2)+NH4 | C45 H86 O10 N1 |
| 1062 | POS490 | MGDG(38:3)+NH4 | C47 H88 O10 N1 |
| 1063 | POS491 | MGDG(38:4)+NH4 | C47 H86 O10 N1 |
| 1064 | POS492 | MGDG(38:5)+NH4 | C47 H84 O10 N1 |
| 1065 | POS494 | MGDG(38:6)+NH4 | C47 H82 O10 N1 |
| 1066 | POS496 | MGDG(42:10)+NH4 | C51 H82 O10 N1 |
| 1067 | POS498 | MGDG(43:10)+Na | C52 H80 O10 Na1 |
| 1068 | POS50 | Cer(d35:4)+H | C35 H64 O3 N1 |
| 1069 | POS503 | MGDG(43:13e)+Na | C52 H76 O9 Na1 |
| 1070 | POS504 | MGDG(44:10)+Na | C53 H82 O10 Na1 |
| 1071 | POS505 | MGDG(47:12)+Na | C56 H84 O10 Na1 |
| 1072 | POS61 | Cer(d36:3+O)+H | C36 H68 O4 N1 |
| 1073 | POS627 | PE(16:1e)+H | C21 H43 O7 N1 P1 |
| 1074 | POS633 | PE(32:0)+H | C37 H75 O8 N1 P1 |
| 1075 | POS636 | PE(33:0e)+H | C38 H79 O7 N1 P1 |
| 1076 | POS659 | PE(35:5)+H | C40 H71 O8 N1 P1 |
| 1077 | POS699 | PE(42:3e)+H | C47 H91 O7 N1 P1 |
| 1078 | POS702 | PE(57:7)+Na | C62 H110 O8 N1 P1 Na1 |
| 1079 | POS779 | SPH(d12:0)+H | C12 H28 O2 N1 |
| 1080 | POS780 | SPH(d14:0)+H | C14 H32 O2 N1 |
| 1081 | POS784 | SPH(d16:0)+H | C16 H36 O2 N1 |
| 1082 | POS788 | SPH(d16:1)+H | C16 H34 O2 N1 |
| 1083 | POS795 | SPH(d20:0)+H | C20 H44 O2 N1 |
| 1084 | POS798 | SPH(d22:0)+H | C22 H48 O2 N1 |
| 1085 | POS801 | SPH(d22:1)+H | C22 H46 O2 N1 |
| 1086 | POS803 | SPH(d22:2)+H | C22 H44 O2 N1 |
| 1087 | POS804 | SPH(m18:0)+H | C18 H40 O1 N1 |
| 1088 | POS805 | SPH(m20:0)+H | C20 H44 O1 N1 |
| 1089 | POS806 | SPH(m20:1)+H | C20 H42 O1 N1 |
| 1090 | POS839 | SQDG(47:2)+NH4 | C56 H108 O12 S1 N1 |
| 1091 | POS841 | ST(m43:5)+H-H2O | C49 H86 O9 N1 S1 |
| 1092 | POS842 | ST(t31:0)+NH4 | C37 H77 O12 N2 S1 |
| 1093 | POS881 | TG(35:4e)+NH4 | C38 H70 O5 N1 |
| 1094 | POS884 | TG(36:1)+NH4 | C39 H76 O6 N1 |
| 1095 | POS885 | TG(36:2)+NH4 | C39 H74 O6 N1 |
| 1096 | POS918 | TG(42:2)+NH4 | C45 H86 O6 N1 |

Table S5

**Table S5. List of primers**

| No. | Gene | Forward | Reverse |
| --- | --- | --- | --- |
| 1 | *Cd74* | 5′- GCTGGATGAAGCAGTGGCTCTT-3′ | 5′-GATGTGGCTGACTTCTCCTGG-3′ |
| 2 | *Rpl5* | 5′- GCGCTACCTAATGGAGGAAGATG-3′ | 5′-CTCTCGGATAGCAGCATGAGCT-3′ |
| 3 | *Satb1* | 5′- TCACAGGCAGTATTTGCACGCG-3′ | 5′-CGAAGGTTTACCAGCAGAGACTG-3′ |
| 4 | *Foxp1* | 5′- CATGCCTCTACCAATGGACAGC-3′ | 5′-GAAGTCGTCACAAACCGCCTCA-3′ |
| 5 | *Lars2* | 5′- GCCTTCCATCTGGACAGTGTCT-3′ | 5′-GGACTTGCTCATCTTCTCCCAC-3′ |
| 6 | *Rpl36* | 5′- GTCAGTAAGCCGAGACACAGCC-3′ | 5′-CCTTGGACACTTTGAGCAACTCC-3′ |
| 7 | *Gphn* | 5′- CTTCCGTCGTGCTCATCTACCT-3′ | 5′-CATGCGATGTCTTCTAGCCACC-3′ |
| 8 | *Peak1* | 5′- CAAGATGGCACTTCAGTCACACC-3′ | 5′-CGTGGATGCTTGGGAGGTATCA-3′ |
| 9 | *Mef2c* | 5′- GTGGTTTCCGTAGCAACTCCTAC-3′ | 5′-GGCAGTGTTGAAGCCAGACAGA-3′ |
| 10 | *Rpl23a* | 5′- AGCATCAGATCAAACAGGCTGTC-3′ | 5′-TCAGGAGCCAAGCGAACATACG-3′ |
| 11 | *Cd83* | 5′- ACCGTGGTTCTGAAGGTGACAG-3′ | 5′-CCAGAGAGAAGAGCAACACAGC-3′ |
| 12 | *Bank1* | 5′- GGCAGCTAGAATGAAGGCGACA-3′ | 5′-GCTATTTCTGCCAGTGTTTTGGC-3′ |
| 13 | *β-actin* | 5′- CATGTACGTTGCTATCCAGGC-3′ | 5′-CTCCTTAATGTCACGCACGAT-3′ |

**Video S1.** **An representative video of Nanoparticle Tracking Analysis (NTA) for Centella Asiatica-derived exosomes (CAEs).**
